# Trends and cross-country inequalities in the global burden of Alzheimer’s disease and other dementias among adults aged 65+ years, 1990–2021: a population based study with projections into 2050

**DOI:** 10.3389/fnagi.2025.1683158

**Published:** 2026-01-29

**Authors:** Sibo Liu, Guozhi Wu, Xiao Ma, Kai Xu, Xiaoyan Lan, Long Wang, Hang Li, Dean Gu, Mengxing Wang, Jinjie Liu

**Affiliations:** 1 Department of Intensive Care Unit, Central Hospital of Dalian University of Technology, Dalian, China; 2 Department of General Medicine, Central Hospital of Dalian University of Technology, Dalian, China; 3 Department of Neurosurgery, Central Hospital of Dalian University of Technology, Dalian, China; 4 Department of Neurology, Central Hospital of Dalian University of Technology, Dalian, China; 5 School of Computer and Communication Engineering, University of Science and Technology Beijing, Beijing, China; 6 Department of Geriatrics, Affiliated Dalian Friendship Hospital of Dalian Medical University, Dalian, China; 7 Department of Neurology, Qingdao Central Hospital, University of Health and Rehabilitation Sciences, Qingdao, China; 8 China National Clinical Research Center for Neurological Diseases, Beijing, China; 9 Department of Scientific Research and Discipline Building, Central Hospital of Dalian University of Technology, Dalian, China; 10 Department of Neurology, Beijing Tiantan Hospital, Capital Medical University, Beijing, China

**Keywords:** Alzheimer’s disease, average annual percentage change, dementia, disability-adjusted life years, mortality, prevalence, risk factors

## Abstract

**Background:**

Alzheimer’s disease and other dementias (ADOD) pose a significant global health challenge, with projected annual increases. The growing elderly population exacerbates burdens, underscoring the need for interventions.

**Methods:**

Using data from the Global Burden of Disease Study 2021, we analysed global ADOD trends from 1990 to 2021 among adults aged 65+ and projected to 2050. Average annual percentage change (AAPC) in the age-standardised prevalence, mortality and disability-adjusted life years (DALYs) rates of ADOD were calculated to quantify the temporal trends.

**Results:**

Globally, the number of adults aged 65 years or older living with ADOD increased from 18.1 (95% uncertainty interval [UI] 14.4–22.5) million in 1990 to 49.1 (38.7–61.3) million in 2021 (AAPC 0.09%). While mortality rates remained relatively stable (AAPC 0%), DALYs increased by 176% (AAPC 0.02%). Females consistently exhibited a higher age-standardised prevalence (7,603 [95% UI 6,023–9,469] cases per 100,000 population) vs. 5,744 [4,486–7,205]) and mortality (304 [78 to 782] vs. 225 [54–626]) rate compared to males, with notable regional variations. From 1990 to 2021, the age-standardised prevalence of ADOD increased only in East Asia and High-income Asia Pacific (AAPC 0.79% and 0.16%). High fasting plasma glucose, high body-mass index, and smoking were identified as primary risk factors. Projections indicate a 50.1% increase in age-standardised prevalence by 2050, with an estimated 191 (52–330) million cases, and a predicted death toll of 6.8 (2.4–11.3) million.

**Conclusion:**

The global number of people living with dementia nearly tripled from 1990 to 2021, mainly due to increases in population ageing and growth. The findings emphasize the need for comprehensive strategies to address ADOD, including prevention, early diagnosis, and effective management, with a focus on gender and regional disparities.

## 1 Introduction

Alzheimer’s disease and other dementias (ADOD) represent a significant global health challenge, with the growing aging population expected to exacerbate associated healthcare and societal burdens (World Health Organization [WHO], 2023). The impact of ADOD extends beyond the individual, placing significant strain on the capacity of healthcare systems and underscoring the urgent need for effective interventions (Frisoni et al., 2023). This study provides a comprehensive analysis of the global trends and disparities in the burden of ADOD among adults aged 65 years and older from 1990 to 2021, with forward-looking projections into 2050, to inform clinical and public health strategies.

Globally, dementia affects over 55 million individuals, with projections indicating an annual increment of nearly 10 million new cases (World Health Organization [WHO], 2021). The clinical relevance of ADOD is underscored by its escalating prevalence and the profound impact on the quality of life for affected individuals and their caregivers (Kalaria et al., 2024). Epidemiological data indicate a concerning increase in the incidence and prevalence of ADOD, with significant heterogeneity observed across different geographical regions and sociodemographic groups (GBD 2021 Causes of Death Collaborators, 2024). This comprehensive analysis will focus on specific aspects of ADOD, 60 including age-standardized prevalence, mortality rates, and DALYs. In addition, the role of risk factors and the impact of the sociodemographic index (SDI) on these trends will be assessed (GBD 2021 Causes of Death Collaborators, 2024). By synthesizing data from 1990 to 2021 and projecting future scenarios to 2050, our objective is to offer a nuanced understanding of ADOD in the context of global ageing. While previous studies have documented the global burden of dementia, this study provides an update using the most recent GBD 2021 data, with a specific focus on the population aged 65 and above. It further incorporates a novel analysis of cross-country health inequalities and provides robust projections to 2050, aiming to identify new trends and disparities that can inform targeted public health strategies.

## 2 Materials and methods

### 2.1 Study population and data collection

Using data from from 100,983 sources, the GBD 2021 database detailed 371 diseases and injuries, along with 88 risk factors and their health impacts. This study included patients aged 65 years or older with ADOD, as defined according to World Health Organization (WHO) (World Health Organization [WHO], 2018). We estimated the global trends in the ADOD burden through age-standardized metrics encompassing prevalence, mortality, and DALYs rates. Additionally, we analyzed DALYs attributable to risk factors. All these metrics were stratified by age (every 5 years from 65–69 to 95+ years of age), gender, and region from 1990 to 2021 (“Methods” section of the Supplementary Appendix) (GBD 2021 Diseases and Injuries Collaborators, 2024; Pérez et al., 2024).

### 2.2 Fatal and nonfatal burdens modelling

The GBD 2021 study employed a comprehensive methodology to model deaths and estimate the nonfatal burdens of ADOD. We used literature, vital registration, and hospitalization data to model mortality, while prevalence, age-standardized prevalence, and age-standardized DALY rates were generated using the DisMod-MR 2.1 modelling tool, which integrates factors such as gender, region, time, and age groups (GBD 2021 Demographics Collaborators, 2024; GBD 2021 Diseases and Injuries Collaborators, 2024). This systematic approach involved using relative risk data and a Bayesian meta-regression model to calculate attributable risks and all-cause mortality rates. The study further refined these estimates by integrating clinical and mortality data to identify severe, end-stage dementia cases, adjusting global mortality figures, and projecting estimates from 1980 to 2022 using log-linear interpolation (section 4–5 of the Supplementary Appendix).

### 2.3 Risk factor assessment

To quantify the impact of specific risk factors on the total burden of disease, we used the measure of attributable DALYs. This measure captures the potential reduction in disease burden if exposure to a given risk factor were to change at the population level. After accounting for comorbidity, we used a microsimulation approach to arrive at a refined estimate of years lived with disability. Years of life lost were calculated by multiplying the estimated number of deaths due to ADOD by the standard life expectancy at the age of death. The total number of DALYs was then derived by summing years lived with disability and years of life lost. The methodology used in our analysis is consistent with that described in the GBD 2021, with additional details provided in the Supplementary methods section (GBD 2021 Causes of Death Collaborators, 2024). Notably, due to data limitations, the impact of the coronavirus disease 2019 (COVID-19) pandemic on risk factors or health outcomes was not formally included or quantified in this analysis.

### 2.4 Health inequality analyses and predictive analysis

To quantify absolute and relative cross-country inequalities in ADOD burden, we employed two complementary indices recommended by the World Health Organization in 2023 (World Heath Organization [WHO], 2023). The Slope Index of Inequality (SII) measures the absolute difference in age-standardized rates between the most- and least-advantaged countries based on SDI rankings. Calculated via least-squares regression weighted by population size using country-level data, where SII >0 indicates a higher burden in the most-advantaged (high-SDI) countries. The Concentration Index (CI) assesses relative inequality by measuring how the ADOD burden is distributed across the SDI spectrum. Defined as twice the area between the concentration curve and the line of equality (range: −1 to 1). CI >0 implies the burden is concentrated among higher-SDI populations. These indices were selected over simpler metrics to account for the full socioeconomic gradient (SDI continuum), not just extreme groups (section 7 of the Supplementary Appendix). The association between national SDI values and age-standardized ADOD prevalence was evaluated using Spearman’s rank correlation—a nonparametric method chosen for its robustness to nonlinear relationships and outliers. This analysis aims to test the hypothesis that population-level development (SDI) correlates with dementia burden. No frontier analysis was performed, as the primary goal was descriptive correlation testing. To forecast the number and incidence of new ADOD cases from 2022 through 2050, Bayesian age-period-cohort (BAPC) models were employed (section 8 of the Supplementary Appendix) (Riebler and Held, 2017). The BAPC models were fitted using integrated nested Laplace approximations (INLA) with weakly informative priors. The models incorporated 5-year age, period, and cohort intervals. Projection uncertainty was quantified and presented as 95% uncertainty intervals (UIs), which represent credible intervals from the posterior distribution.

### 2.5 Data source and presentation

For the GBD 2021 study, we sourced data from 22,824 vital registration sources, 513 nonfatal health outcome sources, 18 relative risk sources, and 11,538 exposure and TMREL sources. For more detailed information about the data sources used in our analysis, please refer to the GBD 2021 source tools^1^. The estimates were presented in both absolute numbers and age-standardized rates per 100,000 population, accompanied by a 95% uncertainty interval (UI). UIs represent the 2.5th and 97.5th percentiles of the posterior distribution generated by Bayesian models, accounting for input data variability, model parameter uncertainty, and demographic stochasticity. The data was categorized by age, sex, 21 GBD regions, 7 GBD super regions, and 5 SDI levels. The SDI for each country was calculated using the most recent 2021 data (section 10 of the Supplementary Appendix). The SDI is a comprehensive indicator that captures the social and demographic development of a region and is scored on a scale from 0 (lowest) to 1 (highest), with 1 indicating the highest education level, the highest per capita income, and the lowest fertility rate.

### 2.6 Statistical analysis

We conducted a thorough descriptive analysis to delineate the global burden of ADOD in adults aged 65 years and older. We conducted a comparative analysis of age-standardized prevalence, mortality, and DALYs across various dimensions, including age, sex, region, and country groups. The age-standardized rates for each region were calculated with data from the GBD study and aligned with the world standard population as referenced in the GBD 2021 report. UIs were generated through probabilistic modeling to quantify variability across all stages of estimation. The 95% UIs, reflecting the 2.5th and 97.5th percentiles of 1,000 Monte Carlo simulations, are reported to facilitate cross-regional comparisons. Furthermore, we estimated the average annual percentage change (AAPC) for each year, employing a join-point regression model (Joinpoint Regression Program, version 5.2.0.0) to track the progression of the disease burden over time (Yang K. et al., 2024). The joinpoint analysis was used to test whether significant changes in trends occurred during the 30-year period. However, no significant joinpoints were identified for the primary global trends, indicating relatively stable progression patterns. Therefore, the AAPC provides a valid summary measure of the constant annual percentage change across the entire study period. All the results are expressed per 100,000 population, and the specific calculation formula is as follows.


$$A\,g\,e-s\,t\,a\,n\,d\,a\,r\,d\,i\,z\,e\,d\,r\,a\,t\,e=\frac{\sum_{i=1}^{A}a_{i}\,w_{i}}{\sum_{i=1}^{A}w_{i}}$$

*Where:*

a_*i*_ is the age specific rate and w_*i*_ is the weight in the same age subgroup of the chosen reference standard population (in which i denotes the i*^th^* age class) and A is the upper age limit.

The AAPC is a statistical measure that captures the overall trend of change in a variable across a defined time span (Kim et al., 2000). In this study, the AAPC was calculated with slope coefficients from a base join-point regression model, which spans from 1990 to 2021. These coefficients were then converted into an annual percentage change. Significance testing was conducted via a Monte Carlo permutation method, which can account for the variability estimated at each data point, or a Poisson model was employed to assess variation. The AAPC indicates whether a variable is increasing, decreasing, or remains stable on an annual basis. If the estimate of the annual percentage change and its 95% CI are consistently greater than zero, it signifies a significant upward trend in the variable. Conversely, if these values are consistently less than zero, a significant downward trend is indicated. The AAPC was calculated via the following formula:


$$A\,A\,P\,C=\left\{e\,x\,p\,\left(\frac{\sum w_{i}\,b_{i}}{\sum w_{i}}\right)-1\right\}\times 100$$

*Where:*

b_*i*_ is the slope coefficient for the i*^th^* segment with i indexing the segments in the desired range of years, and w_*i*_ is the length of each segment in the range of years.

All statistical analyses were conducted using GraphPad Prism (version 9.5.0), Joinpoint Regression Program (version 5.2.0.0), R (version 4.4.1), and Stata (version MP 17).

## 3 Results

### 3.1 Global trends

**FIGURE 1:**
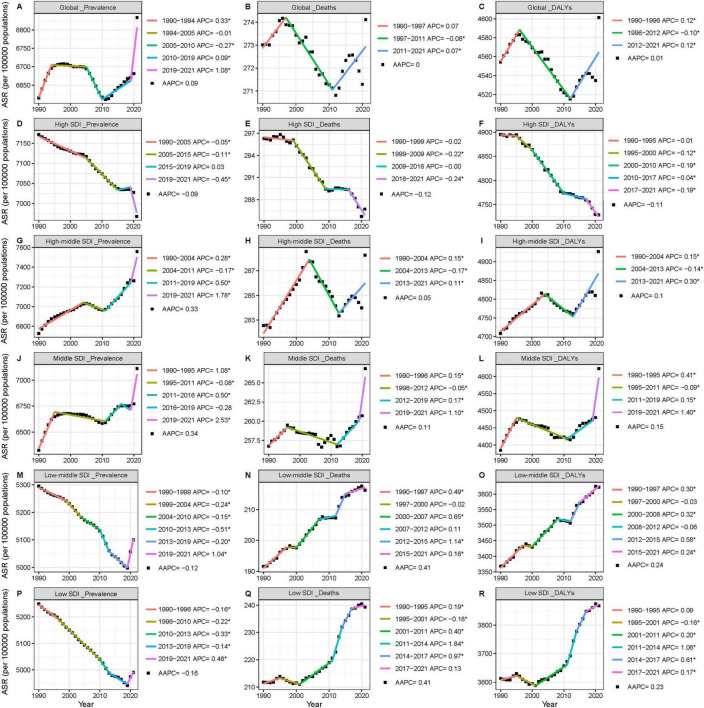
Trends in age-standardized rates for different regions of ADOD among people aged ≥65 years based on joinpoint regression model, 1990–2021**. (A–C)** Global trends. **(D–F)** High-SDI region. **(G–I)** High-middle-SDI region. **(J–L)** Middle-SDI region. **(M–O)** Low-middle-SDI region. **(P–R)** Low-SDI region. For each row, panels show: **(A,D,G,J,M,P)** Age-standardized prevalence rates (per 100,000 population). **(B,E,H,K,N,Q)** Age-standardized mortality rates (per 100,000 population). **(C,F,I,L,O,R)** Age-standardized DALY rates (per 100,000 population). Data source: Global Burden of Disease Study 2021. ADOD, Alzheimer’s disease and other dementias; SDI, socio-demographic index; DALYs, disability-adjusted life years; ASR, age-standardized rate. The asterisk symbol (*) denotes statistical significance and corresponds to *p* < 0.05.

From 1990 to 2021, the number of adults aged 65 years or older living with ADOD increased by 171%, from 18.1 million (95% UI 14.4–22.5) to 49.1 million (38.7–61.3). During this period, the age-standardized prevalence of ADOD among this age group showed a slight increase of 3%, from 6,616 (95% UI 5,271–8,205) to 6,833 (5,390–8,522) cases per 100,000 population, with an average annual trend of 0.09% (Figure 1 and Table 1). Furthermore, the proportion of ADOD patients in this age group steadily increased, from 83.1% in 1990 to 86.4% in 2021 (Supplementary Figure 1). Both the overall population prevalence of ADOD and the prevalence within this specific age group demonstrated a slow upward trend from 1990 to 2021 (Supplementary Figure 2).

**TABLE 1:** Age-standardized prevalence and AAPC of ADOD in people aged ≥65 years at global and regional level, 1990–2021.

	Prevalence (95% UI)					
Subgroup	No of people with ADOD in 1990 (00000s)	Age-standardized rate in 1990 (per 100 000)	No of people with ADOD in 2021 (00000s)	Age-standardized rate in 2021 (per 100 000)	AAPC (95% CI)	*P*-value
Global	181 (144 to 225)	6616 (5271 to 8205)	491 (387 to 613)	6833 (5390 to 8522)	0.09 (0.08 to 0.1)	<0.001
**Sex:**						
Female	121 (97 to 150)	7243 (5786 to 8963)	318 (252 to 396)	7603 (6023 to 9469)	0.14 (0.12 to 0.16)	<0.001
Male	60 (47 to 75)	5575 (4398 to 6960)	173 (135 to 217)	5744 (4486 to 7205)	0.08 (0.05 to 0.12)	<0.001
**Age group (years):**						
65–69	22 (17 to 28)	1808 (1414 to 2275)	54 (42 to 67)	1952 (1518 to 2437)	0.23 (0.22 to 0.25)	<0.001
70–74	28 (22 to 36)	3349 (2622 to 4255)	74 (57 to 94)	3578 (2770 to 4565)	0.2 (0.14 to 0.25)	<0.001
75–79	41 (33 to 51)	6707 (5377 to 8230)	93 (74 to 115)	7071 (5629 to 8702)	0.15 (0.13 to 0.17)	<0.001
80–84	44 (36 to 55)	12552 (10140 to 15548)	114 (91 to 142)	13002 (10368 to 16185)	0.1 (0.09 to 0.11)	<0.001
85–89	30 (24 to 37)	19790 (15790 to 24310)	92 (73 to 113)	20032 (15865 to 24816)	0.03 (0.01 to 0.05)	0.011
90–94	12 (9 to 14)	26857 (21377 to 33165)	47 (37 to 59)	26408 (20745 to 33046)	−0.07 (−0.09 to −0.04)	<0.001
≥ 95	3 (3 to 4)	33789 (26540 to 42083)	18 (14 to 22)	32194 (24954 to 40786)	−0.16 (−0.18 to −0.14)	<0.001
**SDI level:**						
High	72 (58 to 88)	7172 (5780 to 8805)	157 (125 to 194)	6968 (5546 to 8619)	−0.09 (−0.1 to −0.08)	<0.001
High-middle	47 (37 to 59)	6727 (5325 to 8370)	130 (102 to 163)	7559 (5922 to 9472)	0.33 (0.27 to 0.39)	<0.001
Middle	37 (29 to 47)	6324 (4957 to 7935)	141 (110 to 177)	7113 (5575 to 8922)	0.34 (0.25 to 0.44)	<0.001
Low-middle	18 (14 to 23)	5295 (4162 to 6636)	48 (38 to 61)	5100 (4004 to 6411)	−0.12 (−0.13 to −0.1)	<0.001
Low	6 (5 to 8)	5250 (4105 to 6578)	14 (11 to 18)	4991 (3924 to 6248)	−0.16 (−0.18 to −0.14)	<0.001

AAPC, average annual percentage change; CI, confidence interval; SDI, sociodemographic index; ADOD, Alzheimer’s disease and other dementias; UI, uncertainty interval. *P*-value for the significant test of AAPCs.

Notably, the age-standardized mortality rate for ADOD in this age group remained relatively stable, with a slight fluctuation from 273 (95% UI 66–738) cases per 100,000 population in 1990 to 274 (69–722) cases per 100,000 population in 2021, indicating an average annual trend of 0%. The number of ADOD-related DALYs in this age group increased by 176% over the same period. However, when standardized by age, the number of DALYs showed a 1% increase, with an annual trend of 0.02% (Figure 1 and Supplementary Tables 1, 2).

### 3.2 Global trends by sex

From 1990 to 2021, the global prevalence of ADOD among adults aged 65 years and older increased for both females (12.1 [95% UI 9.7–15.0] to 31.8 [25.2–39.6] million) and males (6.0 [95% UI 4.7–7.5] to 17.3 [13.5–21.7] million). When standardized by age, the prevalence showed a more pronounced increase among females, increasing from 7,243 (95% UI 5,786–8,963) to 7,603 (6,023–9,469) cases per 100,000 population, compared with the increase observed among males, which increased from 5,575 (4,398–6,960) to 5,744 (4,486–7,205) cases per 100,000 population (AAPC of 0.14% for females vs. 0.08% for males) (Table 1). Notably, the prevalence increase in females consistently exceeded that in males across all age subgroups over 70 years, with a particularly marked difference observed in females aged 70–74 years (AAPC 0.25%, Supplementary Figure 3).

During the same period, the age-standardized mortality rate for ADOD was consistently higher in females than in males, with females exhibiting 301 (95% UI 74–802) to 304 (78–782) cases per 100,000 population, and males showing a lower range from 219 (51–615) to 225 (54–626) cases per 100,000 population (AAPC of 0.02% for females vs. 0.08% for males, Supplementary Table 1). In terms of age-standardized DALYs, females also bore a greater burden, increasing by 2% (from 5,057 [95% UI 2,289–11,107] to 5,158 [2,378–10,999] cases per 100,000 population), while males saw a 3% increase (from 3,675 [1,635–8,516] to 3,770 [1,692–8,643] cases per 100,000 population, Supplementary Table 2).

This disparity between the sexes was observed across all SDI levels and age subgroups, with females generally having a greater burden of ADOD than males did, especially in countries with high-middle SDIs (Supplementary Figures 4,5).

### 3.3 Global trends by age subgroups

From 1990 to 2021, a significant global increase in the number of older adults with ADOD was observed across all age subgroups, with the 95+ sub-group experiencing a sixfold increase. All these age subgroups experienced an astonishing increase of over 200% in the number of ADOD cases. At the same time, age-standardized prevalence rates have increased for all age subgroups except those aged over 90 (Table 1 and Supplementary Figure 3).

In 2021, the age-standardized mortality rate for ADOD increased with age, starting at 31 (95% UI 7–92) cases per 100,000 for individuals aged 65–69 years and ending at 3,906 (987–9,744) cases per 100,000 for those aged over 95 years. However, from 1990 to 2021, the age-standardized mortality rates of ADOD across all age groups have remained relatively stable (Supplementary Table 1).

The number of age-standardized DALYs for ADOD also increased with age. Between 1990 and 2021, within the 65–79 age subgroup, there was a marked increase in the number of age-standardized DALYs. In 2021, the highest number of age-standardized DALYs was observed in individuals aged 95 years and older, reaching 38,958 (95%UI 15,644–90,038) cases per 100,000 population (Supplementary Table 2).

### 3.4 Global trends by sociodemographic index

From 1990 to 2021, the age-standardized prevalence for ADOD among adults decreased in most SDI subgroups, except for the high-middle and middle-SDI group which showed an increase (AAPC 0.33% and 0.34%). The impact of ADOD on the age-standardized prevalence became more pronounced as the SDI exceeded the threshold of 0.63 (Supplementary Figure 6). In 2021, the highest age-standardized prevalence rates were observed in high-middle-SDI countries, at 7,559 (95% UI 5,922–9,472) per 100,000 population (Table 1). Regardless of sociodemographic level, the increase in prevalence was consistently higher in those aged ≥65 years than in the overall population (Supplementary Figures 2, 7).

From 1990 to 2021, the age-standardized mortality rates for ADOD increased across all subgroups except for the high-SDI subgroup. In 2021, high-middle-SDI countries had the highest age-standardized mortality rate, at 288 (95% UI 71–776) per 100,000 population (Supplementary Table 1 and Supplementary Figures 4, 5).

The age-standardized DALYs for ADOD among adults aged 65 years and older clearly increased across all subgroups except for the high-SDI subgroup, with the most significant increase in low-middle-SDI countries (APCC 0.24%). In 2021, high-middle-SDI countries had the highest number of age-standardized DALYs at 4,927 (95% UI 2,266–10,775) years per 100,000 population (Supplementary Table 2).

### 3.5 Regional trends

From 1990 to 2021, the age-standardized prevalence of ADOD increased only in East Asia and High-income Asia Pacific (AAPC 0.79% and 0.16%), remained steady in Central Sub-Saharan Africa and Tropical Latin America (AAPC 0.00%), and decreased in most of the 21 regions. The most reduced burden was estimated in Australasia (AAPC −0.54%). In 2021, the highest rates were in East Asia (8,792 [95% UI 6,862–11,019] per 100,000), North Africa and Middle East (7,677 [6,021–9,569] per 100,000), and High-income North America (7,455 [5,879–9,272] per 100,000, Supplementary Table 3 and Supplementary Figure 8). When the analysis was stratified by sex, no significant differences were observed across different regions (Supplementary Table 4).

One third of the regions showed an increase in age-standardized mortality rates for ADOD from 1990 to 2021. The most significant increases were observed in South Asia (AAPC 0.69%), Central Sub-Saharan Africa (0.46%), and Australasia (0.40%). In contrast, the most significant decreases were observed in North Africa and Middle East (AAPC −0.28%), Oceania (−0.28%), and Australasia (−0.19%, Supplementary Figure 8 and Supplementary Table 5).

Approximately 50% of the regions experienced an increase in the number of age-standardized DALYs in old people from 1990 to 2021, with South Asia experiencing the highest increase (AAPC 0.43%). The greatest reduction in age-standardized DALYs was estimated in Australasia (AAPC −0.31%). In 2021, the highest numbers of DALYs were noted in Central Sub-Saharan Africa (6,062 [95% UI 2,518 –14,313] per 100,000), East Asia (5,685 [2,619–12,421] per 100,000), and High-income North America (5,076 [2,321–10,911] per 100,000). The lowest numbers of DALYs were observed in Andean Latin America (2,706 [95% UI 1,261–5,839] per 100,000, Supplementary Figure 8 and Supplementary Table 6). When the analysis was stratified by sex, no significant differences were found across different regions (Supplementary Figure 9).

### 3.6 National trends

**FIGURE 2:**
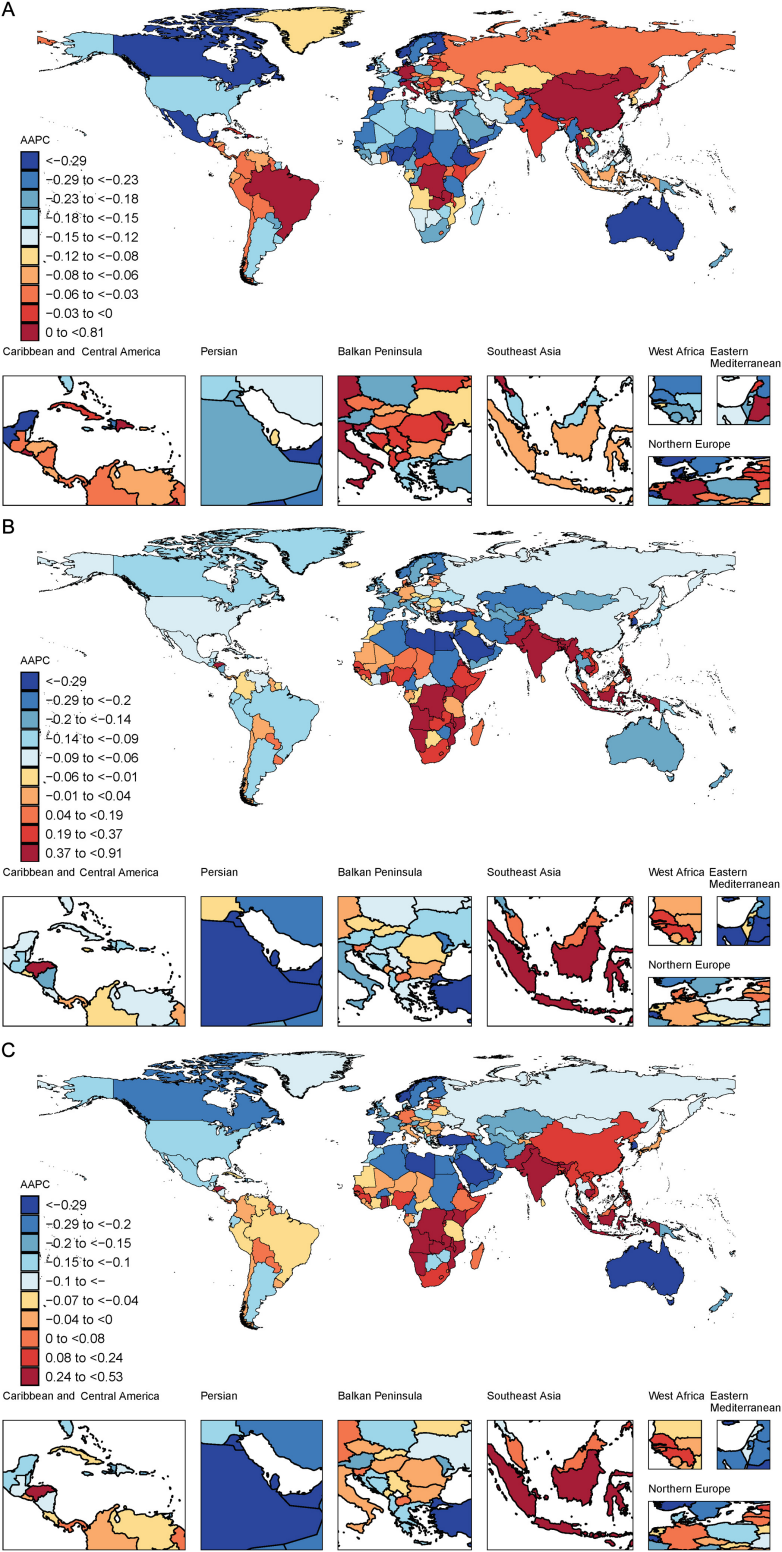
Maps showing average annual percentage change (AAPC) in global age-standardized rates of ADOD among people aged ≥65 years, 1990–2021. **(A)** Map showing average annual percentage change in global prevalence of ADOD among people aged ≥65 years, 1990–2021; **(B)** Map showing average annual percentage change in global mortality among people with ADOD aged ≥65 years, 1990–2021; **(C)** Map showing average annual percentage change in global DALYs among people with ADOD aged ≥65 years, 1990–2021. Data source: Global Burden of Disease Study 2021. ADOD, Alzheimer’s disease and other dementias; DALYs, disability-adjusted life years.

From 1990 to 2021, the greatest increase in the age-standardized prevalence of ADOD was observed in China, with an average annual trend of 0.81%, followed by Italy (AAPC 0.39%) and Japan (AAPC 0.16%). Conversely, the most significant decrease in the age-standardized prevalence was observed in Denmark, with an average annual trend of −0.73%, followed by Norway (AAPC −0.62%), Australia (AAPC -0.60%). In 2021, the highest age-standardized prevalence was observed in China, at 8,921 (95% UI 6955–11195) cases per 100,000 population, whereas the lowest was observed in Nigeria, at 3,567 (2,763–4,538) cases per 100,000 population (Figure 2 and Supplementary Table 7).

The age-standardized mortality rate for ADOD decreased in most countries among this age group, with the greatest decrease in Guam, with an AAPC of −0.81, followed by Republic of Korea, with a value of −0.69, and United Arab Emirates, with a value of −0.58. In 2021, Gabon presented an age-standardized mortality rate of 387 (95% UI 91–1,043) cases per 100,000 population, whereas Peru presented the lowest rate of 150 (35–399) cases per 100,000 population (Figure 2 and Supplementary Table 8).

From 1990 to 2021, the most significant increase in the number of age-standardized DALYs for ADOD was observed in India (AAPC 0.53%), followed by Indonesia (AAPC 0.52%) and Uganda (AAPC 0.46%). The most significant decreases in the number of age-standardized DALYs were observed in United Arab Emirates (AAPC −0.57), Republic of Korea (AAPC −0.52), and Guam (AAPC −0.48). In 2021, Democratic Republic of the Congo presented the highest number of age-standardized DALYs for old people, at 6,152 (95% UI 2,545–14,397) years per 100,000 population, whereas Peru presented the lowest, at 2,669 (1,243–5,732) years per 100,000 population (Figure 2 and Supplementary Table 9).

### 3.7 Risk factors

A detailed analysis of global data from 1990 to 2021 revealed three primary risk factors associated with DALYs for ADOD among people aged ≥65 years, including high fasting plasma glucose, high body-mass index, and smoking. In 2021, these factors accounted for 694 (95% UI 40–1,903), 328 (−58 to 1,214), and 168 (70–386) age-standardized DALYs per 100,000 population, respectively. From 1990 to 2021, the corresponding AAPCs for these factors were 1.12%, 1.30%, and −0.78%. In all SDI subgroup countries, the most substantial increased burden from 1990 to 2021 was associated with a high fasting plasma glucose level (AAPC 1.12%) and high body-mass index (1.30%). In contrast, countries with a high SDI contributed the most to reducing the burden attributed to smoking (Table 2).

**TABLE 2:** Main risk factors for age-standardized ADOD related DALYs, and AAPC, among people aged ≥65 years, 1990–2021.

	Age-standardized DALYs (per 100 000) (95% UI)			
Risk factors by SDI	1990	2021	AAPC (95% CI)	*P*-Value
**High fasting plasma glucose**				
Global	492 (29 to 1343)	694 (40 to 1903)	1.12 (1.08 to 1.16)	<0.001
High SDI	536 (32 to 1440)	787 (47 to 2139)	1.26 (1.2 to 1.32)	<0.001
High-middle SDI	482 (28 to 1328)	671 (40 to 1816)	1.09 (1.05 to 1.14)	<0.001
Middle SDI	503 (27 to 1409)	657 (37 to 1815)	0.87 (0.84 to 0.91)	<0.001
Low-middle SDI	373 (21 to 1019)	594 (32 to 1684)	1.52 (1.43 to 1.6)	<0.001
Low SDI	359 (19 to 1006)	517 (26 to 1505)	1.19 (1.11 to 1.26)	<0.001
**High body-mass index**				
Global	218 (−14 to 860)	328 (−58 to 1214)	1.3 (1.28 to 1.32)	<0.001
High SDI	325 (−44 to 1221)	424 (−89 to 1505)	0.86 (0.84 to 0.89)	<0.001
High-middle SDI	261 (−26 to 1039)	402 (−79 to 1452)	1.38 (1.33 to 1.42)	<0.001
Middle SDI	80 (−1 to 386)	252 (−35 to 984)	3.74 (3.69 to 3.79)	<0.001
Low-middle SDI	62 (−1 to 292)	167 (−21 to 660)	3.26 (3.18 to 3.34)	<0.001
Low SDI	23 (−12 to 160)	90 (−4 to 428)	4.45 (4.41 to 4.49)	<0.001
**Smoking**				
Global	213 (87 to 491)	168 (70 to 386)	−0.78 (−0.81 to −0.75)	<0.001
High SDI	253 (107 to 577)	171 (72 to 385)	−1.26 (−1.29 to −1.23)	<0.001
High-middle SDI	200 (80 to 464)	196 (81 to 451)	−0.09 (−0.17 to −0.01)	0.025
Middle SDI	219 (88 to 505)	178 (73 to 419)	−0.69 (−0.76 to −0.62)	<0.001
Low-middle SDI	149 (61 to 357)	121 (48 to 285)	−0.65 (−0.69 to −0.61)	<0.001
Low SDI	91 (37 to 215)	76 (29 to 184)	−0.58 (−0.67 to −0.48)	<0.001

AAPC, average annual percentage change; CI, confidence interval; DALYs, disability adjusted life years; SDI, sociodemographic index; ADOD, Alzheimer’s disease and other dementias; UI, uncertainty interval. *P*-value for the significant test of AAPCs.

### 3.8 Health inequality analyses

**FIGURE 3:**
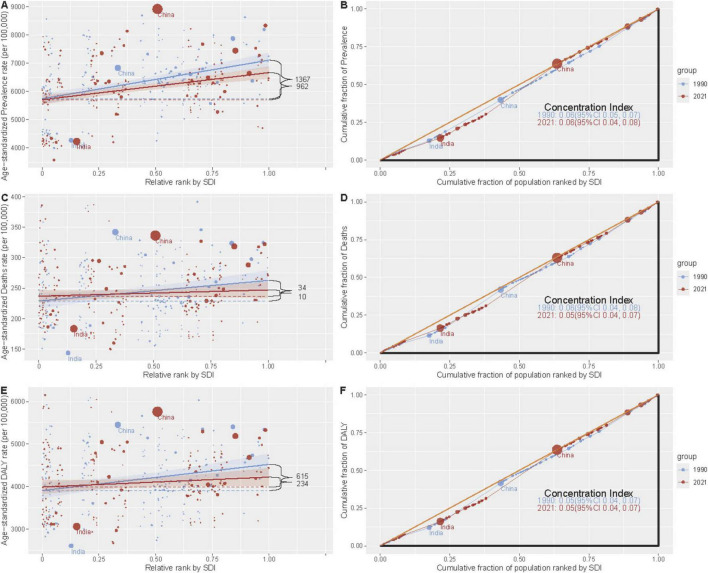
Health inequality regression curves and concentration curves for the age-standardized prevalence, mortality, and DALYs of ADOD among people aged ≥65 years from 1990 to 2021 across the world. Panels show: **(A,B)** Prevalence. **(C,D)** Mortality. **(E,F)** DALYs. **(A,C,E)** Slope Index of Inequality (SII) regression curves. The SII (red line) represents the absolute difference in burden between the most- and least-advantaged countries. A positive SII indicates a higher burden in higher-SDI countries. **(B,D,F)** Concentration curves. The Concentration Index (CI) measures relative inequality. A curve below the line of equality (diagonal dashed line) indicates a concentration of the burden among higher-SDI populations. Data source: Global Burden of Disease Study 2021. ADOD, Alzheimer’s disease and other dementias; SDI, socio-demographic index; DALYs, disability-adjusted life years.

The slope index of inequality demonstrated that in the age-standardized prevalence, mortality, and DALY rates of ADOD among countries with the highest and lowest SDI values, there was a decline from 1367 (95% UI: 853–1,881), 34 (9–58), and 615 (240–990) in 1990 to 962 (547–1,377), 10 (−11 to 31), and 234 (−72 to 539) in 2021, respectively. This decline indicates a reduction in the absolute disparity between high- and low-SDI countries. In contrast, the relative gradient inequality, as measured by the concentration index, was 0.06, 0.06, and 0.05 in 1990 and 0.06, 0.05, and 0.05 in 2021, respectively (Figure 3 and Supplementary Table 10). These findings suggest that relative inequalities in ADOD burden have not widened, despite the persistence of absolute disparities. This finding underscores the predominant influence of demographic aging on global ADOD patterns, eclipsing the impact of socioeconomic disparities.

**FIGURE 4:**
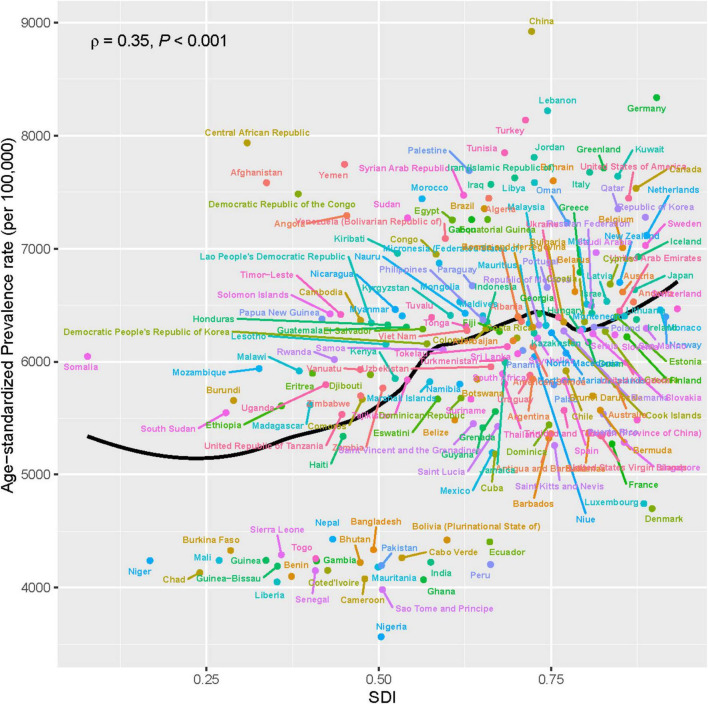
Association between age-standardized prevalence of ADOD among people aged ≥65 years and SDI. Data source: Global Burden of Disease Study 2021. ADOD, Alzheimer’s disease and other dementias; SDI, socio-demographic index.

To explore the relationship between SDI and the age-standardized prevalence of ADOD, Spearman correlation analyses were performed (Figure 4). The age-standardized prevalence of ADOD was positively correlated with SDI at the national and regional levels (*r* = 0.35, *P* < 0.001), meaning that the higher the SDI in a country or region, the higher the prevalence of ADOD. However, there was no correlation between age-standardized mortality and DALY rates of ADOD and SDI (*P* = 0.472, 0.234, Supplementary Figures 10, 11).

### 3.9 Global trends of ADOD predicted by the BAPC model

The BAPC model projected that the age-standardized prevalence of ADOD among this population will increase significantly worldwide, from approximately 6,833 (95% UI 6,824–6,843) cases per 100,000 population in 2021 to approximately 10,254 (2,684–17,823) cases per 100,000 population in 2050, with 191 (52–330) million cases. This represents an increase of 50.1% over three decades.

**FIGURE 5:**
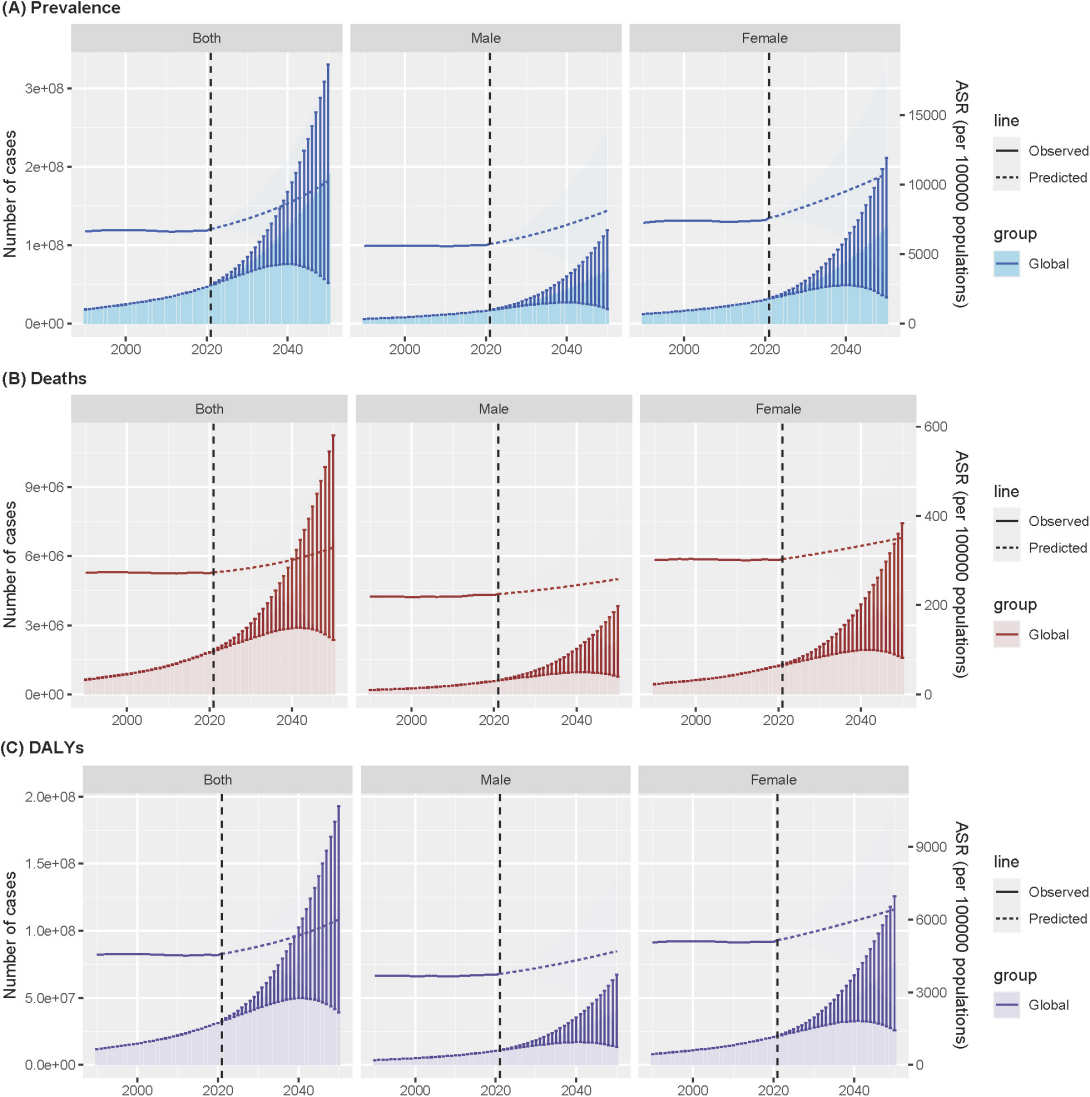
Age-standardized ADOD among people aged ≥65 years prevalence **(A)**, mortality **(B)**, and DALYs **(C)** over time at global, with projections up to 2050. Data source: Global Burden of Disease Study 2021. ADOD, Alzheimer’s disease and other dementias; DALYs, disability-adjusted life years; ASR, age-standardized rate.

Globally, the age-standardized mortality rate is projected to increase to approximately 330 (112–547) cases per 100,000 population, resulting in an estimated 6.8 (2.4–11.3) million deaths by 2050. Similarly, the age-standardized DALY rate will increase to 6,004 (1,965–10,043) cases per 100,000 population, with 116 (39–193) million cases of DALYs projected in 2050 (Figure 5 and Supplementary Tables 11–13). Notably, sex differences persist, with females having a greater burden of disease than males in all three indicators. Moreover, the difference in prevalence between males and females appears to be widening, a trend that has become evident over time.

## 4 Discussion

The key findings of our study highlight a significant increase in the global burden of ADOD among adults aged 65 years and older from 1990 to 2021. Specifically, the number of prevalent ADOD cases surged by 171%, culminating in nearly 50 million cases in 2021, with projections indicating this number will soar to 191 million by 2050. While the age-standardized prevalence showed a slight increase of 3%, the age-standardized mortality rate remained relatively stable. This finding contrasts with the notable 176% increase in the number of ADOD-related DALYs, underscoring the significant impact of the disease on quality of life and functional status. These findings underscore the urgent need for effective interventions to address the growing challenge posed by ADOD.

Several key findings merit further discussion. First, the global increase in the prevalence of ADOD is consistent with the findings of previous studies, which have also reported a significant rise in the number of dementia cases worldwide (GBD 2021 Nervous System Disorders Collaborators, 2024; Livingston et al., 2020). The WHO has estimated that the global population of individuals aged 60 and above will reach 2.1 billion by 2050 (World Health Organization [WHO], 2024). The observed increase in ADOD burden can be partially attributed to the aging global population, as the proportion of individuals aged 65 and older continues to rise. This demographic shift is particularly pronounced in countries with high-middle- and middle-SDI, where the age-standardized prevalence of ADOD has increased significantly. Conversely, countries with high-SDI exhibited a declining trend in the burden of ADOD, indicating that socioeconomic factors may exert a pivotal influence on disease incidence and progression.

Gender-specific analyses revealed a consistently higher age-standardized prevalence and mortality rate among females compared to males across all SDI levels and age subgroups. This finding aligns with extensive evidence highlighting the greater vulnerability of females to dementia (Gong et al., 2023; Jia et al., 2020; Martinkova et al., 2021). The observed 32% higher age-standardized prevalence in females (Table 1) likely stems from synergistic biological and social pathways, including longer post-diagnosis survival and sex-specific risk trajectories such as menopause-related metabolic dysregulation (Cui et al., 2023; Saif et al., 2022). While genetic and sociocultural factors also contribute, this multifactorial etiology necessitates integrating gender-sensitive cognitive assessments into routine geriatric care to enable early detection. Further investigation should prioritize mechanistic studies targeting female-specific risk profiles to optimize interventions.

It is also notable that there are considerable regional variations in the ADOD burden. In 2021, the East Asia and High-income Asia Pacific regions exhibited the highest age-standardized prevalence rates, while the Australasia region demonstrated the most significant decrease in prevalence over the study period. These regions may face unique challenges related to genetic predisposition, rapid urbanization, environmental factors, and lifestyle changes that contribute to the risk of developing ADOD. For example, research has indicated that specific genetic variations may be associated with a higher prevalence of dementia in East Asian populations (Belloy et al., 2023; Duan et al., 2023). Conversely, enhancements in healthcare infrastructure and accessibility in Australasia may have contributed to the observed decline in prevalence, which have facilitated more effective prevention strategies and earlier intervention (Mateo-Arriero et al., 2023; Ranson et al., 2021). Despite declining absolute inequality (SII), the 2.1-fold higher prevalence in high-SDI countries (Figure 3) underscores demographic destiny versus healthcare capacity paradox: advanced economies face ageing-driven caseloads, while low-SDI regions lack diagnostic infrastructure. This dual challenge necessitates tailored interventions stratified by SDI trajectories—scaling telemedicine in resource-poor areas while optimizing long-term care financing in ageing societies. Such stratification aligns with the stable relative inequality, confirming that tailored approaches must address demographic destiny versus infrastructure capacity disparities.

The observed decline in absolute inequality (SII) over time, despite a persistently higher burden in high-SDI countries, presents a complex picture. This phenomenon may be attributed to a “diagnostic and survival paradox.” High-SDI countries, with their advanced healthcare systems, possess greater diagnostic capacity and longer life expectancy, leading to higher recorded prevalence and longer survival with the disease. Concurrently, low- and middle-SDI regions may experience significant under-diagnosis due to limited access to healthcare and stigma (Stangl et al., 2019), alongside higher competing risks of mortality from other causes. The narrowing absolute gap could thus reflect the rapid pace of population aging in middle-SDI countries, which is increasing their ADOD caseload and “catching up” in terms of absolute numbers (GBD 2023 Disease and Injury and Risk Factor Collaborators, 2025), even as relative disparities in diagnosis and care persist.

The role of risk factors in the etiology of ADOD is another important aspect of our study. The identification of high fasting plasma glucose, high body mass index and smoking as primary risk factors for ADOD is consistent with existing evidence linking these factors to cognitive decline and dementia (Biessels and Whitmer, 2020; Nianogo et al., 2022; Qureshi et al., 2024). The observed increase in the burden attributed to high fasting plasma glucose and high body mass index highlights the importance of addressing metabolic disorders and obesity in the prevention of dementia. The 1.52% annual rise in fasting plasma glucose-attributable DALYs in low-middle SDI countries (vs. 1.12% globally) reflects a ‘double burden’ of malnutrition and delayed diabetes management in rapidly urbanizing regions. This signals an urgent need for integrated dementia-diabetes screening programs in primary care. Conversely, it is encouraging that high-SDI countries contributed significantly to the reduction in smoking-related DALYs, indicating the effectiveness of targeted public health campaigns in these regions.

The projected increases in the ADOD burden until 2050, as estimated by the BAPC models, underscore the necessity for immediate and sustained action. With an anticipated 50.1% increase in age-standardized prevalence and a projected 6.8 million deaths and 116.0 million DALYs, the global healthcare system will face unprecedented challenges. Our findings underscore the necessity for: (World Health Organization [WHO], 2023) scaling up dementia prevention programs that target modifiable risk factors (e.g., high fasting plasma glucose and high body-mass index) globally, with tailored campaigns for rapidly aging low- and middle-income countries; (Frisoni et al., 2023) investing in healthcare system preparedness, including workforce training in dementia care and the development of accessible diagnostic infrastructure; and (World Health Organization [WHO], 2021) formulating and financing sustainable long-term care policies and social support systems to alleviate the burden on families and societies.

To guide clinical practice and public health policy, our findings emphasize the necessity for a comprehensive approach that integrates prevention, early diagnosis, and effective management of ADOD. Enhancing access to healthcare services, particularly in low- and middle-SDI countries, is crucial. Our findings on the burden in older adults should be viewed within a broader life-course perspective. Evidence suggests a growing burden of early-onset dementia, highlighting the long preclinical phase of the disease and underscoring the critical importance of preventive interventions beginning early in life (Feng et al., 2025). This life-course approach complements our findings and presents a more comprehensive picture of the escalating global challenge posed by dementia.

Moreover, promoting health literacy and awareness about ADOD risk factors and preventive measures among the general population, especially in aging societies, is imperative. Future research should focus on refining risk prediction models, exploring the impact of novel therapeutic interventions, and investigating the long-term effects of COVID-19 on cognitive health. Additionally, addressing the underrepresentation of specific populations in research studies, such as those from lower-SDI countries and racial/ethnic minorities, will enhance the generalizability and relevance of findings (Ho et al., 2024).

Our analysis of trends up to 2021 precedes recent paradigm shifts in the diagnosis and management of Alzheimer’s disease, which are critical for contextualizing our findings and projections. In diagnosis, the integration of artificial intelligence (AI) with neuroimaging and biomarker data is poised to revolutionize early and precise detection (Kale et al., 2024). Therapeutically, the field has witnessed the landmark approval of disease-modifying immunotherapies (e.g., lecanemab, donanemab), which provide the first clear evidence that clearing amyloid-β can slow clinical progression in early-stage patients (Heneka et al., 2024; Jucker and Walker, 2023). Looking ahead, the investigational pipeline is richly diversified, with over 70% of novel agents targeting non-amyloid, non-tau pathways, such as inflammation and synaptic plasticity, heralding a move toward precision medicine (Cummings et al., 2023). Concurrently, innovations in nanomedicine are being leveraged to enhance drug delivery across the blood-brain barrier, thereby improving the bioavailability and efficacy of therapeutic agents (Singh et al., 2024; Yang H. et al., 2024). Beyond pharmacotherapy, non-pharmacological approaches like exercise are recognized for their potential in prevention and mitigating cognitive decline, particularly at the stage of mild cognitive impairment (Brooks et al., 2024). These collective advances underscore an urgent need for global healthcare systems to prepare for the integration of new diagnostic and therapeutic tools. However, this promising future must be tempered by addressing the concomitant challenges of cost, equitable access, and the requisite clinical infrastructure to ensure these innovations do not widen existing health disparities.

Despite the comprehensiveness of our analysis, several limitations should be acknowledged. Firstly, data availability and quality varied across countries and regions, potentially introducing bias and uncertainty into our estimates. Secondly, the impact of the COVID-19 pandemic on ADOD burden and risk factors was not formally quantified due to data limitations, which could influence future trends (Marsters et al., 2024). Additionally, our analysis concentrated on broad risk factors and did not explore the intricate interplay between genetic, environmental, and lifestyle factors that contribute to ADOD development. While our study focused on prevalence and mortality, future research incorporating incidence data from longitudinal cohorts could further elucidate the interplay between risk factor control and dementia onset and disentangle ageing effects from risk factor trajectories.

## 5 Conclusion

In conclusion, our study provides a comprehensive analysis of the global burden of ADOD and its trends over the past three decades. By elucidating regional disparities, gender differences, and the significance of modifiable risk factors, our objective is to facilitate the formulation of targeted strategies that can mitigate the growing impact of this devastating disease. Future research and policy efforts must build upon these findings to ensure a comprehensive and equitable response to the ADOD epidemic.

## Data Availability

The datasets generated during and/or analyzed during the current study are available in the Global Health Data Exchange GBD 2021 repository, https://ghdx.healthdata.org/gbd-2021/sources.

## Footnotes

1
https://ghdx.healthdata.org/gbd-2021/sources
