# Supplementary material for: Trends and cross-country inequalities in the global burden of Alzheimer’s disease and other dementias among adults aged 65+ years, 1990–2021: a population based study with projections into 2050
Source: Front Aging Neurosci. 2026 Jan 29;17:1683158. doi: 10.3389/fnagi.2025.1683158 (PMC12894397; doi:10.3389/fnagi.2025.1683158)
Supplement: Supplementary file 1 [file Data_Sheet_1.pdf]

Supplementary appendix

This appendix formed part of the original submission.

Supplement to: Trends and cross-country inequalities in the global burden of Alzheimer's disease and other dementias among adults aged 65+ years, 1990-2021: a population based study with projections into 2050

Content

Supplementary Methods 4
1. GBD Overview 4
2. Data sources 4
3. Calculation methods for DALYs 5
3.1 YLLs 5
3.2 YLDs 6
4. Estimation of ADOD non-fatal burdens in GBD 2021 7
4.1 Case definition 7
4.2 Data seeking and flowchart 8
4.3 Data inputs and processing 8
4.4 Modelling strategy 8
5. Estimation of ADOD fatal burdens in GBD 2021 9
5.1 Flowchart 9
5.2 Data inputs 10
5.3 Modelling strategy 11
6. Estimation of ADOD burdens attributable to risk factor in GBD 2021 13
6.1 Definitions of risk factors in GBD 2021 14
7. Estimation of ADOD cross-country health inequality in GBD 2021 14
7.1 Slope index of inequality 15
7.2 Concentration index 15
8. Predictive analysis of ADOD 16
9. Data accuracy 17
9.1 Data extractions in each country 17
10. Socio-demographic Index (SDI) 18
10.1 SDI definition 18
10.2 SDI calculation 18
10.3 SDI grouping and quantification 19
11. Reference 55
Additional Results in Tables and Figures 57
Table S1. Age standardised mortality of ADOD in people aged ≥65 years and their AAPC at global and regional level, 1990-2021 57
Table S2. Age standardised DALYs of ADOD in people aged ≥65 years and their AAPC at global and regional level, 1990-2021 58
Table S3. Age standardized prevalence of ADOD in people aged ≥65 years and their AAPCs at regional levels, 1990-2021 59
Table S4. Age standardized prevalence, mortality and DALYs of ADOD in people aged ≥65 years at regional levels by sex, 2021 61
Table S5. Age standardized mortality of ADOD in people aged ≥65 years and their AAPCs at regional levels, 1990-2021 63
Table S6. Age standardized DALYs of ADOD in people aged ≥65 years and their AAPCs at regional levels, 1990-2021 65
Table S7. Age standardized prevalence of ADOD in people aged ≥65 years in 2021 and their AAPCs between 1990-2021 in 204 countries and territories. 67

Table S8. Age standardized mortality of ADOD in people aged ≥65 years in 2021 and their AAPCs between 1990-2021 in 204 countries and territories. 85
Table S9. Age standardized DALYs of ADOD in people aged ≥65 years in 2021 and their AAPCs between 1990-2021 in 204 countries and territories. 97
Table S10. Sociodemographic-index-related inequalities in prevalence, deaths, and DALYs for ADOD, aged ≥65 years, both sexes 115
Table S11. Case number and ASR prediction of prevalence for ADOD aged ≥65 years from 2022 to 2050 by BAPC models in global, by both sexes 121
Table S12. Case number and ASR prediction of deaths for ADOD aged ≥65 years from 2022 to 2050 by BAPC models in global, by both sexes 125
Table S13. Case number and ASR prediction of DALYs for ADOD aged ≥65 years from 2022 to 2050 by BAPC models in global, by both sexes 129
Figure S1. The changes in the proportion of prevalence cases among ADOD aged over 65 years to the overall ADOD patients from 1990 to 2021. 133
Figure S2. Temporal trend of age-standardized prevalence (A) mortality (B), and DALYs (C) for ADOD patients aged over 65 years and overall ADOD patients from 1990 to 2021. 134
Figure S3. Average annual percent changes of age-standardized prevalence, mortality, and DALYs of ADOD in patients aged over 65 years from 1990 to 2021 by sex and age. 135
Figure S4. Temporal trend of age-standardized prevalence, mortality, and DALYs of ADOD in patients aged over 65 years from 1990 to 2021 at global and socio-demographic index levels by sex. 136
Figure S5. Average annual percent changes of age-standardized prevalence, mortality, and DALYs of ADOD in patients aged over 65 years from 1990 to 2021 at socio-demographic index levels by sex. 137
Figure S6. Prevalence (A), mortality (B), and DALYs (C) rate of ADOD patients aged over 65 years from 204 countries according to the socio-demographic index in 2021 138
Figure S7. Average annual percent changes of age-standardized prevalence (A), mortality (B), and DALYs (C) of ADOD aged over 65 years and overall ADOD patients from 1990 to 2021 at global and socio-demographic index levels. 139
Figure S8. Average annual percent changes of age-standardized prevalence, mortality, and DALYs of ADOD patients aged over 65 years from 1990 to 2021 at regions levels 140
Figure S9. Average annual percent changes of age-standardized prevalence, mortality, and DALYs of ADOD patients aged over 65 years from 1990 to 2021 at regions levels by sex. 141
Figure S10. Association between age-standardised mortality of ADOD among people aged ≥65 years and SDI 142
Figure S11. Association between age-standardised DALYs of ADOD among people aged ≥65 years and SDI 143
STROBE Statement 144

Supplementary Methods

1. GBD Overview

For more than three decades, the Global Burden of Diseases, Injuries, and Risk Factors Study (GBD) has been systematically and comprehensively recording and analysing causes of human death stratified by age, sex, and time across the world[1]. This information has been used to guide policy solutions, reduce modifiable risk factors, monitor and evaluate national and sub-national health interventions, and ultimately improve health recommendations at both regional and local levels. Published in The Lancet in May 2020, GBD 2021 provides, for the first time, an independent estimation of population for each of 204 countries and territories and for the globe using a standardized, replicable approach, as well as a comprehensive update on fertility and migration[2]. GBD 2021 incorporates major data additions and improvements and methodological refinements. Mortality and life expectancy estimates have expanded to a total of 881 locations at the most detailed level, and new causes have been added to the fatal and nonfatal cause lists, for a total of 371 diseases and injuries (https://www.healthdata.org/research-analysis/about-gbd/protocol). GBD 2021 produced estimates for each epidemiological quantity of interest— incidence, prevalence, mortality, years lived with disability (YLDs), years of life lost (YLLs), and disability-adjusted life-years (DALYs)— for 288 causes of death by age-sex-location-year for 25 age groups from birth to 95 years and older; for males, females, and both sexes combined; in 204 countries and territories grouped into 21 regions and seven super-regions; and for every year from 1990 to 2021. GBD 2021 also includes subnational analyses for 21 countries and territories. An international network of collaborators provides, reviews, and analyses the available data to generate these metrics; GBD 2021 drew on the expertise of more than 11 000 collaborators from more than 160 countries and territories. GBD classifies diseases and injuries into a hierarchy with four levels that include both fatal and non-fatal causes. Level 1 causes include three broad aggregate categories (communicable, maternal, neonatal, and nutritional [CMNN] diseases; non-communicable diseases [NCDs]; and injuries) and Level 2 disaggregates those categories into 22 clusters of causes, which are further disaggregated into Level 3 and Level 4 causes. At the most detailed level, 288 fatal causes are estimated[2].

2. Data sources

GBD 2021 synthesises a large and growing number of data input sources including surveys, censuses, vital statistics, and other health-related data sources. The data from these sources are used to estimate morbidity; illness, and injury; and attributable risk for 204 countries and territories from 1990 to 2021; mortality deaths are estimated from 1980 to 2021.

The GBD estimation process is based on identifying multiple relevant data sources for each disease or injury, including censuses, household surveys, civil registration and vital statistics, disease registries, health service use, air pollution monitors, satellite imaging, disease notifications, and other sources. Each of these types of data is identified from a systematic review of published studies, searches of government and international organization websites, published reports, primary data sources such as the Demographic and Health Surveys, and contributions of datasets by GBD collaborators. All data

used in this study were extracted from the Global Health Data Exchange (https://vizhub.healthdata.org/gbd-results/) including (1) global age- and sex- specific prevalence, mortality, DALYs numbers and crude rates (per 100,000 persons) from 1990 to 2021; (2) Regional age- and sex- specific incidence, mortality, DALYs numbers and crude rates from 1990 to 2021 by socio-demographic index (SDI) categories; (3) National age- and sex- specific incidence, mortality, DALYs numbers and crude rates from 1990 to 2021; (4) GBD world standard population in 2021; (5) Age- and sex- specific Alzheimer's disease and other dementias (ADOD) DALYs numbers and crude rates attributable to risk factors (level 4).

3. Calculation methods for DALYs

To estimate DALYs, GBD 2021 started by estimating cause-specific mortality and non- fatal health loss. For each year for which YLDs have been estimated, GBD 2021 computed DALYs by adding YLLs and YLDs for each age-sex-location (Figure 1). Uncertainty in YLLs was assumed to be independent of uncertainty in YLDs. A total of 500 draws for DALYs were computed by initially summing the first instance of the 500 draws for YLLs and YLDs, followed by repeating this process for each subsequent draw. 95% UIs were computed by using the 25th and 975th ordered draw of the DALY uncertainty distribution. DALYs were determined as the aggregate of YLLs and YLDs for every cause, stratified by location, age group, sex, and year. For more information, please refer to the previously reported literature[3].

Figure 1. DALY burden estimation for GBD 2021

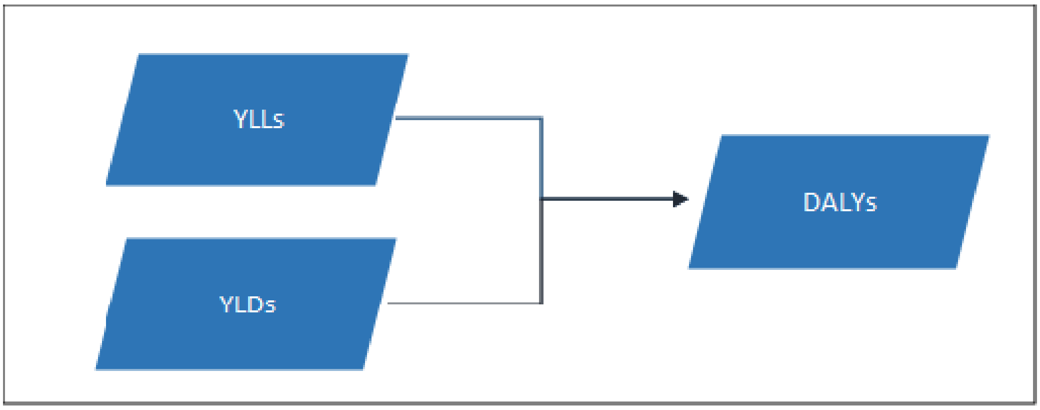

3.1 YLLs

Years of life lost (YLLs) owing to premature mortality were computed for 1082 locations and 39 years. The initial step involved employing the lowest observed age-specific mortality rates by location and sex across all estimation years. These rates were extracted from locations with total populations exceeding 5 million in 2016 to construct a theoretical minimum risk reference life table.

The YLL is a metric that is computed by multiplying the number of estimated deaths by the predicted

life expectancy by age, sex, location, and year. The metric therefore highlights premature deaths by applying a larger weight to deaths that occur in younger age groups. Uncertainty was propagated from the CoDCorrected deaths across all demographic groups. The predicted life expectancy is calculated with two main components, a global, sex-agnostic, all time theoretical "best" life expectancy (assuming that males and females in all countries in the world could theoretically have this life expectancy) and the average age of death from with-shock life tables for each location, sex, age group, and year. Further details on GBD life expectancy values can be found in the previous papers[2,4]. The core equation can be written as follows:

$$YLL = \sum_{c=1,a=o,s=1}^{\infty} d_{cas}\, e_a$$

3.2 YLDs

The critical stage in the estimation of YLDs is a micro-simulation, which adjusts for comorbidity. GBD 2021 refer to this micro-simulation process as "COMO" (for comorbidity correction). For GBD 2021, the co-occurrence of different diseases was estimated by simulating 20,000 individuals in each location-age-sex-year combination, with each individual exposed to the independent probability of having any of the sequelae included in GBD, based on prevalence. Age was the main predictor of comorbidity such that age- specific micro-simulations accommodated most of the required comorbidity correction[5].

The two components necessary for the computation of YLDs and are the two inputs into COMO: 1) prevalence of each disease sequela and 2) DWs. The prevalence values of causes are primarily produced by using DisMod-MR 2.1 and, for causes with multiple sequelae, subsequently apportioned into sequela-specific prevalence based on available estimates of the severity distribution.

The micro-simulation, as performed for each age-sex-location-year, can best be represented as a four-step process. First, simulated individuals (simulants) are exposed to independent probabilities of having each sequela, where the probability is equal to the prevalence estimate. For each simulant, the probability of having a disease sequela is equal to the estimated prevalence. Each simulant is determined to have or not have the disease sequelae based on a draw from a binomial distribution. From this simulation, simulants end up with any number of sequelae, from 0 up to the theoretical maximum given their demographics. Second, the DW for each simulant is estimated on the basis of the disease sequelae that they have acquired. The formula for the cumulative DW for a simulant is one minus the multiplicative sum of one minus each DW present

$$Simulant\ DW_l = 1 - \prod_{k=i}^{j}(1 - \mathrm{DWk})$$

Where:

DWk is the DW for the kth disease sequela that the simulant l has acquired.

Once the simulant DW is computed, the DW attributable to each sequela for the simulant is calculated by using the following formula:

$$ADW_{lk} = \frac{DW_k}{\sum_{k=i}^{k=j} DW_k} * Simulant\ DW_l$$

Where:

$ADWlk$ is the attributable DW for disease sequela k in simulant l

DWk is the DW for disease sequela k

Simulant DW_l is the DW for simulant l from the combination of all sequelae that they have acquired.

This formula apportions the overall simulant DW to each condition in proportion to the DW of each condition in isolation.

Finally, YLDs per capita in an age-sex-country-year are computed by taking the sum of the attributable DWs for a disease sequela across simulants.

$$YLD\ Ratek = \frac{\sum_{l=1}^{n} ADW_{lk}}{n}$$

The actual number of YLDs from disease sequela k in an age-sex-location-year is then computed as the YLD rate k times the appropriate age-sex-location-year population.

4. Estimation of ADOD non-fatal burdens in GBD 2021

4.1 Case definition

Dementia was defined according to WHO criteria as a syndrome that can be caused by a number of diseases that, over time, destroy nerve cells and damage the brain, typically leading to a decline in cognitive function (i.e. the ability to process thoughts) beyond what would be expected from the usual consequences of biological ageing.

Alzheimer disease: Alzheimer disease is a primary degenerative cerebral disease of unknown etiology with characteristic neuropathological and neurochemical features. The disorder is usually insidious in onset and develops slowly but steadily over a period of several years.

Vascular dementia: Vascular dementia is the result of infarction of the brain due to vascular disease,

including hypertensive cerebrovascular disease. The infarcts are usually small but cumulative in their effect. Onset is usually in later life. Include: arteriosclerotic dementia.

Dementia in other diseases classified elsewhere: Cases of dementia due, or presumed to be due, to causes other than Alzheimer disease or cerebrovascular disease. Onset may be at any time in life, though rarely in old age.

Unspecified dementia: Include: Presenile: dementia, psychosis; Primary degenerative dementia; Senile: dementia, psychosis. Exclude: senility.

Table 1. ICD codes used for inclusion of hospital and claims data

Stroke subtype	ICD-9	ICD-10
Alzheimer's disease and other dementias	290-290.9, 294.1-294.9, 331-331.2	F00-F02.0, F02.8-F03.9, G30-G31.1, G31.8-G31.9

4.2 Data seeking and flowchart

A systematic review was performed for ADOD models in GBD 2021 in accordance with PRISMA systematic review guidelines. Relative risk sources were identified through a systematic review using search terms in PubMed2. This yielded 4470 total hits, of which 34 studies were marked for extraction. The utilized search strings is presented below:

The GBD 2021 searched PubMed with the search terms "dementia" or "Alzheimer's disease" and "cohort change" or "time" or "trends". No studies were excluded because of language or year.

4.3 Data inputs and processing

In the GBD studies, the criteria for determining dementia prevalence were aligned with classifications from either the Diagnostic and Statistical Manual of Mental Disorders (DSM), spanning from DSM-III to DSM-5, or the International Classification of Diseases (ICD), ranging from ICD-8 to ICD-10. These classifications were applied by clinical experts during representative surveys. The studies also encompassed data gleaned from various definitions and diagnostic methodologies, with adjustments made to mitigate biases arising from the divergent study attributes. For further details, please refer to previously published papers[2,6].

4.4 Modelling strategy

Disease Modelling Meta-Regression (DisMod-MR) 2.1, a Bayesian compartmental model used extensively in GBD nonfatal modelling, was used to generate initial estimates of dementia prevalence[7]. This model maintains consistency between different parameters, such as prevalence, incidence, remission and mortality, by establishing relationships within a system of differential equations.

Remission was assumed to be zero and incidence to be negligible before the age of 40, given the progressive and chronic nature of dementia and its rarity before this age[8,9]. The Bayesian framework facilitates the estimation of prevalence in data-poor locations by incorporating information from covariates and priors derived from the model fit of higher hierarchical levels (for example, the country prior is informed by the regional model fit). In locations with robust data on both prevalence and incidence, the combined incidence and mortality data suggested a higher prevalence than observed, leading to the decision to prioritise prevalence data over incidence, as incidence is likely to be less accurate due to the gradual onset of the disease. The average severity of disease in prevalent cases is higher than in incident cases, suggesting that measurement error is likely to be lower in the former. Covariates such as smoking prevalence and average years of education for those aged 15 and over were included in the model to aid estimation, particularly in sites with poor or missing data. Priors for the education parameter were set based on meta-analytic results from the literature on the relative risk of dementia per additional year of education. The prevalence model used literature data on mortality to produce initial estimates of dementia mortality, which were then used in the mortality modelling process.

Following the estimation of dementia-related mortality, a subsequent DisMod-MR 2.1 model was run to produce the final prevalence estimates. This model had the same settings as the initial model, but excluded literature data on mortality and included GBD dementia mortality estimates. For further details, please refer to previously published papers[6,10].

5. Estimation of ADOD fatal burdens in GBD 2021

5.1 Flowchart

The flowchart is shown in the following Figure 2.

Figure 2. Estimation of ADOD fatal burdens

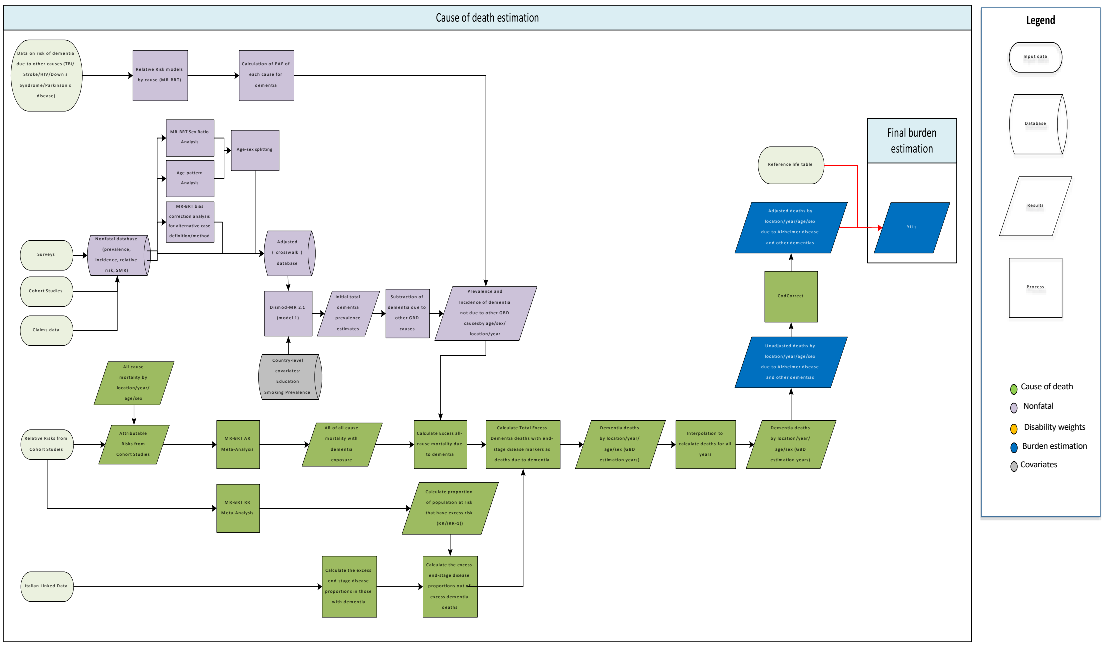

5.2 Data inputs

Two source types were extracted:

(1) Literature on the relative risk of all-cause mortality given the exposure of dementia. Relative risk sources were identified through a systematic review using search terms in PubMed[2,11]. This yielded 4470 total hits, of which 34 studies were marked for extraction. Overall, the data were heterogeneous and varied in the exposure category measured (all dementia, Alzheimer's disease, cognitive impairment) and in the different factors controlled for in analyses.

(2) Linked vital registration and hospitalisation data. GBD 2021 used mortality records linked to inpatient records, covering all deaths from 2003 to 2017 in the Emilia-Romagna region of Italy.

Table 2: Results of systematic review on all-cause excess mortality with dementia

N		60
Region name (%)	East Asia	4 (6.7)
	Eastern sub-Saharan Africa	2 (3.3)
	High-income Asia Pacific	4 (6.7)
	High-income North America	22 (36.7)
	North Africa and Middle East	1 (1.7)
	Tropical Latin America	1 (1.7)
	Western Europe	26 (43.3)
Exposure (%)	Alzheimer's disease	11 (18.3)
	Cognitive impairment	10 (16.7)
	Other dementia	35 (58.3)
	Vascular dementia	4 (6.7)
Conducted in clinical setting (%)	Clinical setting	10 (16.7)

	Population representative	50 (83.3)
Controlled for education (%)	Controlled	32 (53.3)
	No control	28 (46.7)
Controlled for basic CVD info (%)	Controlled	33 (55.0)
	No control	27 (45.0)
Extensive CVD control (%)	Controlled	15 (25.0)
	No control	45 (75.0)
Controlled for smoking and alcohol (%)	Controlled	11 (18.3)
	No control	49 (81.7)
Controlled for factors in causal pathway (%)	Controlled	13 (21.7)
	No control	47 (78.3)

5.3 Modelling strategy

For GBD 2019 onward, the fatal modelling process was redesigned to avoid the need for using estimates only from the highest dementia mortality locations. This was accomplished with an attributable risk model based on a systematic review of cohort studies and relative risk data, and end-stage disease proportions from linked hospital and death records. The modelling process is described below. The modelling process is described below and refer to the previously published paper[2].

Modelling steps

Relative risk data

First, using relative risk data extracted from studies identified by systematic review, GBD 2021 calculated attributable risk and the GBD estimate of all-cause mortality rate for a given study location and time, using the following formula:

$$Attributable\ Risk = (Relative\ Risk - 1) * All\text{-}Cause\ Mortality$$

GBD 2021 then conducted a meta-analysis on the attributable risk data, using covariates for age, sex, exposure category (all dementia, Alzheimer’s disease, cognitive impairment), whether the study was conducted in a clinical sample, and categories indicating different types of variables that were controlled for in the component studies (educational attainment, cardiovascular disease comorbidities, smoking and alcohol consumption, and daily activities or residence in a nursing home). Relative risks were estimated using a second Bayesian bias-reduction meta-regression model and the same studies identified through systematic review. Regression results for relative risk and attributable risk analyses are displayed below.

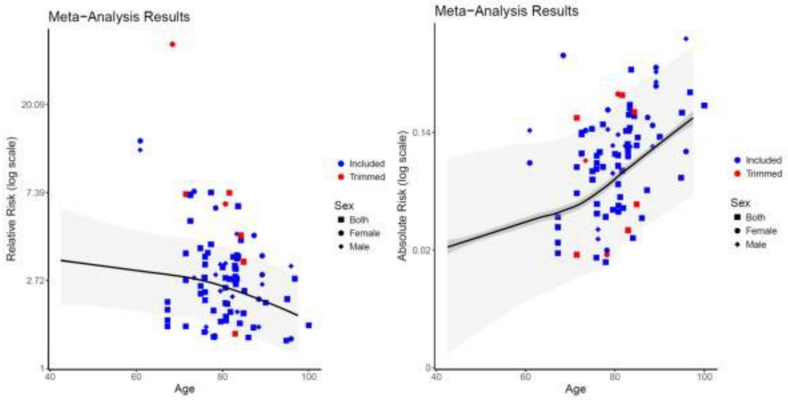

The outcomes from meta-regression analyses were utilized to determine the aggregate excess mortality attributed to dementia, calculated as the multiplication of the prevalence figures (after adjustments for dementia resulting from other GBD-related diseases) and the estimated attributable risks. For specifics on how prevalence was calculated, refer to the section on non-fatal outcomes for dementia.

Integrated Data Analysis

The product of the attributable risk and prevalence yields the overall count of excess deaths associated with dementia, which may encompass fatalities due to other conditions such as cardiovascular diseases. These are more prevalent among individuals with dementia compared to the general population, likely due to shared underlying risk factors like blood pressure issues, smoking habits, and lower levels of education. To isolate the number of deaths directly caused by dementia from the total excess dementia-related deaths, the GBD 2021 conducted an in-depth analysis of integrated clinical and mortality data. This analysis leveraged mortality records linked to hospitalization data, encompassing all fatalities from 2003 to 2017 in Italy's Emilia-Romagna region. Utilizing these datasets, GBD 2021 identified indicators of severe, terminal-phase disease in clinical records up to twelve months prior to death.

For the selection of these indicators, GBD 2021 assessed each ICD code present in the data, calculating the disparity in the proportion of individuals who died with dementia and had documentation of each code in the year preceding death, versus those who died without dementia but had records of the same code in the same time frame. GBD 2021 examined the 150 codes with the most significant differences, opting for codes that signified terminal disease stages, excluding those related to conditions like cardiovascular disease. Codes indicating conditions such as decubitus ulcer, malnutrition, sepsis, pneumonia, urinary tract infections, falls from bed, signs of senility, dehydration, sodium imbalances, muscle wasting, bronchitis, dysphagia, hip fractures, and bedridden status were selected as markers of severe disease.

To ascertain the proportion of excess deaths attributable to dementia, GBD 2021 calculated the proportion of dementia-related deaths that presented clinical markers of terminal disease in the year prior to death, in contrast to the occurrence of such markers among those who died without dementia. The difference in proportions, with end-stage disease markers, between those with and without dementia, represents the estimated fraction of individuals presumed to have died from severe, end-stage

dementia among total deaths within the dementia population.

Determination of Dementia-Attributable Deaths

To apply these findings to the overall excess deaths, GBD 2021 then refined these proportions to determine the fraction of individuals who died with severe, end-stage dementia as part of the excess dementia-related fatalities. This was achieved using the following formula:

$$\frac{Died\ with\ Severe\ Disease}{Excess\ Dementia\ Deaths} = \frac{Died\ with\ Severe\ Disease}{Total\ Dementia\ Deaths} * \frac{Relative\ Risk}{Relative\ Risk - 1}$$

In the GBD 2021 study, the mortality attributed to dementia was derived by multiplying the total excess dementia deaths by the proportion of those who succumbed to severe forms of the disease among the excess dementia fatalities. These refined estimates of dementia-related deaths were subsequently utilized to adjust the data on mortality causes from all other conditions recorded in vital registration systems.

For annual interpolation across all years, GBD 2021 employed log-linear interpolation techniques to project these outcomes (restricted to the years 1990, 1995, 2000, 2005, 2010, 2015, 2017, 2019, 2020, 2021, and 2022) to generate estimates for the complete time span from 1980 to 2022. The Socio-demographic Index served as a covariate for extrapolating the data back to the year 1980.

6. Estimation of ADOD burdens attributable to risk factor in GBD 2021

Four key components are included in the estimation of the burden attributable to a given risk factor: the metric of burden being assessed (the number of deaths, YLLs, YLDs, or DALYs [the sum of YLLs and YLDs]); the exposure levels for a risk factor; the RR of a given outcome due to exposure; and the counterfactual level of risk factor exposure. Uncertainty intervals for attributable burden were calculated using 500 draws for GBD 2021[12,13]. Estimates of attributable burden as DALYs for risk–outcome pairs were generated by using the following model:

$$AB_{jasgt} = \sum_{o=1}^{w} DALY_{joasgt} PAF_{joasgt}$$

Where:

$ABjasgt$ is the attributable burden for risk factor j for age group a, sex s, location g, and year t;

$DALYjoasgt$ is total DALYs for cause o (of w relevant outcomes for risk factor j) for age group a, sex s, location g, and year t;

$PAFjoasgt$ is the PAF for cause o due to risk factor j for age group a, sex s, location g, and year

$t.$

The proportions of deaths, YLLs, or YLDs attributable to a given risk factor or risk factor cluster were analogously computed by sequentially substituting each metric in place of DALYs in the equation provided.

6.1 Definitions of risk factors in GBD 2021

In GBD 2021, the risk factors associated with ADOD are defined as follows[13].

✓ High fasting plasma glucose:

High fasting plasma glucose (FPG) is measured as the mean FPG in a population, where FPG is a continuous exposure in units of mmol/L. Since FPG is along a continuum, GBD 2021 define high FPG as any level above the theoretical minimum-risk exposure level (TMREL), which is 4.9–5.3 mmol/L.

✓ High body-mass index

High body-mass index (BMI) for adults (ages 20+) is defined as BMI greater than 20 to 23 kg/m^2. High BMI for children and adolescents (ages 2–19) is defined as being overweight or obese based on International Obesity Task Force standards.

✓ Smoking

Consistent with prior GBD cycles, the prevalence of current smoking and the prevalence of former smoking was estimated using data sourced from cross-sectional, nationally representative household surveys. Current smokers were identified as individuals partaking in the use of any smoked tobacco product on either a daily or occasional basis. Former smokers were categorized as those who had ceased the use of all smoked tobacco products for a minimum duration of six months, or in accordance with the definition employed by the respective survey, where possible.

7. Estimation of ADOD cross-country health inequality in GBD 2021

Health inequities are the unjust differences in health between persons of different social groups, and can be linked to forms of disadvantage such as poverty, discrimination and lack of access to services or goods. While health inequity is a normative concept, and thus cannot be precisely measured or monitored, health inequality –observable differences between subgroups within a population – can be measured and monitored, and serves as an indirect means of evaluating health inequity. For detailed information on this topic, please consult the section on health inequality on the official website of the World Health Organization[14].

The two most common complex measures to summarize health inequality in a series of subgroups with

a natural ordering are the slope index of inequality (to assess absolute inequality) and the concentration index (to assess relative inequality). A common strength of both of these measures is that their calculation involves weighting by the size of the population, enabling them to yield a single number that describes inequality among all subgroups, taking into account the population size. The detailed calculation principles for these two indicators can be found in the Healthy Inequality Monitoring handbook on the official website of the World Health Organization[15].

7.1 Slope index of inequality

The slope index of inequality is used to show the gradient of health across multiple subgroups with natural ordering (most commonly education or wealth). The slope index of inequality represents the absolute difference in predicted values of a health indicator between those with the highest level of education or wealth and those with the lowest level of education or wealth, while taking into consideration the entire distribution of education or wealth using an appropriate regression model. To calculate the slope index of inequality, a weighted sample of the whole population is ranked from the most disadvantaged subgroup (at rank zero or 0) to the most advantaged (at rank 1) according to, for example, education or wealth. This ranking is weighted, accounting for the proportional distribution of the population within each subgroup. The population of each wealth or education category is then considered in terms of its range in the cumulative population distribution, and the midpoint of this range. Then, the health indicator of interest is regressed against this midpoint value for wealth or education subgroups using an appropriate model, and the predicted values of the health indicator are calculated for the two extremes (rank 1 and rank 0). The difference between the predicted values at rank 1 and rank 0 (covering the entire distribution) generates the slope index of inequality value. Thus, the slope index of inequality represents the difference between the lowest and the highest, while considering all other subgroups in the regression (that is, the effect of change in the whole distribution of population by education or wealth). When the slope of the regression line is flat, the slope index of inequality is 0. When ranking from the most disadvantaged to the most advantaged, positive values indicate that the health indicator of interest is more prevalent in the most advantaged subgroup, whereas negative values mean that the indicator is more prevalent in the most disadvantaged subgroup.

The slope index of inequality value has straightforward meaning and has the same unit of measure as the health indicator, making it very useful. Non-technical audiences can understand the slope index as an estimate of the difference in a given health indicator between the worst-off and best-off individual in a population, though they may initially understand little about how the number is calculated or why it provides an advantage over simple difference.

7.2 Concentration index

The concentration index is a relative measure of inequality that shows the health gradient across multiple subgroups with natural ordering (most commonly education or wealth). It indicates the extent to which a health indicator is concentrated among the disadvantaged or the advantaged. Given that a population is ranked by increasing socioeconomic status, the concentration index has a negative value when the health indicator – whether a favourable indicator such as measles immunization coverage or

an adverse indicator such as under-five mortality – is concentrated among the disadvantaged (for example, the poor or less educated); and it has a positive value when the health indicator is concentrated among the advantaged (for example, the rich or more educated). When there is no inequality, the concentration index is 0. If a single individual (the smallest possible population subgroup) accounted for 100% of a health indicator in a population (the highest relative inequality that is theoretically possible), this would cause the concentration index to approach its maximum absolute value of either –1 or +1. While ±1 is the theoretical maximum of a concentration index, in practice absolute values for the concentration index will rarely exceed 0.5, and a value of 0.2 to 0.3 is considered to represent a reasonably high level of relative inequality.

The visual way to illustrate the concentration index is to use a related concept called the concentration curve. Like the slope index of inequality, it starts with ranking a weighted sample of the whole population from the most-disadvantaged subgroup (at rank 0) to the most-advantaged (at rank 1) according to, for example, education or wealth (x axis). The y axis indicates the cumulative fraction of the health indicator corresponding to each subgroup. The concentration curve is drawn by connecting the dots. The concentration curve lies below the 45° diagonal line from the bottom left corner to the top right – the so-called line of equality – if the health indicator is concentrated among the advantaged; the concentration curve lies above the line of equality if the health indicator is concentrated among the disadvantaged. When there is no inequality, the concentration curve lies on the line of equality. The concentration index is calculated as twice the area between the hypothetical line of equality and the concentration curve.

The slope index is calculated using the Linear Model (LM) function of R or Robust Linear Model (RLM) function (in the event of suspicion of significant heteroscedasticity in the data), following the reorganization of the data in accordance with the guidelines set forth in the Healthy Inequality Monitoring handbook. The concentration index is calculated using the egen_inequal package of Stata[16,17].

8. Predictive analysis of ADOD

In order to forecast the number and incidence of new cases from 2022 through 2050, we used Bayesian age-period-cohort models (BAPC). The BAPC model is founded upon the Age-Period-Cohort (APC) model. The theoretical foundation of this model is the assertion that the incidence or mortality rate is correlated with age structure and population size. The foundation for this analysis is the generalized linear model (GLM).

The application of BAPC models leverages the integrated nested Laplace approximations (INLA) technique to enable a thorough Bayesian analytical process. These models are capable of producing both age-specific and age-standardized predictive rates. In scenarios where the focus is on forecasting, these models inherently incorporate Poisson noise to enhance the predictive distribution's accuracy.

The specific equations and evaluation methods can be found on the R package official website and in previously published literature[18-21].

9. Data accuracy

ADOD is a common and frequent disease worldwide, but only some countries can provide sufficient data. Tables 5 display source count information for fatal ADOD.

This is particularly the case in low-resource regions and countries, resulting in a data gap for first-hand epidemiological studies. GBD 2021 study used the following steps to make the estimates as accurate as possible: (1) compilation of data sources through data identification and extraction; (2) data adjustment; (3) estimation of prevalence and incidence by cause and sequelae by using DisMod-MR 2.1 or alternative modelling strategies for selected cause groups; (4) estimation by impairment; (5) severity distributions; (6) incorporation of disability weights (DWs); (7) adjustment for comorbidity; and (8) the estimation of YLDs by sequelae and causes.

Table 3: Source counts for ADOD in fatal models

Condition	Vital registration and other death data	Number of countries
Alzheimer's disease and other dementias	476	54

9.1 Data extractions in each country

Data pertaining to ischemic stroke from registries and hospital records are assembled globally, encompassing contributions from medical facilities, governmental entities, research surveys, and a spectrum of databases. The Global Burden of Disease (GBD) study compiles this information through a systematic process:

Initially, the GBD Collaborators embark on a comprehensive data collection effort, sourcing health-related data from worldwide hospitals, governmental agencies, research surveys, systematic literature reviews, and various databases. This initial phase is followed by a meticulous data cleaning and organization procedure. Subsequently, the research teams employ the Bayesian meta-regression model, DisMod-MR 2.1, to produce estimates for regions and time periods lacking direct data.

In the final stage, to ensure a thorough compilation of data sources not previously captured in the Global Health Data Exchange (GHDx) or PubMed, the team consults with additional experts in the field. The GBD Network encompasses a diverse array of over 12,000 Collaborators hailing from more than 160 countries, representing a broad spectrum of sectors including research, science, healthcare, policy-making, and international organizations.

The GBD employs the DisMod-MR2.1 model to generate ischemic stroke statistics. It is noteworthy that the original datasets fed into the model are not restricted by age. The GBD refines these estimates through a series of meticulous steps: data cleansing, standardization, adjustment, model development, and

calibration validation.

10. Socio-demographic Index (SDI)

10.1 SDI definition

The Socio-demographic Index (SDI) serves as a comprehensive measure reflecting the underlying societal and economic factors that shape health outcomes across geographic locations. Essentially, it represents the geometric average of three distinct indices ranging from 0 to 1: the total fertility rate (TFR) for individuals under the age of 25 (denoted as TFU25), the average educational attainment for the population aged 15 and above (referred to as EDU15+), and the per capita income that has been subject to a lag distribution (known as LDI). Post-calculation of the SDI for the Global Burden of Disease (GBD) 2021, the derived values were adjusted by scaling them up by a factor of 100 to fit within a range of 0 to 100. This scaling facilitates a more intuitive interpretation and comparison across different regions.

10.2 SDI calculation

For each covariate input, an index score of 0 represents the minimum level of each covariate input past which selected health outcomes can get no worse, and an index score of 1 represents the maximum level of each covariate input past which selected health outcomes cease to improve. As a composite, a location with an SDI of 0 would have a theoretical minimum level of sociodemographic development relevant to these health outcomes, and a location with an SDI of 1 (before multiplying by 100 for reporting) would have a theoretical maximum level of sociodemographic development relevant to these health outcomes.

The index scores that form the basis of the SDI were calculated in the following manner:

$$ICly = \max(\frac{C_{ly} - C_{low}}{C_{high} - C_{low}}, 0.05)$$

Where:

> *ICly* is the index for covariate *C*, location *l*, and year *y* and is equal to the difference between the value of that covariate in that location-year and the lower bound of the covariate divided by the difference between the upper and lower bounds for that covariate.

The composite SDI is the geometric mean of these three indices for a given location-year. For GBD 2021, final SDI values were multiplied by 100 for reporting, in order to improve understanding of and broader engagement with the values[3].

10.3 SDI grouping and quantification

GBD 2021 SDI grouping and quantification are shown in the following tables (Table 4 and Table 5).

Table 3. GBD 2021 SDI groupings by location

Location Name	Level
Addis Ababa	Low SDI
Afar	Low SDI
Afghanistan	Low SDI
Amhara	Low SDI
Balochistan	Low SDI
Bauchi	Low SDI
Benin	Low SDI
Benishangul-Gumuz	Low SDI
Bihar, Rural	Low SDI
Borno	Low SDI
Burkina Faso	Low SDI
Burundi	Low SDI
Central African Republic	Low SDI
Chad	Low SDI
Côte d'Ivoire	Low SDI
Democratic Republic of the Congo	Low SDI
Dire Dawa	Low SDI
Eritrea	Low SDI
Gambella	Low SDI
Gambia	Low SDI
Gilgit-Baltistan	Low SDI
Gombe	Low SDI
Guinea	Low SDI
Guinea-Bissau	Low SDI
Haiti	Low SDI
Harari	Low SDI
Jharkhand, Rural	Low SDI
Jigawa	Low SDI
Kaduna	Low SDI
Kano	Low SDI
Katsina	Low SDI
Kebbi	Low SDI
Khyber Pakhtunkhwa	Low SDI
Liberia	Low SDI
Madagascar	Low SDI
Madhya Pradesh, Rural	Low SDI
Malawi	Low SDI
Mali	Low SDI
Mozambique	Low SDI
Nepal	Low SDI

Niger	Low SDI
Niger	Low SDI
Oromia	Low SDI
Papua New Guinea	Low SDI
Rwanda	Low SDI
Senegal	Low SDI
Sierra Leone	Low SDI
Sokoto	Low SDI
Solomon Islands	Low SDI
Somali	Low SDI
Somalia	Low SDI
South Sudan	Low SDI
Southern Nations, Nationalities, and Peoples	Low SDI
Taraba	Low SDI
Tigray	Low SDI
Timor-Leste	Low SDI
Togo	Low SDI
Uganda	Low SDI
United Republic of Tanzania	Low SDI
Yemen	Low SDI
Yobe	Low SDI
Zamfara	Low SDI
Abia	Low-middle SDI
Abra	Low-middle SDI
Aceh	Low-middle SDI
Acre	Low-middle SDI
Adamawa	Low-middle SDI
Aguascalientes	Low-middle SDI
Agusan Del Norte	Low-middle SDI
Agusan Del Sur	Low-middle SDI
Aklan	Low-middle SDI
Akwa Ibom	Low-middle SDI
Alagoas	Low-middle SDI
Albania	Low-middle SDI
Albay	Low-middle SDI
Alborz	Low-middle SDI
Amazonas	Low-middle SDI
Andhra Pradesh, Rural	Low-middle SDI
Angola	Low-middle SDI
Arunachal Pradesh, Rural	Low-middle SDI
Assam, Rural	Low-middle SDI
Azad Jammu & Kashmir	Low-middle SDI
Bahia	Low-middle SDI
Bangladesh	Low-middle SDI
Baringo	Low-middle SDI
Bayelsa	Low-middle SDI
Belize	Low-middle SDI
Bengkulu	Low-middle SDI

Benue	Low-middle SDI
Bhutan	Low-middle SDI
Bihar, Urban	Low-middle SDI
Bolivia (Plurinational State of)	Low-middle SDI
Bomet	Low-middle SDI
Bungoma	Low-middle SDI
Busia	Low-middle SDI
Cabo Verde	Low-middle SDI
Cambodia	Low-middle SDI
Cameroon	Low-middle SDI
Ceará	Low-middle SDI
Central Java	Low-middle SDI
Central Sulawesi	Low-middle SDI
Chhattisgarh, Rural	Low-middle SDI
Comoros	Low-middle SDI
Congo	Low-middle SDI
Cross River	Low-middle SDI
Delta	Low-middle SDI
Democratic People's Republic of Korea	Low-middle SDI
Djibouti	Low-middle SDI
East Nusa Tenggara	Low-middle SDI
Ebonyi	Low-middle SDI
Egypt	Low-middle SDI
Ekiti	Low-middle SDI
El Salvador	Low-middle SDI
Elgeyo-Marakwet	Low-middle SDI
Embu	Low-middle SDI
Enugu	Low-middle SDI
Eswatini	Low-middle SDI
Garissa	Low-middle SDI
Ghana	Low-middle SDI
Gorontalo	Low-middle SDI
Guatemala	Low-middle SDI
Gujarat, Rural	Low-middle SDI
Haryana, Rural	Low-middle SDI
Homa Bay	Low-middle SDI
Honduras	Low-middle SDI
Isiolo	Low-middle SDI
Jammu & Kashmir and Ladakh, Rural	Low-middle SDI
Kajiado	Low-middle SDI
Kakamega	Low-middle SDI
Karnataka, Rural	Low-middle SDI
Kericho	Low-middle SDI
Kiambu	Low-middle SDI
Kilifi	Low-middle SDI
Kiribati	Low-middle SDI
Kirinyaga	Low-middle SDI
Kisii	Low-middle SDI

Kisumu	Low-middle SDI
Kitui	Low-middle SDI
Kogi	Low-middle SDI
Kwale	Low-middle SDI
Kwara	Low-middle SDI
Kyrgyzstan	Low-middle SDI
Laikipia	Low-middle SDI
Lampung	Low-middle SDI
Lamu	Low-middle SDI
Lao People's Democratic Republic	Low-middle SDI
Lesotho	Low-middle SDI
Machakos	Low-middle SDI
Maharashtra, Rural	Low-middle SDI
Makueni	Low-middle SDI
Maluku	Low-middle SDI
Mandera	Low-middle SDI
Manipur, Rural	Low-middle SDI
Maranhão	Low-middle SDI
Marsabit	Low-middle SDI
Marshall Islands	Low-middle SDI
Mauritania	Low-middle SDI
Meghalaya, Rural	Low-middle SDI
Meru	Low-middle SDI
Micronesia (Federated States of)	Low-middle SDI
Migori	Low-middle SDI
Mizoram, Rural	Low-middle SDI
Mombasa	Low-middle SDI
Mongolia	Low-middle SDI
Morocco	Low-middle SDI
Murang'a	Low-middle SDI
Myanmar	Low-middle SDI
Nagaland, Rural	Low-middle SDI
Nairobi	Low-middle SDI
Nakuru	Low-middle SDI
Namibia	Low-middle SDI
Nandi	Low-middle SDI
Narok	Low-middle SDI
Nasarawa	Low-middle SDI
Nicaragua	Low-middle SDI
North Maluku	Low-middle SDI
Nyamira	Low-middle SDI
Nyandarua	Low-middle SDI
Nyeri	Low-middle SDI
Odisha, Rural	Low-middle SDI
Ogun	Low-middle SDI
Ondo	Low-middle SDI
Other Union Territories, Rural	Low-middle SDI
Oyo	Low-middle SDI

Pará	Low-middle SDI
Paraíba	Low-middle SDI
Pernambuco	Low-middle SDI
Piaui	Low-middle SDI
Plateau	Low-middle SDI
Punjab	Low-middle SDI
Punjab, Rural	Low-middle SDI
Rajasthan, Rural	Low-middle SDI
Rio Grande do Norte	Low-middle SDI
Rondônia	Low-middle SDI
Roraima	Low-middle SDI
Algeria	Middle SDI
Amapá	Middle SDI
Anambra	Middle SDI
Andhra Pradesh, Urban	Middle SDI
Anhui	Middle SDI
Antique	Middle SDI
Apayao	Middle SDI
Ardebil	Middle SDI
Armenia	Middle SDI
Arunachal Pradesh, Urban	Middle SDI
Assam, Urban	Middle SDI
Aurora	Middle SDI
Azerbaijan	Middle SDI
Baja California	Middle SDI
Baja California Sur	Middle SDI
Bali	Middle SDI
Bangka-Belitung Islands	Middle SDI
Banten	Middle SDI
Basilan	Middle SDI
Bataan	Middle SDI
Batanes	Middle SDI
Batangas	Middle SDI
Benguet	Middle SDI
Biliran	Middle SDI
Bohol	Middle SDI
Botswana	Middle SDI
Bukidnon	Middle SDI
Bulacan	Middle SDI
Bushehr	Middle SDI
Cagayan	Middle SDI
Camarines Norte	Middle SDI
Camarines Sur	Middle SDI
Camiguin	Middle SDI
Campeche	Middle SDI
Capiz	Middle SDI
Catanduanes	Middle SDI
Cavite	Middle SDI

Cebu	Middle SDI
Central Kalimantan	Middle SDI
Chahar Mahaal and Bakhtiari	Middle SDI
Chhattisgarh, Urban	Middle SDI
Chiapas	Middle SDI
Chihuahua	Middle SDI
Coahuila	Middle SDI
Colima	Middle SDI
Colombia	Middle SDI
Costa Rica	Middle SDI
Cotabato (North Cotabato)	Middle SDI
Cuba	Middle SDI
Davao de Oro	Middle SDI
Davao Del Norte	Middle SDI
Davao Del Sur	Middle SDI
Davao Occidental	Middle SDI
Davao Oriental	Middle SDI
Delhi, Rural	Middle SDI
Dinagat Islands	Middle SDI
Dominican Republic	Middle SDI
Durango	Middle SDI
East Azarbayejan	Middle SDI
East Java	Middle SDI
Eastern Cape	Middle SDI
Eastern Samar	Middle SDI
Ecuador	Middle SDI
Edo	Middle SDI
Equatorial Guinea	Middle SDI
Espírito Santo	Middle SDI
Fars	Middle SDI
FCT (Abuja)	Middle SDI
Fiji	Middle SDI
Free State	Middle SDI
Gabon	Middle SDI
Gansu	Middle SDI
Gauteng	Middle SDI
Gilan	Middle SDI
Goa, Rural	Middle SDI
Goiás	Middle SDI
Golestan	Middle SDI
Grenada	Middle SDI
Guanajuato	Middle SDI
Guangxi	Middle SDI
Guerrero	Middle SDI
Guimaras	Middle SDI
Guizhou	Middle SDI
Gujarat, Urban	Middle SDI
Guyana	Middle SDI

Hainan	Middle SDI
Hamadan	Middle SDI
Haryana, Urban	Middle SDI
Hebei	Middle SDI
Henan	Middle SDI
Hidalgo	Middle SDI
Himachal Pradesh, Rural	Middle SDI
Hormozgan	Middle SDI
Hubei	Middle SDI
Hunan	Middle SDI
Ifugao	Middle SDI
Ilam	Middle SDI
Ilocos Norte	Middle SDI
Ilocos Sur	Middle SDI
Iloilo	Middle SDI
Imo	Middle SDI
Iraq	Middle SDI
Isabela	Middle SDI
Isfahan	Middle SDI
Islamabad Capital Territory	Middle SDI
Jalisco	Middle SDI
Jamaica	Middle SDI
Jambi	Middle SDI
Jammu & Kashmir and Ladakh, Urban	Middle SDI
Jharkhand, Urban	Middle SDI
Jiangxi	Middle SDI
Kalinga	Middle SDI
Karnataka, Urban	Middle SDI
Kerala, Rural	Middle SDI
Kerala, Urban	Middle SDI
Kerman	Middle SDI
Kermanshah	Middle SDI
Khorasan-e-Razavi	Middle SDI
Khuzestan	Middle SDI
Kohgiluyeh and Boyer-Ahmad	Middle SDI
Kurdistan	Middle SDI
KwaZulu-Natal	Middle SDI
La Union	Middle SDI
Lagos	Middle SDI
Laguna	Middle SDI
Lanao Del Norte	Middle SDI
Lanao Del Sur	Middle SDI
Leyte	Middle SDI
Limpopo	Middle SDI
Lorestan	Middle SDI
Madhya Pradesh, Urban	Middle SDI
Maguindanao	Middle SDI
Maharashtra, Urban	Middle SDI

Maldives	Middle SDI
Manipur, Urban	Middle SDI
Marinduque	Middle SDI
Markazi	Middle SDI
Masbate	Middle SDI
Mato Grosso	Middle SDI
Mato Grosso do Sul	Middle SDI
Mazandaran	Middle SDI
Meghalaya, Urban	Middle SDI
México	Middle SDI
Mexico City	Middle SDI
Michoacán de Ocampo	Middle SDI
Minas Gerais	Middle SDI
Misamis Occidental	Middle SDI
Misamis Oriental	Middle SDI
Mizoram, Urban	Middle SDI
Morelos	Middle SDI
Mountain Province	Middle SDI
Mpumalanga	Middle SDI
Nagaland, Urban	Middle SDI
National Capital Region	Middle SDI
Nauru	Middle SDI
Nayarit	Middle SDI
Negros Occidental	Middle SDI
Negros Oriental	Middle SDI
Ningxia	Middle SDI
North Khorasan	Middle SDI
North Sulawesi	Middle SDI
North Sumatra	Middle SDI
Northern Cape	Middle SDI
Northern Samar	Middle SDI
North-West	Middle SDI
Nueva Ecija	Middle SDI
Nueva Vizcaya	Middle SDI
Nuevo León	Middle SDI
Oaxaca	Middle SDI
Occidental Mindoro	Middle SDI
Odisha, Urban	Middle SDI
Oriental Mindoro	Middle SDI
Osun	Middle SDI
Other Union Territories, Urban	Middle SDI
Palawan	Middle SDI
Palestine	Middle SDI
Pampanga	Middle SDI
Panama	Middle SDI
Pangasinan	Middle SDI
Papua	Middle SDI
Paraguay	Middle SDI

Paraná	Middle SDI
Peru	Middle SDI
Puebla	Middle SDI
Punjab, Urban	Middle SDI
Qazvin	Middle SDI
Qinghai	Middle SDI
Qom	Middle SDI
Querétaro	Middle SDI
Quezon	Middle SDI
Quintana Roo	Middle SDI
Quirino	Middle SDI
Rajasthan, Urban	Middle SDI
Rio de Janeiro	Middle SDI
Rio Grande do Sul	Middle SDI
Rivers	Middle SDI
Rizal	Middle SDI
Romblon	Middle SDI
Saint Lucia	Middle SDI
Saint Vincent and the Grenadines	Middle SDI
Samar (Western Samar)	Middle SDI
Samburu	Middle SDI
Samoa	Middle SDI
San Luis Potosí	Middle SDI
Santa Catarina	Middle SDI
São Paulo	Middle SDI
Sao Tome and Principe	Middle SDI
Sarangani	Middle SDI
Semnan	Middle SDI
Sergipe	Middle SDI
Shanxi	Middle SDI
Siaya	Middle SDI
Sichuan	Middle SDI
Sikkim, Rural	Middle SDI
Sikkim, Urban	Middle SDI
Sinaloa	Middle SDI
Sindh	Middle SDI
Siquijor	Middle SDI
Sistan and Baluchistan	Middle SDI
Sonora	Middle SDI
Sorsogon	Middle SDI
South Cotabato	Middle SDI
South Kalimantan	Middle SDI
South Khorasan	Middle SDI
South Sulawesi	Middle SDI
South Sumatra	Middle SDI
Southeast Sulawesi	Middle SDI
Southern Leyte	Middle SDI
Sri Lanka	Middle SDI

Sudan	Middle SDI
Sultan Kudarat	Middle SDI
Sulu	Middle SDI
Surigao Del Norte	Middle SDI
Surigao Del Sur	Middle SDI
Suriname	Middle SDI
Syrian Arab Republic	Middle SDI
Tabasco	Middle SDI
Taita Taveta	Middle SDI
Tajikistan	Middle SDI
Tamaulipas	Middle SDI
Tamil Nadu, Rural	Middle SDI
Tamil Nadu, Urban	Middle SDI
Tana River	Middle SDI
Tarlac	Middle SDI
Tawi-Tawi	Middle SDI
Tehran	Middle SDI
Telangana, Rural	Middle SDI
Telangana, Urban	Middle SDI
Thailand	Middle SDI
Tharaka Nithi	Middle SDI
Tibet	Middle SDI
Tlaxcala	Middle SDI
Tocantins	Middle SDI
Tokelau	Middle SDI
Tonga	Middle SDI
Trans Nzoia	Middle SDI
Tripura, Rural	Middle SDI
Tripura, Urban	Middle SDI
Tunisia	Middle SDI
Turkana	Middle SDI
Turkmenistan	Middle SDI
Tuvalu	Middle SDI
Uasin Gishu	Middle SDI
Uttar Pradesh, Rural	Middle SDI
Uttar Pradesh, Urban	Middle SDI
Uttarakhand, Rural	Middle SDI
Uzbekistan	Middle SDI
Vanuatu	Middle SDI
Venezuela (Bolivarian Republic of)	Middle SDI
Veracruz de Ignacio de la Llave	Middle SDI
Viet Nam	Middle SDI
Vihiga	Middle SDI
Wajir	Middle SDI
West Azarbayejan	Middle SDI
West Bengal, Rural	Middle SDI
West Bengal, Urban	Middle SDI
West Java	Middle SDI

West Kalimantan	Middle SDI
West Nusa Tenggara	Middle SDI
West Papua	Middle SDI
West Pokot	Middle SDI
West Sulawesi	Middle SDI
West Sumatra	Middle SDI
Western Cape	Middle SDI
Xinjiang	Middle SDI
Yazd	Middle SDI
Yogyakarta	Middle SDI
Yucatán	Middle SDI
Yunnan	Middle SDI
Zacatecas	Middle SDI
Zambales	Middle SDI
Zambia	Middle SDI
Zamboanga Del Norte	Middle SDI
Zamboanga Del Sur	Middle SDI
Zamboanga Sibugay	Middle SDI
Zanjan	Middle SDI
Zimbabwe	Middle SDI
Abruzzo	High-middle SDI
Altai kray	High-middle SDI
American Samoa	High-middle SDI
Amur oblast	High-middle SDI
Antigua and Barbuda	High-middle SDI
Argentina	High-middle SDI
Arkhangelsk oblast without Nenets autonomous district	High-middle SDI
Astrakhan oblast	High-middle SDI
Bahamas	High-middle SDI
Bahrain	High-middle SDI
Barbados	High-middle SDI
Basilicata	High-middle SDI
Belarus	High-middle SDI
Belgorod oblast	High-middle SDI
Bosnia and Herzegovina	High-middle SDI
Brunei Darussalam	High-middle SDI
Bryansk oblast	High-middle SDI
Bulgaria	High-middle SDI
Calabria	High-middle SDI
Campania	High-middle SDI
Chechen Republic	High-middle SDI
Chelyabinsk oblast	High-middle SDI
Chile	High-middle SDI
Chongqing	High-middle SDI
Chukotka Autonomous Area	High-middle SDI
Chuvash Republic	High-middle SDI
Cook Islands	High-middle SDI
Croatia	High-middle SDI

Delhi, Urban	High-middle SDI
Distrito Federal	High-middle SDI
Dominica	High-middle SDI
East Kalimantan	High-middle SDI
Emilia-Romagna	High-middle SDI
Friuli-Venezia Giulia	High-middle SDI
Fujian	High-middle SDI
Georgia	High-middle SDI
Goa, Urban	High-middle SDI
Greece	High-middle SDI
Guam	High-middle SDI
Guangdong	High-middle SDI
Heilongjiang	High-middle SDI
Himachal Pradesh, Urban	High-middle SDI
Hungary	High-middle SDI
Inner Mongolia	High-middle SDI
Irkutsk oblast	High-middle SDI
Israel	High-middle SDI
Ivanovo oblast	High-middle SDI
Jakarta	High-middle SDI
Jewish autonomous oblast	High-middle SDI
Jiangsu	High-middle SDI
Jilin	High-middle SDI
Jordan	High-middle SDI
Kabardino-Balkar Republic	High-middle SDI
Kaliningrad oblast	High-middle SDI
Kaluga oblast	High-middle SDI
Kamchatka kray	High-middle SDI
Karachay-Cherkess Republic	High-middle SDI
Kazakhstan	High-middle SDI
Kemerovo oblast	High-middle SDI
Khabarovsk kray	High-middle SDI
Khanty-Mansi autonomous area	High-middle SDI
Kirov oblast	High-middle SDI
Komi Republic	High-middle SDI
Kostroma oblast	High-middle SDI
Krasnodar kray	High-middle SDI
Krasnoyarsk kray	High-middle SDI
Kurgan oblast	High-middle SDI
Kursk oblast	High-middle SDI
Lazio	High-middle SDI
Lebanon	High-middle SDI
Leningrad oblast	High-middle SDI
Liaoning	High-middle SDI
Libya	High-middle SDI
Liguria	High-middle SDI
Lipetzk oblast	High-middle SDI
Lombardia	High-middle SDI

Magadan oblast	High-middle SDI
Malaysia	High-middle SDI
Malta	High-middle SDI
Marche	High-middle SDI
Mauritius	High-middle SDI
Molise	High-middle SDI
Montenegro	High-middle SDI
Moscow City	High-middle SDI
Moscow oblast	High-middle SDI
Murmansk oblast	High-middle SDI
Nenets autonomous district	High-middle SDI
Niue	High-middle SDI
Nizhny Novgorod oblast	High-middle SDI
North Kalimantan	High-middle SDI
North Macedonia	High-middle SDI
Northern Mariana Islands	High-middle SDI
Novgorod oblast	High-middle SDI
Novosibirsk oblast	High-middle SDI
Oman	High-middle SDI
Omsk oblast	High-middle SDI
Orenburg oblast	High-middle SDI
Oryol oblast	High-middle SDI
Palau	High-middle SDI
Penza oblast	High-middle SDI
Perm kray	High-middle SDI
Piemonte	High-middle SDI
Portugal	High-middle SDI
Primorsky kray	High-middle SDI
Provincia autonoma di Bolzano	High-middle SDI
Provincia autonoma di Trento	High-middle SDI
Pskov oblast	High-middle SDI
Puglia	High-middle SDI
Republic of Adygeya	High-middle SDI
Republic of Altai	High-middle SDI
Republic of Bashkortostan	High-middle SDI
Republic of Buryatia	High-middle SDI
Republic of Crimea	High-middle SDI
Republic of Dagestan	High-middle SDI
Republic of Ingushetia	High-middle SDI
Republic of Kalmykia	High-middle SDI
Republic of Karelia	High-middle SDI
Republic of Khakassia	High-middle SDI
Republic of Mari El	High-middle SDI
Republic of Moldova	High-middle SDI
Republic of Mordovia	High-middle SDI
Republic of North Ossetia-Alania	High-middle SDI
Republic of Sakha (Yakutia)	High-middle SDI
Republic of Tatarstan	High-middle SDI

Republic of Tuva	High-middle SDI
Riau	High-middle SDI
Riau Islands	High-middle SDI
Romania	High-middle SDI
Rostov oblast	High-middle SDI
Ryazan oblast	High-middle SDI
Saint Kitts and Nevis	High-middle SDI
Saint Petersburg	High-middle SDI
Sakhalin oblast	High-middle SDI
Samara oblast	High-middle SDI
Saratov oblast	High-middle SDI
Sardegna	High-middle SDI
Serbia	High-middle SDI
Sevastopol	High-middle SDI
Seychelles	High-middle SDI
Shaanxi	High-middle SDI
Shandong	High-middle SDI
Sicilia	High-middle SDI
Slovakia	High-middle SDI
Smolensk oblast	High-middle SDI
Spain	High-middle SDI
Stavropol kray	High-middle SDI
Sverdlovsk oblast	High-middle SDI
Tambov oblast	High-middle SDI
Tomsk oblast	High-middle SDI
Toscana	High-middle SDI
Trinidad and Tobago	High-middle SDI
Tula oblast	High-middle SDI
Türkiye	High-middle SDI
Tver oblast	High-middle SDI
Tyumen oblast without autonomous areas	High-middle SDI
Udmurt Republic	High-middle SDI
Ukraine (without Crimea & Sevastopol)	High-middle SDI
Ulyanovsk oblast	High-middle SDI
Umbria	High-middle SDI
Uruguay	High-middle SDI
Uttarakhand, Urban	High-middle SDI
Valle d'Aosta	High-middle SDI
Veneto	High-middle SDI
Vladimir oblast	High-middle SDI
Volgograd oblast	High-middle SDI
Vologda oblast	High-middle SDI
Voronezh oblast	High-middle SDI
Yamalo-Nenets autonomous area	High-middle SDI
Yaroslavl oblast	High-middle SDI
Zabaikalsk kray	High-middle SDI
Zhejiang	High-middle SDI
Agder	High SDI

Aichi	High SDI
Akita	High SDI
Alabama	High SDI
Alaska	High SDI
Andorra	High SDI
Aomori	High SDI
Arizona	High SDI
Arkansas	High SDI
Australia	High SDI
Austria	High SDI
Barking and Dagenham	High SDI
Barnet	High SDI
Barnsley	High SDI
Bath and North East Somerset	High SDI
Bedford	High SDI
Beijing	High SDI
Belgium	High SDI
Bermuda	High SDI
Bexley	High SDI
Birmingham	High SDI
Blackburn with Darwen	High SDI
Blackpool	High SDI
Bolton	High SDI
Bournemouth	High SDI
Bracknell Forest	High SDI
Bradford	High SDI
Brent	High SDI
Brighton and Hove	High SDI
Bristol, City of	High SDI
Bromley	High SDI
Buckinghamshire	High SDI
Bury	High SDI
Calderdale	High SDI
California	High SDI
Camden	High SDI
Canada	High SDI
Central Bedfordshire	High SDI
Cheshire East	High SDI
Cheshire West and Chester	High SDI
Chiba	High SDI
Colorado	High SDI
Connecticut	High SDI
Cornwall	High SDI
County Durham	High SDI
Coventry	High SDI
Croydon	High SDI
Cumbria	High SDI
Cyprus	High SDI

Czechia	High SDI
Darlington	High SDI
Delaware	High SDI
Denmark	High SDI
Derby	High SDI
Derbyshire	High SDI
Devon	High SDI
District of Columbia	High SDI
Dolnośląskie	High SDI
Doncaster	High SDI
Dorset	High SDI
Dudley	High SDI
Ealing	High SDI
East Riding of Yorkshire	High SDI
East Sussex	High SDI
Ehime	High SDI
Enfield	High SDI
Essex	High SDI
Estonia	High SDI
Finland	High SDI
Florida	High SDI
France	High SDI
Fukui	High SDI
Fukuoka	High SDI
Fukushima	High SDI
Gateshead	High SDI
Georgia	High SDI
Germany	High SDI
Gifu	High SDI
Gloucestershire	High SDI
Greenland	High SDI
Greenwich	High SDI
Gunma	High SDI
Hackney	High SDI
Halton	High SDI
Hammersmith and Fulham	High SDI
Hampshire	High SDI
Haringey	High SDI
Harrow	High SDI
Hartlepool	High SDI
Havering	High SDI
Hawaii	High SDI
Herefordshire, County of	High SDI
Hertfordshire	High SDI
Hillingdon	High SDI
Hiroshima	High SDI
Hokkaidō	High SDI
Hong Kong Special Administrative Region of China	High SDI

Hounslow	High SDI
Hyōgo	High SDI
Ibaraki	High SDI
Iceland	High SDI
Idaho	High SDI
Illinois	High SDI
Indiana	High SDI
Innlandet	High SDI
Iowa	High SDI
Ireland	High SDI
Ishikawa	High SDI
Isle of Wight	High SDI
Islington	High SDI
Iwate	High SDI
Kagawa	High SDI
Kagoshima	High SDI
Kanagawa	High SDI
Kansas	High SDI
Kensington and Chelsea	High SDI
Kent	High SDI
Kentucky	High SDI
Kingston upon Hull, City of	High SDI
Kingston upon Thames	High SDI
Kirklees	High SDI
Knowsley	High SDI
Kōchi	High SDI
Kujawsko-Pomorskie	High SDI
Kumamoto	High SDI
Kuwait	High SDI
Kyōto	High SDI
Lambeth	High SDI
Lancashire	High SDI
Latvia	High SDI
Leeds	High SDI
Leicester	High SDI
Leicestershire	High SDI
Lewisham	High SDI
Lincolnshire	High SDI
Lithuania	High SDI
Liverpool	High SDI
Łódzkie	High SDI
Louisiana	High SDI
Lubelskie	High SDI
Lubuskie	High SDI
Luton	High SDI
Luxembourg	High SDI
Macao Special Administrative Region of China	High SDI
Maine	High SDI

Małopolskie	High SDI
Manchester	High SDI
Maryland	High SDI
Massachusetts	High SDI
Mazowieckie	High SDI
Medway	High SDI
Merton	High SDI
Michigan	High SDI
Middlesbrough	High SDI
Mie	High SDI
Milton Keynes	High SDI
Minnesota	High SDI
Mississippi	High SDI
Missouri	High SDI
Miyagi	High SDI
Miyazaki	High SDI
Monaco	High SDI
Montana	High SDI
Møre og Romsdal	High SDI
Nagano	High SDI
Nagasaki	High SDI
Nara	High SDI
Nebraska	High SDI
Netherlands	High SDI
Nevada	High SDI
New Hampshire	High SDI
New Jersey	High SDI
New Mexico	High SDI
New York	High SDI
New Zealand non-Maori population	High SDI
Newcastle upon Tyne	High SDI
Newham	High SDI
Niigata	High SDI
Nordland	High SDI
Norfolk	High SDI
North Carolina	High SDI
North Dakota	High SDI
North East Lincolnshire	High SDI
North Lincolnshire	High SDI
North Somerset	High SDI
North Tyneside	High SDI
North Yorkshire	High SDI
Northamptonshire	High SDI
Northern Ireland	High SDI
Northumberland	High SDI
Nottingham	High SDI
Nottinghamshire	High SDI
Ohio	High SDI

Ōita	High SDI
Okayama	High SDI
Okinawa	High SDI
Oklahoma	High SDI
Oldham	High SDI
Opolskie	High SDI
Oregon	High SDI
Ōsaka	High SDI
Oslo	High SDI
Oxfordshire	High SDI
Pennsylvania	High SDI
Peterborough	High SDI
Plymouth	High SDI
Podkarpackie	High SDI
Podlaskie	High SDI
Pomorskie	High SDI
Poole	High SDI
Portsmouth	High SDI
Puerto Rico	High SDI
Qatar	High SDI
Reading	High SDI
Redbridge	High SDI
Redcar and Cleveland	High SDI
Republic of Korea	High SDI
Rhode Island	High SDI
Richmond upon Thames	High SDI
Rochdale	High SDI
Rogaland	High SDI
Rotherham	High SDI
Rutland	High SDI
Saga	High SDI
Saitama	High SDI
Salford	High SDI
San Marino	High SDI
Sandwell	High SDI
Saudi Arabia	High SDI
Scotland	High SDI
Sefton	High SDI
Shanghai	High SDI
Sheffield	High SDI
Shiga	High SDI
Shimane	High SDI
Shizuoka	High SDI
Shropshire	High SDI
Singapore	High SDI
Śląskie	High SDI
Slough	High SDI
Slovenia	High SDI

Solihull	High SDI
Somerset	High SDI
South Carolina	High SDI
South Dakota	High SDI
South Gloucestershire	High SDI
South Tyneside	High SDI
Southampton	High SDI
Southend-on-Sea	High SDI
Southwark	High SDI
St Helens	High SDI
Staffordshire	High SDI
Stockholm	High SDI
Stockport	High SDI
Stockton-on-Tees	High SDI
Stoke-on-Trent	High SDI
Suffolk	High SDI
Sunderland	High SDI
Surrey	High SDI
Sutton	High SDI
Sweden except Stockholm	High SDI
Świętokrzyskie	High SDI
Swindon	High SDI
Switzerland	High SDI
Taiwan (Province of China)	High SDI
Tameside	High SDI
Telford and Wrekin	High SDI
Tennessee	High SDI
Texas	High SDI
Thurrock	High SDI
Tianjin	High SDI
Tochigi	High SDI
Tokushima	High SDI
Tōkyō	High SDI
Torbay	High SDI
Tottori	High SDI
Tower Hamlets	High SDI
Toyama	High SDI
Trafford	High SDI
Troms og Finnmark	High SDI
Trøndelag	High SDI
United Arab Emirates	High SDI
United States Virgin Islands	High SDI
Utah	High SDI
Vermont	High SDI
Vestfold og Telemark	High SDI
Vestland	High SDI
Viken	High SDI
Virginia	High SDI

Wakayama	High SDI
Wakefield	High SDI
Wales	High SDI
Walsall	High SDI
Waltham Forest	High SDI
Wandsworth	High SDI
Warmińsko-Mazurskie	High SDI
Warrington	High SDI
Warwickshire	High SDI
Washington	High SDI
West Berkshire	High SDI
West Sussex	High SDI
West Virginia	High SDI
Westminster	High SDI
Wielkopolskie	High SDI
Wigan	High SDI
Wiltshire	High SDI
Windsor and Maidenhead	High SDI
Wirral	High SDI
Wisconsin	High SDI
Wokingham	High SDI
Wolverhampton	High SDI
Worcestershire	High SDI
Wyoming	High SDI
Yamagata	High SDI
Yamaguchi	High SDI
Yamanashi	High SDI
York	High SDI
Zachodniopomorskie	High SDI

Table 5. GBD 2021 SDI quantification

Location Name	Value
Abra	0.654174
Abruzzo	0.816497
Aceh	0.671759
Acre	0.562075
Addis Ababa	0.695288
Afar	0.286503
Afghanistan	0.335068
Agder	0.907093
Aguascalientes	0.682557
Agusan Del Norte	0.615708
Agusan Del Sur	0.541666
Aichi	0.883329
Akita	0.832381
Aklan	0.640421

Alabama	0.826418
Alagoas	0.529743
Alaska	0.857126
Albania	0.706889
Albay	0.640151
Alborz	0.748209
Algeria	0.65972
AmapÃ¡	0.629808
Amazonas	0.603586
American Samoa	0.726268
Amhara	0.322156
Andorra	0.869895
Angola	0.482946
Antigua and Barbuda	0.74985
Antique	0.569823
ÅOEita	0.848716
ÅOEsaka	0.876433
Aomori	0.828694
Apayao	0.607701
Ardebil	0.658778
Argentina	0.733528
Arizona	0.847683
Arkansas	0.816754
Armenia	0.702497
Aurora	0.614063
Australia	0.844269
Austria	0.854558
Azad Jammu & Kashmir	0.541343
Azerbaijan	0.695411
B angka-Belitung Islands	0.644388
B uckinghamshire	0.888192
Bahamas	0.805144
Bahia	0.574142
Bahrain	0.752218
Baja California	0.704777
Baja California Sur	0.710175
Bali	0.652383
Balochistan	0.41711
Bangladesh	0.493106
Banten	0.641544
Barbados	0.747065
Baringo	0.514622
Barking and Dagenham	0.806426
Barnet	0.885111
Barnsley	0.788112
Basilan	0.546109
Basilicata	0.783798
Bataan	0.660374

Batanes	0.682566
Batangas	0.686055
Bath and North East Somerset	0.895076
Bedford	0.856962
Belarus	0.784114
Belgium	0.853674
Belize	0.610552
Bengkulu	0.613885
Benguet	0.716293
Benin	0.374522
Benishangul-Gumuz	0.323655
Bermuda	0.82132
Bexley	0.844098
Bhutan	0.476725
Biliran	0.643028
Birmingham	0.836949
Blackburn with Darwen	0.810134
Blackpool	0.788905
Bohol	0.604934
Bolivia (Plurinational State of)	0.604497
Bolton	0.812596
Bomet	0.530036
Bosnia and Herzegovina	0.722964
Botswana	0.643078
Bournemouth	0.87015
Bracknell Forest	0.890716
Bradford	0.814924
Brent	0.858684
Brighton and Hove	0.897901
Bristol, City of	0.896566
Bromley	0.869194
Brunei Darussalam	0.810289
Bukidnon	0.551059
Bulacan	0.708003
Bulgaria	0.764641
Bungoma	0.488275
Burkina Faso	0.284471
Burundi	0.291289
Bury	0.828817
Bushehr	0.708677
Busia	0.47817
CÃ´te d'Ivoire	0.424541
Cabo Verde	0.533601
Cagayan	0.634333
Calabria	0.775305
Calderdale	0.836996
California	0.87109
Camarines Norte	0.594459

Camarines Sur	0.633744
Cambodia	0.474
Cambridgeshire	0.88763
Camden	0.936076
Cameroon	0.480365
Camiguin	0.63209
Campania	0.766832
Campeche	0.665088
Canada	0.873182
Capiz	0.5714
Catanduanes	0.609423
Cavite	0.729179
CearÃ¡	0.563913
Cebu	0.658155
Central African Republic	0.311027
Central Bedfordshire	0.851319
Central Java	0.61378
Central Kalimantan	0.639931
Central Sulawesi	0.617545
Chad	0.243517
Chahar Mahaal and Bakhtiari	0.678339
Cheshire East	0.884279
Cheshire West and Chester	0.870242
Chiapas	0.569757
Chiba	0.861645
Chihuahua	0.674472
Chile	0.770149
Coahuila	0.678075
Colima	0.699338
Colombia	0.65664
Colorado	0.875986
Comoros	0.476956
Congo	0.586909
Connecticut	0.901972
Cook Islands	0.778252
Cornwall	0.839219
Costa Rica	0.70437
Cotabato (North Cotabato)	0.552112
County Durham	0.810755
Coventry	0.847336
Croatia	0.799069
Croydon	0.85144
Cuba	0.669332
Cumbria	0.842326
Cyprus	0.835649
Czechia	0.82851
Darlington	0.835428
Davao de Oro	0.533099

Davao Del Norte	0.636971
Davao Del Sur	0.659918
Davao Occidental	0.578315
Davao Oriental	0.547237
Delaware	0.866079
Democratic People's Republic of Korea	0.569455
Democratic Republic of the Congo	0.390178
Denmark	0.897314
Derby	0.844077
Derbyshire	0.823175
Devon	0.854622
Dinagat Islands	0.622041
Dire Dawa	0.542618
District of Columbia	0.907427
Distrito Federal	0.776152
Djibouti	0.4892
Dominica	0.747382
Dominican Republic	0.619171
Doncaster	0.793628
Dorset	0.851417
Dudley	0.802586
Durango	0.640563
Ealing	0.882007
East Azarbayejan	0.667934
East Java	0.64654
East Kalimantan	0.761652
East Nusa Tenggara	0.550546
East Riding of Yorkshire	0.835194
East Sussex	0.838987
Eastern Cape	0.619101
Eastern Samar	0.4998
Ecuador	0.665675
Egypt	0.603962
Ehime	0.84378
El Salvador	0.56557
Elgeyo -Marakwet	0.525285
Embu	0.548378
Emilia-Romagna	0.829695
Enfield	0.845517
Equatorial Guinea	0.663978
Eritrea	0.404572
EspÃ-rito Santo	0.667429
Essex	0.844953
Estonia	0.845787
Eswatini	0.586217
Fars	0.715109
Fiji	0.669069
Finland	0.860244

Florida	0.861825
France	0.837816
Free State	0.678894
Friuli-Venezia Giulia	0.819858
Fukui	0.85658
Fukuoka	0.858415
Fukushima	0.841218
Gabon	0.639081
Gambella	0.460576
Gambia	0.410077
Garissa	0.32165
Gateshead	0.82858
Gauteng	0.736905
Georgia	0.733124
Georgia	0.847268
Germany	0.903516
Ghana	0.563348
Gifu	0.853448
Gilan	0.712362
Gilgit-Baltistan	0.399312
Gloucestershire	0.870918
GoiÃ¡s	0.639348
Golestan	0.656422
Gorontalo	0.57105
Greece	0.791882
Greenland	0.83564
Greenwich	0.845444
Grenada	0.669351
Guam	0.802168
Guanajuato	0.647045
Guatemala	0.540099
Guerrero	0.584127
Guimaras	0.60986
Guinea	0.336555
Guinea-Bissau	0.353448
Gunma	0.86177
Guyana	0.650902
Hackney	0.891329
Haiti	0.448751
Halton	0.835688
Hamadan	0.666969
Hammersmith and Fulham	0.934892
Hampshire	0.871999
Harari	0.53974
Haringey	0.871466
Harrow	0.858245
Hartlepool	0.797297
Havering	0.834177

Hawaii	0.87084
Herefordshire, County of	0.846195
Hertfordshire	0.886963
Hidalgo	0.633128
Hillingdon	0.892536
Hiroshima	0.87017
HokkaidA	0.844009
Homa Bay	0.507695
Honduras	0.513586
Hormozgan	0.670775
Hounslow	0.896361
Hungary	0.791025
HyÅg o	0.868491
Ibaraki	0.860523
Iceland	0.874629
Idaho	0.836495
Ifugao	0.597326
Ilam	0.705185
Illinois	0.880611
Ilocos Norte	0.687657
Ilocos Sur	0.671837
Iloilo	0.673508
Indiana	0.844051
Innlandet	0.899977
Iowa	0.864342
Iraq	0.662777
Ireland	0.87399
Isabela	0.636784
Isfahan	0.709894
Ishikawa	0.860215
Isiolo	0.435134
Islamabad Capital Territory	0.695559
Isle of Wight	0.826024
Islington	0.924303
Israel	0.809091
Iwate	0.835653
Jakarta	0.801238
Jalisco	0.677078
Jamaica	0.683064
Jambi	0.640779
Jordan	0.72542
KAchi	0.83589
Kagawa	0.857948
Kagoshima	0.832472
Kajiado	0.501635
Kakamega	0.508519
Kalinga	0.575239
Kanagawa	0.882744

Kansas	0.858891
Kazakhstan	0.718332
Kensington and Chelsea	0.946366
Kent	0.844501
Kentucky	0.821721
Kericho	0.520412
Kerman	0.668784
Kermanshah	0.674511
Khorasan-e-Razavi	0.67054
Khuzestan	0.669817
Khyber Pakhtunkhwa	0.451366
Kiambu	0.593046
Kilifi	0.486687
Kingston upon Hull, City of	0.797611
Kingston upon Thames	0.908394
Kiribati	0.525958
Kirinyaga	0.546741
Kirklees	0.823525
Kisii	0.549399
Kisumu	0.548721
Kitui	0.474177
Knowsley	0.811985
Kohgiluyeh and Boyer-Ahmad	0.694488
Kumamoto	0.834728
Kurdistan	0.642334
Kuwait	0.846802
Kwale	0.479657
KwaZulu-Natal	0.662386
KyÅt o	0.876289
Kyrgyzstan	0.609181
La Union	0.661006
Laguna	0.70162
Laikipia	0.576687
Lambeth	0.916016
Lampung	0.608913
Lamu	0.505575
Lanao Del Norte	0.587617
Lanao Del Sur	0.53291
Lancashire	0.839626
Lao People's Democratic Republic	0.489281
Latvia	0.830715
Lazio	0.827196
Lebanon	0.741226
Leeds	0.86788
Leicester	0.828051
Leicestershire	0.851101
Lesotho	0.511571
Lewisham	0.856729

Leyte	0.611702
Liberia	0.353229
Libya	0.735084
Liguria	0.821521
Limpopo	0.613432
Lincolnshire	0.820523
Lithuania	0.857613
Liverpool	0.847483
Lombardia	0.829091
Lorestan	0.669421
Louisiana	0.82667
Luton	0.838003
Luxembourg	0.884636
MÃ¸re og Romsdal	0.908149
MÃ©xico	0.681505
Machakos	0.550158
Madagascar	0.401385
Maguindanao	0.510702
Maine	0.866793
Makueni	0.514231
Malawi	0.381986
Malaysia	0.742553
Maldives	0.657665
Mali	0.271176
Malta	0.801854
Maluku	0.581624
Manchester	0.880573
Mandera	0.239927
MaranhÃ£o	0.492162
Marche	0.803749
Marinduque	0.54965
Markazi	0.682789
Marsabit	0.399977
Marshall Islands	0.573525
Maryland	0.891056
Masbate	0.45806
Massachusetts	0.90725
Mato Grosso	0.647044
Mato Grosso do Sul	0.642693
Mauritania	0.495267
Mauritius	0.717977
Mazandaran	0.729936
Medway	0.819462
Merton	0.887252
Meru	0.509152
Mexico City	0.759378
Michigan	0.864941
MichoacAjn de Ocampo	0.613949

Micronesia (Federated States of)	0.588013
Middlesbrough	0.798977
Mie	0.860795
Migori	0.482207
Milton Keynes	0.886756
Minas Gerais	0.648905
Minnesota	0.887884
Misamis Occidental	0.588185
Misamis Oriental	0.662873
Mississippi	0.811867
Missouri	0.849044
Miyagi	0.860059
Miyazaki	0.826769
Molise	0.787791
Mombasa	0.598167
Monaco	0.909519
Mongolia	0.618744
Montana	0.859517
Montenegro	0.796533
Morelos	0.670105
Morocco	0.56168
Mountain Province	0.519219
Mozambique	0.327475
Mpumalanga	0.648325
Murang'a	0.552922
Myanmar	0.528492
Nagano	0.858967
Nagasaki	0.829106
Nairobi	0.684189
Nakuru	0.572109
Namibia	0.618074
Nandi	0.51604
Nara	0.851417
Narok	0.458227
National Capital Region	0.751536
Nauru	0.62755
Nayarit	0.657929
Nebraska	0.865629
Negros Occidental	0.604235
Negros Oriental	0.578181
Nepal	0.433953
Netherlands	0.888376
Nevada	0.847864
New Hampshire	0.898526
New Jersey	0.891851
New Mexico	0.832846
New York	0.885926
Newcastle upon Tyne	0.871588

Newham	0.840478
Nicaragua	0.523647
Niger	0.17031
Niigata	0.845234
Niue	0.726219
Nordland	0.898214
Norfolk	0.836989
North Carolina	0.846174
North Dakota	0.876135
North East Lincolnshire	0.803564
North Kalimantan	0.754016
North Khorasan	0.651483
North Lincolnshire	0.824583
North Macedonia	0.750955
North Maluku	0.563445
North Somerset	0.858649
North Sulawesi	0.652615
North Sumatra	0.669499
North Tyneside	0.835248
North Yorkshire	0.855521
Northamptonshire	0.839033
Northern Cape	0.665813
Northern Ireland	0.84153
Northern Mariana Islands	0.777505
Northern Samar	0.523968
Northumberland	0.822334
North-West	0.654616
Nottingham	0.858455
Nottinghamshire	0.822433
Nueva Ecija	0.650592
Nueva Vizcaya	0.61661
Nuevo LeÃ³n	0.712153
Nyamira	0.593029
Nyandarua	0.57754
Nyeri	0.579363
Oaxaca	0.588389
Occidental Mindoro	0.460289
Ohio	0.851227
Okayama	0.86235
Okinawa	0.82164
Oklahoma	0.828145
Oldham	0.796705
Oman	0.773801
Oregon	0.87019
Oriental Mindoro	0.605552
Oromia	0.337961
Oslo	0.947286
Oxfordshire	0.899135

Palau	0.75459
Palawan	0.527211
Palestine	0.629202
Pampanga	0.697271
Panama	0.70666
Pangasinan	0.666136
Papua	0.646554
Papua New Guinea	0.418098
ParÃ¡	0.577314
ParaÃ-ba	0.557922
Paraguay	0.650488
ParanÃ¡	0.669861
Pennsylvania	0.87395
Pernambuco	0.583215
Peru	0.662036
Peterborough	0.837008
Piaui	0.520292
Piemonte	0.806813
Plymouth	0.842177
Poole	0.862991
Portsmouth	0.864778
Portugal	0.745395
Provincia autonoma di Bolzano	0.838907
Provincia autonoma di Trento	0.829532
Puebla	0.622885
Puerto Rico	0.824544
Puglia	0.763947
Punjab	0.520053
Qatar	0.846704
Qazvin	0.687516
Qom	0.694134
QuerÃ©taro	0.684048
Quezon	0.630169
Quintana Roo	0.682591
Quirino	0.575885
Reading	0.905272
Redbridge	0.849278
Redcar and Cleveland	0.79636
Republic of Korea	0.887196
Republic of Moldova	0.732393
Rhode Island	0.884284
Riau	0.724215
Riau Islands	0.749803
Richmond upon Thames	0.932022
Rio de Janeiro	0.710471
Rio Grande do Norte	0.585328
Rio Grande do Sul	0.689723
Rizal	0.710524

Rochdale	0.800322
Rogaland	0.916765
Romania	0.766321
Romblon	0.527573
RondÃ´nia	0.61851
Roraima	0.609883
Rotherham	0.803331
Rutland	0.852196
Rwanda	0.43614
SÃ£o Paulo	0.711183
Saga	0.835873
Saint Kitts and Nevis	0.756333
Saint Lucia	0.672602
Saint Vincent and the Grenadines	0.640887
Saitama	0.856554
Salford	0.837393
Samar (Western Samar)	0.527591
Samburu	0.371476
Samoa	0.59234
San Luis PotosÃ-	0.64758
San Marino	0.887884
Sandwell	0.793669
Santa Catarina	0.69484
Sao Tome and Principe	0.503306
Sarangani	0.582419
Sardegna	0.772644
Saudi Arabia	0.814516
Scotland	0.853887
Sefton	0.825438
Semnan	0.724002
Senegal	0.409005
Serbia	0.792213
Sergipe	0.59059
Seychelles	0.727579
Sheffield	0.854247
Shiga	0.874378
Shimane	0.838874
Shizuoka	0.865721
Shropshire	0.84238
Siaya	0.484422
Sicilia	0.762559
Sierra Leone	0.359009
Sinaloa	0.678348
Sindh	0.513737
Singapore	0.856235
Siquijor	0.600945
Sistan and Baluchistan	0.549869
Slough	0.877374

Slovakia	0.808329
Slovenia	0.842633
Solihull	0.871567
Solomon Islands	0.429542
Somali	0.270148
Somalia	0.077434
Somerset	0.842821
Sonora	0.709904
Sorsogon	0.600776
South Carolina	0.838586
South Cotabato	0.632537
South Dakota	0.856264
South Gloucestershire	0.884119
South Kalimantan	0.622222
South Khorasan	0.653365
South Sudan	0.278378
South Sulawesi	0.622994
South Sumatra	0.646421
South Tyneside	0.79949
Southampton	0.860211
Southeast Sulawesi	0.618325
Southend-on-Sea	0.825019
Southern Leyte	0.601491
Southern Nations, Nationalities, and Peoples	0.357248
Southwark	0.919165
Spain	0.769483
Sri Lanka	0.701372
St Helens	0.810914
Staffordshire	0.828182
Stockholm	0.916765
Stockport	0.861015
Stockton-on-Tees	0.829518
Stoke-on-Trent	0.796442
Sudan	0.542748
Suffolk	0.840389
Sultan Kudarat	0.519757
Sulu	0.483999
Sunderland	0.817933
Surigao Del Norte	0.627546
Surigao Del Sur	0.587487
Suriname	0.641163
Surrey	0.904484
Sutton	0.85753
Sweden except Stockholm	0.875759
Swindon	0.866702
Switzerland	0.933532
Syrian Arab Republic	0.622856
TÃ¼rkiye	0.713246

Tabasco	0.649851
Taita Taveta	0.542579
Taiwan (Province of China)	0.87514
Tajikistan	0.536613
TÅk yÅ	0.929043
Tamaulipas	0.682912
Tameside	0.798558
Tana River	0.389359
Tarlac	0.65076
Tawi-Tawi	0.535621
Tehran	0.776103
Telford and Wrekin	0.826094
Tennessee	0.831969
Texas	0.836777
Thailand	0.682657
Tharaka Nithi	0.528625
Thurrock	0.81863
Tigray	0.384003
Timor-Leste	0.450689
Tlaxcala	0.648989
Tocantins	0.601403
Tochigi	0.861256
Togo	0.410016
Tokelau	0.687018
Tokushima	0.857298
Tonga	0.629101
Torbay	0.81246
Toscana	0.811485
Tottori	0.836091
Tower Hamlets	0.903701
Toyama	0.865454
TrÃ¸ndelag	0.916774
Trafford	0.896679
Trans Nzoia	0.549785
Trinidad and Tobago	0.769401
Troms og Finnmark	0.904454
Tunisia	0.681701
Turkana	0.368386
Turkmenistan	0.68304
Tuvalu	0.578627
Uasin Gishu	0.567923
Uganda	0.426554
Umbria	0.799092
United Arab Emirates	0.84974
United Republic of Tanzania	0.448566
United States Virgin Islands	0.822988
Uruguay	0.721713
Utah	0.854829

Uzbekistan	0.664965
Valle d'Aosta	0.812706
Vanuatu	0.472796
Veneto	0.808197
Venezuela (Bolivarian Republic of)	0.5966
Veracruz de Ignacio de la Llave	0.627367
Vermont	0.891522
Vestfold og Telemark	0.907352
Vestland	0.917642
Viet Nam	0.621621
Vihiga	0.527055
Viken	0.914602
Virginia	0.881907
Wajir	0.258714
Wakayama	0.84733
Wakefield	0.804507
Wales	0.833275
Walsall	0.790655
Waltham Forest	0.840039
Wandsworth	0.924121
Warrington	0.878129
Warwickshire	0.865694
Washington	0.878014
West Azarbayejan	0.626919
West Berkshire	0.897125
West Java	0.644279
West Kalimantan	0.587439
West Nusa Tenggara	0.587663
West Papua	0.676555
West Pokot	0.44769
West Sulawesi	0.576459
West Sumatra	0.667845
West Sussex	0.863886
West Virginia	0.820334
Western Cape	0.719732
Westminster	0.93701
Wigan	0.805483
Wiltshire	0.859021
Windsor and Maidenhead	0.915543
Wirral	0.818112
Wisconsin	0.873096
Wokingham	0.910822
Wolverhampton	0.810888
Worcestershire	0.842681
Wyoming	0.863143
Yamagata	0.838535
Yamaguchi	0.856636
Yamanashi	0.858174

Yazd	0.713638
Yemen	0.45354
Yogyakarta	0.67683
York	0.88811
YucatÃ¡n	0.654459
Zacatecas	0.636671
Zambales	0.654254
Zambia	0.51023
Zamboanga Del Norte	0.538032
Zamboanga Del Sur	0.630995
Zamboanga Sibugay	0.549421
Zanjan	0.661464
Zimbabwe	0.475577

11. Reference

1. Murray CJL. The Global Burden of Disease Study at 30 years. *Nature medicine* 2022; **28**(10): 2019-26.
2. Global burden of 288 causes of death and life expectancy decomposition in 204 countries and territories and 811 subnational locations, 1990-2021: a systematic analysis for the Global Burden of Disease Study 2021. *Lancet (London, England)* 2024; **403**(10440): 2100-32.
3. Global incidence, prevalence, years lived with disability (YLDs), disability-adjusted life-years (DALYs), and healthy life expectancy (HALE) for 371 diseases and injuries in 204 countries and territories and 811 subnational locations, 1990-2021: a systematic analysis for the Global Burden of Disease Study 2021. *Lancet (London, England)* 2024; **403**(10440): 2133-61.
4. Global age-sex-specific mortality, life expectancy, and population estimates in 204 countries and territories and 811 subnational locations, 1950-2021, and the impact of the COVID-19 pandemic: a comprehensive demographic analysis for the Global Burden of Disease Study 2021. *Lancet (London, England)* 2024; **403**(10440): 1989-2056.
5. Vos T, Flaxman AD, Naghavi M, et al. Years lived with disability (YLDs) for 1160 sequelae of 289 diseases and injuries 1990-2010: a systematic analysis for the Global Burden of Disease Study 2010. *Lancet (London, England)* 2012; **380**(9859): 2163-96.
6. Global, regional, and national burden of Alzheimer's disease and other dementias, 1990-2016: a systematic analysis for the Global Burden of Disease Study 2016. *The Lancet Neurology* 2019; **18**(1): 88-106.
7. Flaxman AD, Vos T, Murray CJL. An Integrative Metaregression Framework for Descriptive Epidemiology. 2014.
8. Vieira RT, Caixeta L, Machado S, et al. Epidemiology of early-onset dementia: a review of the literature. *Clinical practice and epidemiology in mental health : CP & EMH* 2013; **9**: 88-95.
9. Jicha GA, Carr SA. Conceptual evolution in Alzheimer's disease: implications for understanding the clinical phenotype of progressive neurodegenerative disease. *Journal of Alzheimer's disease : JAD* 2010; **19**(1): 253-72.
10. Estimation of the global prevalence of dementia in 2019 and forecasted prevalence in 2050: an analysis for the Global Burden of Disease Study 2019. *The Lancet Public health* 2022; **7**(2): e105-e25.

11. Matthews FE, Arthur A, Barnes LE, et al. A two-decade comparison of prevalence of dementia in individuals aged 65 years and older from three geographical areas of England: results of the Cognitive Function and Ageing Study I and II. *Lancet (London, England)* 2013; **382**(9902): 1405-12.
12. Global, regional, and national comparative risk assessment of 84 behavioural, environmental and occupational, and metabolic risks or clusters of risks for 195 countries and territories, 1990-2017: a systematic analysis for the Global Burden of Disease Study 2017. *Lancet (London, England)* 2018; **392**(10159): 1923-94.
13. Global burden and strength of evidence for 88 risk factors in 204 countries and 811 subnational locations, 1990-2021: a systematic analysis for the Global Burden of Disease Study 2021. *Lancet (London, England)* 2024; **403**(10440): 2162-203.
14. Organization WH. Health Inequality Monitor. *World Health Organization* 2024; **https://**www.who.int/data/inequality-monitor.
15. Organization WH. Handbook on health inequality monitoring: with a special focus on low- and middle-income countries. *World Health Organization* 2013.
16. Hosseinpoor AR, Schlotheuber A, Nambiar D. Health Equity Assessment Toolkit Plus (HEAT Plus): software for exploring and comparing health inequalities using uploaded datasets. 2018; **11**(sup1): 1440783.
17. Hosseinpoor AR, Bergen N, Kirkby K, Schlotheuber A. Strengthening and expanding health inequality monitoring for the advancement of health equity: a review of WHO resources and contributions. *International journal for equity in health* 2023; **22**(1): 49.
18. Riebler A, Held L. Projecting the future burden of cancer: Bayesian age-period-cohort analysis with integrated nested Laplace approximations. *Biometrical journal Biometrische Zeitschrift* 2017; **59**(3): 531-49.
19. Fosse E. Bayesian age–period–cohort models: Age, Period and Cohort Effects; 2020.
20. Thiago, G., Martins, et al. Bayesian computing with INLA: New features. *Computational Statistics & Data Analysis* 2013.
21. Lindgren F, Rue H. Bayesian Spatial and Spatio-temporal Modelling with R-INLA. *Journal of statistical software* 2015; **63**.

Additional Results in Tables and Figures

Table S1. Age standardised mortality of ADOD in people aged ≥65 years and their AAPC at global and regional level, 1990-2021

	No in 1990 (0000s)	Age standardised rate in 1990 (per 100 000)	No in 2021 (0000s)	Age standardised rate in 2021 (per 100 000)	AAPC (95% CI)	P value
Global	63 (15 to 172)	273 (66 to 738)	188 (47 to 497)	274 (69 to 722)	0 (-0.02 to 0.02)	0.957
Sex:						
Female	44 (11 to 119)	301 (74 to 802)	128 (33 to 330)	304 (78 to 782)	0.02 (0 to 0.04)	0.099
Male	18 (4 to 53)	219 (51 to 615)	59 (14 to 167)	225 (54 to 626)	0.08 (0.05 to 0.11)	<0.001
Age group (years):						
65-69	4 (1 to 11)	30 (7 to 86)	8 (2 to 25)	31 (7 to 92)	0.07 (0 to 0.14)	0.037
70-74	4 (1 to 11)	63 (15 to 177)	8 (2 to 25)	64 (16 to 182)	0.03 (-0.04 to 0.11)	0.409
75-79	9 (2 to 26)	144 (34 to 418)	19 (5 to 56)	146 (35 to 423)	0.03 (-0.01 to 0.07)	0.1
80-84	16 (4 to 45)	454 (112 to 1259)	40 (10 to 106)	456 (117 to 1209)	0 (-0.06 to 0.06)	0.927
85-89	16 (4 to 42)	1038 (249 to 2777)	48 (12 to 125)	1043 (262 to 2737)	0.01 (-0.03 to 0.05)	0.523
90-94	9 (2 to 24)	2131 (524 to 5494)	38 (10 to 94)	2126 (541 to 5264)	0 (-0.04 to 0.03)	0.816
≥95	4 (1 to 10)	3930 (960 to 10235)	21 (5 to 53)	3906 (987 to 9744)	-0.03 (-0.08 to 0.02)	0.232
SDI level:						
High	27 (7 to 73)	296 (74 to 786)	71 (18 to 178)	287 (75 to 726)	-0.12 (-0.14 to -0.09)	<0.001
High-middle	16 (4 to 44)	283 (68 to 770)	47 (12 to 128)	288 (71 to 776)	0.05 (0.01 to 0.08)	0.005
Middle	12 (3 to 33)	257 (60 to 708)	47 (11 to 127)	267 (65 to 717)	0.11 (0.07 to 0.15)	<0.001
Low-middle	5 (1 to 15)	192 (44 to 544)	18 (4 to 49)	216 (51 to 598)	0.41 (0.31 to 0.52)	<0.001
Low	2 (0 to 5)	212 (48 to 605)	5 (1 to 15)	239 (56 to 669)	0.41 (0.32 to 0.5)	<0.001

Note: Estimates are for individuals aged over 65 years. AAPCs=average annual percent changes. CI=confidence interval. P value for the significant test of AAPCs. SDI=Socio-demographic Index. ADOD=Alzheimer's Disease and Other Dementias. Numbers in parentheses are 95% uncertainty intervals (Cases and age-standardized rate) and 95% confidence interval (AAPCs).

Table S2. Age standardised DALYs of ADOD in people aged ≥65 years and their AAPC at global and regional level, 1990-2021

	No in 1990 (0000s)	Age standardised rate in 1990 (per 100 000)	No in 2021 (0000s)	Age standardised rate in 2021 (per 100 000)	AAPC (95% CI)	P value
Global	1178 (540 to 2621)	4554 (2055 to 10139)	3256 (1491 to 7115)	4601 (2098 to 10049)	0.02 (0 to 0.04)	0.025
Sex:						
Female	810 (371 to 1780)	5057 (2289 to 11107)	2168 (998 to 4624)	5158 (2378 to 10999)	0.05 (0.03 to 0.07)	<0.001
Male	368 (167 to 847)	3675 (1635 to 8516)	1087 (494 to 2489)	3770 (1692 to 8643)	0.06 (0.04 to 0.07)	<0.001
Age group (years):						
65-69	134 (60 to 303)	1087 (487 to 2451)	313 (141 to 709)	1134 (511 to 2569)	0.12 (0.1 to 0.15)	<0.001
70-74	134 (60 to 303)	1937 (956 to 4174)	313 (141 to 709)	2005 (1004 to 4362)	0.09 (0.07 to 0.11)	<0.001
75-79	222 (109 to 496)	3600 (1767 to 8066)	487 (239 to 1065)	3689 (1814 to 8076)	0.06 (0.04 to 0.08)	<0.001
80-84	292 (132 to 660)	8247 (3728 to 18658)	729 (336 to 1600)	8324 (3836 to 18266)	0.01 (-0.04 to 0.06)	0.714
85-89	220 (99 to 483)	14581 (6550 to 31979)	668 (301 to 1439)	14614 (6592 to 31464)	0 (-0.02 to 0.02)	0.9
90-94	105 (44 to 234)	24479 (10203 to 54522)	434 (183 to 937)	24283 (10224 to 52386)	-0.03 (-0.05 to 0)	0.04
⩾95	41 (16 to 92)	39895 (15644 to 90038)	212 (83 to 468)	38958 (15306 to 85932)	-0.09 (-0.14 to -0.04)	<0.001
SDI level:						
High	476 (221 to 1038)	4895 (2246 to 10668)	1102 (507 to 2325)	4729 (2195 to 9976)	-0.11 (-0.13 to -0.1)	<0.001
High-middle	307 (140 to 689)	4709 (2103 to 10593)	836 (386 to 1826)	4927 (2266 to 10775)	0.1 (0.06 to 0.15)	<0.001
Middle	243 (110 to 549)	4384 (1949 to 9945)	881 (407 to 1941)	4624 (2116 to 10175)	0.15 (0.12 to 0.18)	<0.001
Low-middle	110 (51 to 252)	3368 (1528 to 7712)	330 (146 to 759)	3622 (1586 to 8309)	0.24 (0.19 to 0.3)	<0.001
Low	40 (18 to 92)	3614 (1576 to 8423)	103 (44 to 244)	3869 (1631 to 9156)	0.23 (0.17 to 0.29)	<0.001

Note: Estimates are for individuals aged over 65 years. AAPCs=average annual percent changes. CI=confidence interval. DALYs=disability-adjusted life years. P value for the significant test of AAPCs. SDI=Socio-demographic Index. ADOD=Alzheimer's Disease and Other Dementias. Numbers in parentheses are 95% uncertainty intervals (Cases and age-standardized rate) and 95% confidence interval (AAPCs).

Table S3. Age standardized prevalence of ADOD in people aged ≥65 years and their AAPCs at regional levels, 1990-2021

Regions	No in 1990 (000s)	Age standardised rate in 1990 (per 100 000)	No in 2021 (000s)	Age standardised rate in 2021 (per 100 000)	AAPC (95% CI)	P value
Asia						
Central Asia	211.7 (166.1 to 264.2)	6301 (4940 to 7873)	316.8 (248 to 397.3)	6173 (4833 to 7730)	-0.06 (-0.07 to -0.05)	<0.001
East Asia	3184.8 (2476.3 to 4013.8)	6780 (5280 to 8544)	14947.3 (11636.4 to 18755.7)	8792 (6862 to 11019)	0.79 (0.73 to 0.84)	<0.001
South Asia	1330.2 (1043.8 to 1679.6)	4338 (3400 to 5475)	4145.2 (3239.3 to 5255.4)	4237 (3308 to 5375)	-0.07 (-0.1 to -0.05)	<0.001
Southeast Asia	968.6 (759.5 to 1211.5)	6483 (5082 to 8102)	2632.1 (2053.7 to 3309.1)	6187 (4827 to 7777)	-0.15 (-0.16 to -0.14)	<0.001
High-income Asia Pacific	999.8 (789.8 to 1249.5)	6468 (5098 to 8095)	3845.9 (3026.5 to 4811.4)	6732 (5307 to 8407)	0.16 (0.13 to 0.19)	<0.001
Europe						
Central Europe	736.2 (575.2 to 924.1)	6473 (5054 to 8128)	1390.9 (1088.6 to 1747.2)	6292 (4925 to 7902)	-0.09 (-0.1 to -0.08)	<0.001
Eastern Europe	1388.8 (1083.1 to 1744.7)	6596 (5140 to 8302)	2093.5 (1634 to 2635.9)	6472 (5051 to 8145)	-0.06 (-0.07 to -0.05)	<0.001
Western Europe	3860.6 (3154.9 to 4648.6)	6952 (5676 to 8378)	7260.7 (5785.9 to 8935.6)	6776 (5388 to 8355)	-0.08 (-0.13 to -0.03)	0.003
Africa						
Central Sub-Saharan Africa	73.5 (57.8 to 92.1)	7422 (5835 to 9274)	187.7 (149.1 to 233.3)	7425 (5887 to 9212)	0 (-0.01 to 0.01)	0.667
Eastern Sub-Saharan Africa	232.2 (182.6 to 290.3)	6010 (4714 to 7503)	525.7 (416.5 to 655)	5706 (4514 to 7108)	-0.17 (-0.17 to -0.16)	<0.001
Southern Sub-Saharan Africa	111.3 (87.1 to 140.4)	6228 (4869 to 7855)	212.2 (166 to 267.5)	5880 (4588 to 7416)	-0.19 (-0.21 to -0.16)	<0.001
Western Sub-Saharan Africa	218.4 (170.8 to 274.4)	4207 (3284 to 5297)	424.8 (331.6 to 533.9)	3886 (3029 to 4888)	-0.26 (-0.27 to -0.25)	<0.001
North Africa and Middle East	785.8 (620.5 to 982)	8078 (6378 to 10082)	2190.3 (1717.3 to 2733.1)	7677 (6021 to 9569)	-0.17 (-0.18 to -0.15)	<0.001
North America						

Caribbean	112 (88.8 to 139.1)	5479 (4331 to 6809)	263.3 (207.3 to 328.5)	5425 (4276 to 6767)	-0.06 (-0.08 to -0.03)	<0.001
High-income North America	2697.2 (2133.4 to 3369.2)	7938 (6277 to 9917)	4862.5 (3832.6 to 6051.5)	7455 (5879 to 9272)	-0.2 (-0.22 to -0.17)	<0.001
South America						
Andean Latin America	64.4 (50.5 to 80.6)	4352 (3409 to 5446)	208.9 (163.5 to 261.6)	4288 (3355 to 5370)	-0.05 (-0.06 to -0.04)	<0.001
Central Latin America	346.4 (272.6 to 433.7)	6150 (4834 to 7699)	1187.4 (932.6 to 1489.7)	5845 (4592 to 7330)	-0.16 (-0.18 to -0.15)	<0.001
Southern Latin America	223.1 (175.2 to 278.4)	6138 (4817 to 7661)	485.8 (380 to 609.7)	5870 (4592 to 7369)	-0.14 (-0.16 to -0.12)	<0.001
Tropical Latin America	428.5 (338.5 to 534.1)	7314 (5765 to 9129)	1566.3 (1232.9 to 1960)	7339 (5780 to 9182)	0 (-0.02 to 0.03)	0.758
Oceania						
Oceania	8.5 (6.6 to 10.7)	6647 (5179 to 8345)	22.7 (17.7 to 28.6)	6330 (4942 to 7952)	-0.16 (-0.17 to -0.15)	<0.001
Australasia	142.3 (112.8 to 175.4)	7019 (5562 to 8650)	329.3 (267.1 to 400.2)	5931 (4806 to 7213)	-0.54 (-0.56 to -0.52)	<0.001

Note: Estimates are for individuals aged over 65 years. AAPCs=average annual percent changes. CI=confidence interval. P value for the significant test of AAPCs. SDI=Socio-demographic Index. Numbers in parentheses are 95% uncertainty intervals (Cases and age-standardized rate) and 95% confidence interval (AAPCs).

Table S4. Age standardized prevalence, mortality and DALYs of ADOD in people aged ≥65 years at regional levels by sex, 2021

Regions	Age-standardized rate in 2021 (per 100,000)					
	Male			Female		
	Prevalence	Mortality	DALYs	Prevalence	Mortality	DALYs
Asia						
Central Asia	5440 (4234 to 6837)	195 (45 to 552)	3368 (1544 to 7685)	6569 (5154 to 8207)	230 (55 to 634)	4119 (1933 to 9066)
East Asia	6986 (5371 to 8842)	275 (64 to 794)	4607 (2080 to 10892)	10077 (7894 to 12580)	365 (90 to 956)	6415 (3011 to 13332)
South Asia	3901 (3035 to 4957)	156 (35 to 443)	2628 (1131 to 6142)	4512 (3535 to 5713)	209 (49 to 574)	3473 (1468 to 8115)
Southeast Asia	5164 (4001 to 6522)	195 (45 to 537)	3345 (1486 to 7615)	6887 (5380 to 8630)	276 (66 to 745)	4738 (2141 to 10500)
High-income Asia Pacific	5492 (4300 to 6899)	222 (54 to 597)	3691 (1652 to 8249)	7548 (5969 to 9406)	327 (92 to 757)	5362 (2566 to 10497)
Europe						
Central Europe	5553 (4309 to 7001)	193 (45 to 542)	3358 (1540 to 7570)	6714 (5267 to 8420)	237 (58 to 646)	4231 (1981 to 9121)
Eastern Europe	5618 (4337 to 7111)	195 (45 to 545)	3397 (1553 to 7734)	6831 (5351 to 8578)	241 (57 to 654)	4295 (2011 to 9388)
Western Europe	5808 (4575 to 7207)	232 (56 to 623)	3828 (1738 to 8512)	7443 (5943 to 9162)	312 (83 to 773)	5128 (2404 to 10506)
Africa						
Central Sub-Saharan Africa	5178 (4061 to 6463)	261 (60 to 738)	4107 (1674 to 9862)	8748 (6955 to 10829)	442 (103 to 1192)	7168 (2982 to 16677)
Eastern Sub-Saharan Africa	4307 (3375 to 5404)	208 (48 to 591)	3344 (1373 to 8017)	6768 (5367 to 8397)	356 (85 to 948)	5669 (2389 to 12872)
Southern Sub-Saharan Africa	4474 (3478 to 5673)	174 (39 to 495)	2961 (1292 to 6997)	6603 (5153 to 8322)	279 (66 to 773)	4715 (2081 to 10788)
Western Sub-Saharan Africa	3107 (2409 to 3927)	157 (35 to 466)	2454 (994 to 6128)	4559 (3567 to 5717)	251 (57 to 703)	3883 (1600 to 9252)
North Africa and Middle East	6816 (5330 to 8519)	251 (60 to 686)	4298 (1960 to 9577)	8504 (6688 to 10599)	307 (74 to 824)	5434 (2528 to 11704)
North America						
Caribbean	4828 (3785 to 6055)	160 (37 to 446)	2855 (1331 to 6355)	5888 (4642 to 7315)	179 (42 to 492)	3382 (1653 to 7151)
High-income North America	6445 (5012 to 8094)	270 (66 to 717)	4339 (1928 to 9711)	8225 (6525 to 10207)	334 (87 to 840)	5612 (2595 to 11781)
South America						
Andean Latin America	3694 (2884 to 4648)	135 (31 to 366)	2355 (1065 to 5198)	4773 (3736 to 5971)	164 (38 to 440)	2990 (1418 to 6349)
Central Latin America	5153 (4024 to 6493)	158 (37 to 434)	2899 (1378 to 6328)	6372 (5010 to 7978)	199 (48 to 539)	3724 (1816 to 7831)

Southern Latin America	4956 (3824 to 6296)	167 (39 to 462)	2942 (1368 to 6535)	6448 (5059 to 8068)	248 (62 to 651)	4262 (1976 to 9133)
Tropical Latin America	6969 (5466 to 8753)	253 (62 to 677)	4414 (2013 to 9830)	7593 (5993 to 9497)	320 (84 to 811)	5454 (2486 to 11689)
Oceania						
Oceania	5412 (4185 to 6850)	200 (46 to 570)	3426 (1524 to 7889)	7200 (5612 to 8996)	253 (60 to 702)	4535 (2104 to 9982)
Australasia	4721 (3796 to 5703)	195 (46 to 529)	3203 (1406 to 7149)	6912 (5598 to 8481)	293 (76 to 736)	4860 (2254 to 10148)

Note: Estimates are for individuals aged over 65 years. CI=confidence interval. Numbers in parentheses are 95% uncertainty intervals (Cases and age-standardized rate).

Table S5. Age standardized mortality of ADOD in people aged ≥65 years and their AAPCs at regional levels, 1990-2021

Regions	No in 1990 (000s)	Age standardised rate in 1990 (per 100 000)	No in 2021 (000s)	Age standardised rate in 2021 (per 100 000)	AAPC (95% CI)	P value
Asia						
Central Asia	6.9 (1.6 to 19)	225 (53 to 622)	10.3 (2.4 to 28.8)	218 (52 to 606)	-0.1 (-0.17 to -0.03)	0.008
East Asia	113.2 (26.6 to 311.5)	336 (79 to 913)	486.1 (118.7 to 1326.1)	332 (81 to 896)	-0.07 (-0.15 to 0.02)	0.109
South Asia	37.5 (8.4 to 110.6)	152 (34 to 445)	153.7 (35.9 to 433.5)	186 (43 to 519)	0.69 (0.5 to 0.87)	< 0.001
Southeast Asia	27.4 (6.3 to 77.7)	223 (51 to 622)	91 (21.7 to 249.5)	245 (59 to 663)	0.31 (0.27 to 0.34)	< 0.001
High-income Asia Pacific	40.4 (10.1 to 107.7)	304 (76 to 798)	197.8 (54.1 to 470.7)	291 (79 to 703)	-0.16 (-0.21 to -0.11)	< 0.001
Europe						
Central Europe	21.3 (5 to 60.1)	226 (53 to 630)	48.5 (11.6 to 133.4)	222 (53 to 611)	-0.05 (-0.08 to -0.02)	0.001
Eastern Europe	40.9 (9.6 to 115.8)	234 (55 to 658)	72.7 (17.3 to 200.4)	228 (54 to 629)	-0.08 (-0.12 to -0.04)	< 0.001
Western Europe	149.6 (37.1 to 400.9)	294 (73 to 782)	335 (86.8 to 844.6)	283 (73 to 719)	-0.11 (-0.14 to -0.09)	< 0.001
Africa						
Central Sub-Saharan Africa	2.2 (0.5 to 6.3)	331 (77 to 913)	7.3 (1.7 to 20.6)	380 (90 to 1044)	0.46 (0.41 to 0.51)	< 0.001
Eastern Sub-Saharan Africa	7.4 (1.7 to 21.2)	263 (61 to 733)	21.6 (5.1 to 59.3)	296 (70 to 800)	0.4 (0.35 to 0.44)	< 0.001
Southern Sub-Saharan Africa	3.7 (0.9 to 10.1)	235 (55 to 647)	7.3 (1.7 to 20.7)	247 (58 to 688)	0.17 (0.1 to 0.24)	< 0.001
Western Sub-Saharan Africa	7.9 (1.8 to 22.5)	196 (45 to 554)	18.4 (4.2 to 53.2)	209 (47 to 595)	0.19 (0.16 to 0.23)	< 0.001
North Africa and Middle East	24.6 (5.8 to 68.4)	305 (73 to 838)	69.4 (16.6 to 189.5)	279 (67 to 754)	-0.28 (-0.34 to -0.22)	< 0.001
North America						
Caribbean	3.1 (0.7 to 8.7)	177 (42 to 496)	8.7 (2 to 24)	171 (40 to 472)	-0.11 (-0.16 to -0.05)	< 0.001
High-income North America	104 (26 to 273.7)	317 (79 to 831)	211.4 (53.9 to 539.2)	308 (79 to 790)	-0.09 (-0.13 to -0.06)	< 0.001
South America						
Andean Latin America	2.2 (0.5 to 6)	157 (36 to 437)	7.3 (1.7 to 19.6)	152 (35 to 408)	-0.1 (-0.23 to 0.04)	0.166
Central Latin America	9.1 (2.1 to 25.6)	185 (44 to 516)	36.9 (8.9 to 99.6)	182 (44 to 493)	-0.06 (-0.12 to 0)	0.054
Southern Latin America	7.1 (1.7 to 19.8)	225 (54 to 621)	18.3 (4.5 to 48.5)	219 (54 to 582)	-0.09 (-0.11 to -0.06)	< 0.001
Tropical Latin America	14.8 (3.6 to 40.3)	303 (75 to 810)	62.1 (15.9 to 160.3)	294 (76 to 759)	-0.1 (-0.13 to -0.06)	< 0.001

Oceania						
Oceania	0.2 (0.1 to 0.6)	248 (57 to 704)	0.7 (0.2 to 1.9)	228 (54 to 641)	-0.28 (-0.3 to -0.27)	< 0.001
Australasia	4.8 (1.2 to 13.1)	268 (65 to 717)	14.9 (3.7 to 38.5)	253 (63 to 656)	-0.19 (-0.24 to -0.15)	< 0.001

Note: Estimates are for individuals aged over 65 years. AAPCs=average annual percent changes. CI=confidence interval. P value for the significant test of AAPCs. SDI=Socio-demographic Index. Numbers in parentheses are 95% uncertainty intervals (Cases and age-standardized rate) and 95% confidence interval (AAPCs).

Table S6. Age standardized DALYs of ADOD in people aged ≥65 years and their AAPCs at regional levels, 1990-2021

Regions	No in 1990 (000s)	Age standardised rate in 1990 (per 100 000)	No in 2021 (000s)	Age standardised rate in 2021 (per 100 000)	AAPC (95% CI)	P value
Asia						
Central Asia	128.5 (60.3 to 284.1)	3967 (1839 to 8798)	193.8 (90.3 to 432.6)	3857 (1789 to 8585)	-0.09 (-0.16 to -0.03)	0.005
East Asia	2274.6 (1002.8 to 5143.1)	5377 (2300 to 12260)	9205.1 (4298.6 to 20094.8)	5685 (2619 to 12421)	0.15 (0.03 to 0.26)	0.012
South Asia	796.3 (364.4 to 1857)	2716 (1224 to 6352)	2897.4 (1258.8 to 6809.8)	3091 (1324 to 7248)	0.43 (0.34 to 0.52)	< 0.001
Southeast Asia	563.1 (264 to 1240.6)	3982 (1835 to 8804)	1710 (778 to 3835.5)	4181 (1880 to 9356)	0.16 (0.14 to 0.17)	< 0.001
High-income Asia Pacific	706.7 (318.5 to 1556.6)	4838 (2147 to 10630)	2903.6 (1341.1 to 5895)	4736 (2222 to 9646)	-0.07 (-0.12 to -0.02)	0.009
Europe						
Central Europe	426.2 (201.5 to 951.3)	3984 (1849 to 8914)	862.8 (401.2 to 1883.7)	3920 (1820 to 8563)	-0.06 (-0.07 to -0.04)	< 0.001
Eastern Europe	812.8 (384.8 to 1811.2)	4127 (1914 to 9251)	1303.6 (606.3 to 2876.8)	4036 (1877 to 8897)	-0.07 (-0.1 to -0.04)	< 0.001
Western Europe	2567.4 (1187.6 to 5614.2)	4791 (2193 to 10473)	5151 (2360.7 to 10862.7)	4625 (2141 to 9752)	-0.11 (-0.13 to -0.08)	< 0.001
Africa						
Central Sub-Saharan Africa	48.8 (21.4 to 113.5)	5472 (2335 to 12666)	140.9 (59.6 to 336.6)	6062 (2518 to 14313)	0.33 (0.3 to 0.36)	< 0.001
Eastern Sub-Saharan Africa	155.1 (69.1 to 356.7)	4374 (1899 to 10111)	400.7 (170 to 935.2)	4690 (1953 to 10910)	0.23 (0.2 to 0.25)	< 0.001
Southern Sub-Saharan Africa	70 (32.3 to 155)	4072 (1852 to 9053)	141.1 (63.4 to 325.1)	4142 (1825 to 9543)	0.06 (0.02 to 0.1)	0.003
Western Sub-Saharan Africa	150.1 (65.7 to 348.9)	3146 (1335 to 7403)	327.2 (137.7 to 783.6)	3232 (1325 to 7767)	0.08 (0.03 to 0.12)	< 0.001

North Africa and Middle East	485.6 (225.3 to 1081.4)	5282 (2406 to 11797)	1334.5 (621.7 to 2924.8)	4877 (2246 to 10703)	-0.25 (-0.29 to -0.22)	< 0.001
North America						
Caribbean	62.7 (30.3 to 137)	3237 (1538 to 7131)	155.6 (74.2 to 334.9)	3152 (1511 to 6776)	-0.09 (-0.11 to -0.06)	< 0.001
High-income North America	1787.6 (828.3 to 3880.8)	5334 (2462 to 11575)	3379.4 (1536 to 7263.7)	5076 (2321 to 10911)	-0.17 (-0.19 to -0.15)	< 0.001
South America						
Andean Latin America	40.2 (18.4 to 90.2)	2775 (1263 to 6246)	131.5 (61.3 to 283.8)	2706 (1261 to 5839)	-0.06 (-0.14 to 0.01)	0.102
Central Latin America	186.7 (90.9 to 405.4)	3449 (1662 to 7504)	685.2 (330.7 to 1457.7)	3368 (1626 to 7166)	-0.08 (-0.11 to -0.06)	< 0.001
Southern Latin America	134.5 (63.6 to 296.8)	3890 (1810 to 8619)	312.5 (145 to 675.9)	3767 (1749 to 8149)	-0.1 (-0.11 to -0.09)	< 0.001
Tropical Latin America	285.3 (129 to 641.8)	5127 (2284 to 11470)	1073.6 (489 to 2330.6)	5040 (2295 to 10937)	-0.05 (-0.07 to -0.03)	< 0.001
Oceania						
Oceania	5 (2.3 to 11.1)	4305 (1944 to 9695)	13.5 (6.3 to 30.2)	3996 (1825 to 8919)	-0.24 (-0.26 to -0.23)	< 0.001
Australasia	88.8 (41.9 to 193.1)	4562 (2125 to 9932)	235.6 (106.3 to 503)	4147 (1886 to 8845)	-0.31 (-0.33 to -0.29)	< 0.001

Note: Estimates are for individuals aged over 65 years. AAPCs=average annual percent changes. CI=confidence interval. P value for the significant test of AAPCs. SDI=Socio-demographic Index. Numbers in parentheses are 95% uncertainty intervals (Cases and age-standardized rate) and 95% confidence interval (AAPCs).

Table S7. Age standardized prevalence of ADOD in people aged ≥65 years in 2021 and their AAPCs between 1990-2021 in 204 countries and territories.

Country or territory	No in 1990 (000s)	Age standardised rate in 1990 (per 100 000)	No in 2021 (000s)	Age standardised rate in 2021 (per 100 000)	AAPC (95% CI)	P value
Afghanistan	31040 (24333 to 38775)	7734 (6046 to 9659)	39724 (31201 to 49815)	7583 (5948 to 9499)	-0.07 (-0.07 to -0.06)	<0.001
Albania	8648 (6719 to 10913)	6355 (4936 to 8009)	24131 (18922 to 30175)	6356 (4982 to 7948)	0 (-0.01 to 0.01)	0.68
Algeria	55792 (43913 to 69930)	7792 (6113 to 9755)	176259 (138440 to 220991)	7446 (5835 to 9332)	-0.15 (-0.16 to -0.13)	<0.001
American Samoa	70 (53 to 88)	6279 (4823 to 7936)	184 (141 to 232)	6132 (4705 to 7750)	-0.08 (-0.09 to -0.07)	<0.001
Andorra	298 (232 to 374)	6974 (5427 to 8750)	983 (764 to 1235)	6528 (5079 to 8200)	-0.21 (-0.23 to -0.19)	<0.001
Angola	12205 (9597 to 15311)	7529 (5914 to 9409)	38332 (30243 to 47926)	7293 (5730 to 9105)	-0.1 (-0.11 to -0.1)	<0.001
Antigua and Barbuda	300 (234 to 376)	5506 (4292 to 6914)	418 (326 to 528)	5361 (4172 to 6762)	-0.09 (-0.1 to -0.08)	<0.001
Argentina	156680 (122503 to 196622)	6166 (4817 to 7741)	310432 (241594 to 390467)	5847 (4550 to 7355)	-0.17 (-0.19 to -0.15)	<0.001
Armenia	11683 (9149 to 14552)	6460 (5054 to 8053)	24589 (19245 to 30844)	6399 (5008 to 8025)	-0.03 (-0.04 to -0.02)	<0.001

Australia	117741 (93415 to 145323)	6998 (5553 to 8634)	272981 (222215 to 331694)	5795 (4711 to 7044)	-0.6 (-0.64 to -0.57)	<0.001
Austria	82826 (64668 to 104174)	7097 (5532 to 8941)	133977 (103639 to 168789)	6617 (5117 to 8345)	-0.22 (-0.24 to -0.2)	<0.001
Azerbaijan	21551 (16931 to 26953)	6419 (5032 to 8040)	36105 (28264 to 45616)	6185 (4837 to 7794)	-0.12 (-0.13 to -0.11)	<0.001
Bahamas	612 (477 to 772)	5503 (4284 to 6937)	1519 (1183 to 1915)	5373 (4183 to 6770)	-0.08 (-0.08 to -0.07)	<0.001
Bahrain	528 (411 to 661)	7836 (6109 to 9787)	2497 (1948 to 3150)	7600 (5947 to 9526)	-0.1 (-0.11 to -0.09)	<0.001
Bangladesh	138617 (108906 to 173490)	4510 (3537 to 5648)	428830 (335300 to 538500)	4337 (3385 to 5450)	-0.13 (-0.15 to -0.11)	<0.001
Barbados	1688 (1323 to 2113)	5569 (4354 to 6979)	2508 (1956 to 3159)	5322 (4150 to 6703)	-0.15 (-0.16 to -0.13)	<0.001
Belarus	70748 (55200 to 89047)	6650 (5181 to 8377)	97326 (75978 to 122377)	6620 (5169 to 8310)	-0.01 (-0.02 to 0)	0.019
Belgium	117795 (93798 to 145121)	7995 (6354 to 9867)	185690 (143886 to 233190)	7008 (5429 to 8799)	-0.42 (-0.44 to -0.41)	<0.001
Belize	456 (357 to 571)	5720 (4479 to 7166)	1140 (897 to 1430)	5484 (4318 to 6871)	-0.13 (-0.15 to -0.12)	<0.001
Benin	6418 (5000 to 8092)	4630 (3599 to 5843)	12631 (10036 to 15768)	4099 (3248 to 5125)	-0.39 (-0.41 to -0.37)	<0.001
Bermuda	277 (217 to 350)	5677 (4429 to 7165)	783 (612 to 987)	5571 (4360 to 7024)	-0.06 (-0.07 to -0.05)	<0.001

Bhutan	540 (421 to 684)	4615 (3592 to 5826)	1951 (1520 to 2463)	4222 (3286 to 5329)	-0.29 (-0.29 to -0.28)	<0.001
Bolivia (Plurinational State of)	8898 (6933 to 11178)	4507 (3506 to 5664)	27210 (21202 to 34284)	4423 (3447 to 5571)	-0.06 (-0.07 to -0.05)	<0.001
Bosnia and Herzegovina	15825 (12307 to 19928)	6304 (4901 to 7944)	36400 (28371 to 45627)	6305 (4912 to 7899)	0 (-0.01 to 0.01)	0.676
Botswana	1709 (1342 to 2138)	6106 (4781 to 7637)	4635 (3611 to 5806)	5845 (4552 to 7322)	-0.14 (-0.15 to -0.13)	<0.001
Brazil	416460 (328989 to 519294)	7323 (5771 to 9143)	1536009 (1208736 to 1921991)	7354 (5790 to 9200)	0.01 (-0.02 to 0.03)	0.628
Brunei Darussalam	352 (273 to 444)	5734 (4436 to 7225)	994 (771 to 1252)	5697 (4419 to 7170)	-0.02 (-0.04 to 0.01)	0.177
Bulgaria	55555 (43159 to 69867)	6497 (5035 to 8187)	89972 (70147 to 112862)	6351 (4950 to 7973)	-0.07 (-0.08 to -0.07)	<0.001
Burkina Faso	11360 (8940 to 14233)	4651 (3641 to 5834)	23775 (18500 to 29997)	4330 (3357 to 5460)	-0.23 (-0.24 to -0.22)	<0.001
Burundi	8585 (6747 to 10764)	6200 (4859 to 7776)	13721 (10748 to 17234)	5658 (4417 to 7100)	-0.29 (-0.31 to -0.28)	<0.001
Cabo Verde	977 (770 to 1224)	4499 (3536 to 5637)	1467 (1142 to 1848)	4264 (3321 to 5365)	-0.17 (-0.18 to -0.16)	<0.001
Cambodia	15799 (12339 to 19838)	6612 (5155 to 8301)	45237 (35137 to 56872)	6366 (4938 to 7998)	-0.13 (-0.13 to -0.12)	<0.001
Cameroon	10448 (8181 to 13135)	4314 (3367 to 5424)	27020 (21014 to 34082)	4078 (3162 to 5146)	-0.18 (-0.19 to -0.17)	<0.001

Canada	249924 (204443 to 298246)	8628 (7060 to 10296)	556151 (452448 to 667002)	7533 (6125 to 9035)	-0.44 (-0.49 to -0.39)	<0.001
Central African Republic	3580 (2805 to 4459)	7956 (6244 to 9888)	6496 (5133 to 8119)	7936 (6293 to 9841)	-0.01 (-0.03 to 0.01)	0.349
Chad	8752 (6814 to 10994)	4582 (3562 to 5758)	12998 (10146 to 16323)	4132 (3216 to 5191)	-0.33 (-0.34 to -0.32)	<0.001
Chile	44738 (35527 to 55411)	5999 (4754 to 7438)	139506 (109214 to 174878)	5920 (4634 to 7423)	-0.05 (-0.06 to -0.03)	<0.001
China	3084208 (2396019 to 3890168)	6834 (5319 to 8621)	14579995 (11338402 to 18316060)	8921 (6955 to 11195)	0.81 (0.75 to 0.86)	<0.001
Colombia	75340 (59204 to 93952)	6393 (5020 to 7971)	316635 (247709 to 398423)	6293 (4931 to 7911)	-0.05 (-0.06 to -0.04)	<0.001
Comoros	608 (478 to 763)	5987 (4684 to 7507)	1736 (1364 to 2164)	5668 (4440 to 7067)	-0.17 (-0.18 to -0.17)	<0.001
Congo	3770 (2947 to 4728)	7355 (5747 to 9206)	8663 (7311 to 10200)	6950 (5848 to 8193)	-0.18 (-0.2 to -0.16)	<0.001
Cook Islands	49 (38 to 62)	6308 (4837 to 7947)	138 (107 to 175)	6180 (4769 to 7799)	-0.07 (-0.07 to -0.06)	<0.001
Costa Rica	8820 (6914 to 11041)	6368 (4988 to 7974)	30441 (23698 to 38374)	6209 (4840 to 7821)	-0.08 (-0.09 to -0.07)	<0.001
Coted'Ivoire	7397 (5768 to 9356)	4329 (3366 to 5469)	22934 (17816 to 28905)	4153 (3218 to 5240)	-0.13 (-0.14 to -0.12)	<0.001
Croatia	29158 (22738 to 36806)	6615 (5159 to 8344)	59285 (45958 to 74761)	6353 (4922 to 8017)	-0.13 (-0.15 to -0.11)	<0.001

Cuba	44541 (35454 to 55217)	5106 (4050 to 6338)	98199 (77321 to 121815)	5182 (4081 to 6425)	0 (-0.06 to 0.06)	0.953
Cyprus	3942 (3056 to 4979)	6978 (5416 to 8781)	12425 (9654 to 15621)	6686 (5191 to 8417)	-0.13 (-0.14 to -0.13)	<0.001
Czechia	75686 (58995 to 95155)	6339 (4934 to 7977)	137683 (107328 to 172997)	6268 (4885 to 7877)	-0.04 (-0.05 to -0.03)	<0.001
Democratic People's Republic of Korea	54675 (42049 to 68987)	6280 (4835 to 7908)	149917 (116631 to 188785)	6158 (4781 to 7754)	-0.06 (-0.08 to -0.04)	<0.001
Democratic Republic of the Congo	50507 (39752 to 63236)	7347 (5776 to 9179)	128219 (101174 to 160028)	7483 (5904 to 9325)	0.06 (0.05 to 0.07)	<0.001
Denmark	48737 (38888 to 60243)	5889 (4692 to 7290)	59380 (46709 to 73899)	4701 (3696 to 5852)	-0.73 (-0.77 to -0.7)	<0.001
Djibouti	336 (263 to 420)	6254 (4901 to 7812)	1601 (1260 to 2014)	5885 (4613 to 7398)	-0.19 (-0.2 to -0.19)	<0.001
Dominica	295 (231 to 371)	5607 (4393 to 7056)	357 (278 to 449)	5442 (4238 to 6840)	-0.09 (-0.11 to -0.08)	<0.001
Dominican Republic	14072 (11137 to 17502)	5571 (4396 to 6935)	47278 (37254 to 58664)	5679 (4474 to 7046)	0.02 (-0.02 to 0.06)	0.372
Ecuador	17465 (13659 to 22033)	4519 (3530 to 5703)	59392 (46119 to 74866)	4407 (3417 to 5557)	-0.08 (-0.09 to -0.07)	<0.001
Egypt	96529 (77738 to 118346)	7516 (6023 to 9227)	213223 (168674 to 265137)	7253 (5710 to 9008)	-0.12 (-0.14 to -0.1)	<0.001
El Salvador	14940 (11679 to 18732)	6216 (4862 to 7790)	38669 (30181 to 48583)	6288 (4915 to 7900)	0.04 (0.03 to 0.05)	<0.001

Equatorial Guinea	713 (561 to 890)	7649 (6012 to 9541)	1898 (1493 to 2372)	7258 (5700 to 9061)	-0.17 (-0.17 to -0.16)	<0.001
Eritrea	2220 (1744 to 2789)	6332 (4949 to 7936)	6997 (5470 to 8748)	5898 (4599 to 7380)	-0.23 (-0.24 to -0.22)	<0.001
Estonia	11342 (8866 to 14220)	6516 (5082 to 8176)	19553 (15229 to 24470)	6398 (4979 to 8013)	-0.06 (-0.08 to -0.04)	<0.001
Eswatini	891 (697 to 1119)	5928 (4615 to 7440)	1532 (1203 to 1924)	5670 (4433 to 7135)	-0.14 (-0.15 to -0.14)	<0.001
Ethiopia	53072 (41620 to 66663)	6157 (4810 to 7732)	150969 (118290 to 190744)	5609 (4382 to 7091)	-0.3 (-0.32 to -0.28)	<0.001
Fiji	1071 (822 to 1361)	6390 (4921 to 8083)	2511 (1938 to 3171)	6268 (4855 to 7906)	-0.07 (-0.09 to -0.04)	<0.001
Finland	46168 (36148 to 57230)	6988 (5466 to 8669)	89952 (69755 to 113825)	6220 (4822 to 7874)	-0.37 (-0.4 to -0.35)	<0.001
France	469471 (393740 to 550239)	5570 (4661 to 6555)	867165 (706262 to 1048765)	5271 (4282 to 6381)	-0.18 (-0.19 to -0.17)	<0.001
Gabon	2757 (2157 to 3444)	7452 (5826 to 9289)	4109 (3222 to 5152)	7257 (5681 to 9078)	-0.08 (-0.09 to -0.07)	<0.001
Gambia	869 (682 to 1087)	4610 (3599 to 5767)	2554 (1989 to 3215)	4237 (3291 to 5338)	-0.27 (-0.28 to -0.27)	<0.001
Georgia	30704 (24062 to 38363)	6431 (5032 to 8041)	39015 (30516 to 48775)	6425 (5033 to 8027)	0 (-0.01 to 0.01)	0.78
Germany	999538 (821637 to 1208309)	8193 (6739 to 9905)	1874193 (1506509 to 2273770)	8336 (6679 to 10157)	0.06 (0.04 to 0.07)	<0.001

Ghana	13504 (10509 to 17060)	4166 (3230 to 5270)	36799 (28686 to 46302)	4070 (3162 to 5120)	-0.07 (-0.08 to -0.07)	<0.001
Greece	94594 (73491 to 118852)	7113 (5519 to 8938)	200889 (156570 to 251310)	6791 (5288 to 8511)	-0.15 (-0.16 to -0.14)	<0.001
Greenland	123 (94 to 155)	7921 (6094 to 9972)	304 (235 to 382)	7714 (5981 to 9689)	-0.1 (-0.14 to -0.06)	<0.001
Grenada	437 (341 to 547)	5632 (4404 to 7059)	429 (335 to 538)	5559 (4338 to 6984)	-0.04 (-0.05 to -0.03)	<0.001
Guam	224 (171 to 284)	6182 (4749 to 7818)	1228 (949 to 1552)	6172 (4782 to 7787)	-0.01 (-0.02 to 0.01)	0.461
Guatemala	12161 (9509 to 15304)	6380 (4983 to 8028)	52636 (41311 to 65608)	6306 (4943 to 7868)	-0.04 (-0.05 to -0.02)	<0.001
Guinea	10030 (7885 to 12587)	4511 (3537 to 5663)	15034 (11728 to 18874)	4241 (3305 to 5330)	-0.2 (-0.21 to -0.19)	<0.001
Guinea-Bissau	845 (660 to 1062)	4310 (3346 to 5422)	1326 (1033 to 1679)	4190 (3257 to 5309)	-0.09 (-0.11 to -0.08)	<0.001
Guyana	1357 (1062 to 1698)	5488 (4291 to 6865)	2199 (1714 to 2756)	5416 (4227 to 6780)	-0.04 (-0.05 to -0.03)	<0.001
Haiti	9726 (7621 to 12183)	5803 (4543 to 7267)	20291 (15970 to 25501)	5339 (4202 to 6700)	-0.27 (-0.28 to -0.26)	<0.001
Honduras	8567 (6701 to 10683)	6452 (5036 to 8051)	26230 (20564 to 32774)	6325 (4950 to 7913)	-0.06 (-0.07 to -0.06)	<0.001
Hungary	77988 (60564 to 97765)	6378 (4948 to 8011)	127481 (99317 to 160529)	6283 (4895 to 7912)	-0.05 (-0.07 to -0.02)	<0.001

Iceland	2164 (1721 to 2643)	7737 (6152 to 9451)	4183 (3328 to 5102)	6927 (5514 to 8438)	-0.35 (-0.38 to -0.33)	<0.001
India	1000432 (784549 to 1264963)	4271 (3342 to 5397)	3362517 (2622755 to 4264204)	4225 (3290 to 5360)	-0.03 (-0.05 to 0)	0.029
Indonesia	338401 (262856 to 427710)	6508 (5054 to 8227)	804816 (622444 to 1021109)	6353 (4914 to 8063)	-0.08 (-0.08 to -0.07)	<0.001
Iran (Islamic Republic of)	100573 (78609 to 126897)	7933 (6216 to 9966)	417656 (326100 to 525511)	7626 (5955 to 9594)	-0.13 (-0.14 to -0.12)	<0.001
Iraq	47584 (37108 to 59472)	7947 (6200 to 9929)	100425 (78434 to 125748)	7568 (5908 to 9458)	-0.16 (-0.18 to -0.15)	<0.001
Ireland	25332 (19660 to 31801)	7023 (5446 to 8812)	49447 (38291 to 62056)	6373 (4935 to 8000)	-0.31 (-0.32 to -0.3)	<0.001
Israel	28724 (22341 to 36325)	6839 (5307 to 8646)	80313 (62344 to 101352)	6428 (4991 to 8106)	-0.2 (-0.21 to -0.19)	<0.001
Italy	561194 (439291 to 693240)	6800 (5315 to 8420)	1346656 (1047391 to 1701223)	7675 (5962 to 9705)	0.39 (0.35 to 0.43)	<0.001
Jamaica	10817 (8454 to 13588)	6334 (4949 to 7959)	17831 (13865 to 22354)	5994 (4666 to 7503)	-0.18 (-0.19 to -0.16)	<0.001
Japan	873553 (684777 to 1098521)	6375 (4989 to 8024)	3204219 (2508497 to 4039317)	6638 (5210 to 8359)	0.16 (0.13 to 0.2)	<0.001
Jordan	5440 (4244 to 6838)	7802 (6085 to 9787)	32014 (25167 to 40057)	7808 (6135 to 9769)	0 (-0.07 to 0.08)	0.936
Kazakhstan	56039 (43725 to 70357)	6438 (5017 to 8094)	70800 (55341 to 88621)	6254 (4877 to 7816)	-0.09 (-0.11 to -0.07)	<0.001
Kenya	29244 (22893 to 36830)	5903 (4611 to 7442)	70832 (55581 to 89254)	5851 (4579 to 7374)	-0.03 (-0.05 to -0.01)	0.001

Kiribati	123 (96 to 156)	6938 (5398 to 8739)	223 (174 to 280)	6959 (5425 to 8709)	0.01 (0 to 0.02)	0.001
Kuwait	2360 (1851 to 2936)	8160 (6404 to 10137)	12190 (9537 to 15341)	7640 (5989 to 9588)	-0.21 (-0.23 to -0.19)	<0.001
Kyrgyzstan	13910 (10882 to 17376)	6386 (4988 to 7987)	18567 (14562 to 23104)	6409 (5018 to 7961)	0.02 (0 to 0.03)	0.015
Lao People's Democratic Republic	6963 (5420 to 8740)	6548 (5093 to 8206)	16593 (12982 to 20754)	6343 (4952 to 7933)	-0.11 (-0.11 to -0.1)	<0.001
Latvia	20319 (15805 to 25458)	6537 (5079 to 8202)	29018 (22747 to 36386)	6533 (5116 to 8200)	0 (-0.01 to 0.01)	0.433
Lebanon	11429 (8952 to 14295)	8221 (6435 to 10268)	49025 (38579 to 61034)	8219 (6468 to 10239)	-0.01 (-0.04 to 0.01)	0.301
Lesotho	3488 (2727 to 4375)	6250 (4875 to 7843)	3544 (2785 to 4450)	6153 (4820 to 7732)	-0.05 (-0.06 to -0.04)	<0.001
Liberia	3055 (2382 to 3835)	4205 (3269 to 5290)	4551 (3573 to 5706)	4050 (3175 to 5077)	-0.12 (-0.15 to -0.09)	<0.001
Libya	10470 (8233 to 13058)	8002 (6297 to 9969)	24395 (19082 to 30437)	7584 (5933 to 9460)	-0.17 (-0.18 to -0.16)	<0.001
Lithuania	25501 (20008 to 31909)	6439 (5041 to 8076)	41115 (32115 to 51439)	6404 (5000 to 8017)	0 (-0.04 to 0.04)	0.841
Luxembourg	2746 (2120 to 3458)	5579 (4300 to 7039)	5086 (3979 to 6460)	4745 (3712 to 6029)	-0.53 (-0.61 to -0.44)	<0.001
Madagascar	17038 (13412 to 21401)	5933 (4661 to 7452)	26829 (20962 to 33752)	5619 (4385 to 7058)	-0.17 (-0.19 to -0.16)	<0.001

Malawi	11746 (9186 to 14731)	5928 (4621 to 7433)	23390 (18395 to 29250)	5918 (4636 to 7402)	-0.01 (-0.02 to 0.01)	0.369
Malaysia	43046 (33631 to 54045)	6693 (5226 to 8397)	123023 (95266 to 154832)	6320 (4893 to 7956)	-0.17 (-0.21 to -0.13)	<0.001
Maldives	248 (193 to 312)	6189 (4823 to 7786)	1374 (1074 to 1721)	6406 (5004 to 8024)	0.11 (0.09 to 0.13)	<0.001
Mali	9055 (7073 to 11367)	4460 (3471 to 5595)	20037 (15683 to 25082)	4241 (3310 to 5307)	-0.16 (-0.17 to -0.16)	<0.001
Malta	2438 (1895 to 3065)	7054 (5479 to 8863)	6856 (5332 to 8633)	6510 (5063 to 8197)	-0.26 (-0.28 to -0.24)	<0.001
Marshall Islands	53 (40 to 67)	6081 (4644 to 7702)	80 (61 to 102)	5822 (4488 to 7379)	-0.14 (-0.15 to -0.13)	<0.001
Mauritania	2910 (2273 to 3674)	4558 (3552 to 5750)	5899 (4602 to 7411)	4182 (3259 to 5263)	-0.28 (-0.29 to -0.27)	<0.001
Mauritius	2862 (2220 to 3624)	6418 (4980 to 8115)	9191 (7143 to 11567)	6339 (4933 to 7969)	-0.04 (-0.04 to -0.03)	<0.001
Mexico	164142 (128340 to 207724)	5725 (4465 to 7256)	506554 (394407 to 640932)	5193 (4041 to 6574)	-0.31 (-0.33 to -0.29)	<0.001
Micronesia (Federated States of)	202 (156 to 255)	6838 (5292 to 8607)	232 (180 to 293)	6872 (5330 to 8634)	0.02 (0.01 to 0.02)	<0.001
Monaco	556 (432 to 701)	7048 (5469 to 8898)	722 (556 to 912)	6458 (4967 to 8161)	-0.28 (-0.29 to -0.27)	<0.001
Mongolia	4518 (3549 to 5653)	6485 (5094 to 8119)	7735 (6065 to 9664)	6530 (5110 to 8167)	0.03 (0.01 to 0.05)	0.003
Montenegro	3152 (2461 to 3963)	6476 (5052 to 8146)	4972 (3867 to 6237)	6289 (4890 to 7882)	-0.09 (-0.12 to -0.06)	<0.001

Morocco	79263 (62194 to 98980)	8000 (6272 to 9992)	174994 (136424 to 218882)	7441 (5795 to 9300)	-0.23 (-0.25 to -0.21)	<0.001
Mozambique	19001 (14907 to 23863)	6114 (4772 to 7658)	32455 (25459 to 40591)	5939 (4646 to 7423)	-0.1 (-0.1 to -0.09)	<0.001
Myanmar	88275 (69535 to 110689)	6983 (5491 to 8759)	202527 (158214 to 254613)	6405 (5001 to 8047)	-0.28 (-0.29 to -0.27)	<0.001
Namibia	2002 (1562 to 2510)	6047 (4705 to 7554)	4484 (3511 to 5624)	5802 (4527 to 7282)	-0.13 (-0.14 to -0.13)	<0.001
Nauru	12 (9 to 15)	6200 (4779 to 7827)	18 (14 to 22)	6428 (4972 to 8144)	0.11 (0.1 to 0.12)	<0.001
Nepal	24382 (19093 to 30453)	5004 (3912 to 6243)	67621 (52752 to 84913)	4430 (3446 to 5563)	-0.39 (-0.4 to -0.38)	<0.001
Netherlands	136610 (111488 to 162414)	7228 (5904 to 8593)	259320 (206612 to 317502)	7118 (5670 to 8717)	-0.05 (-0.07 to -0.03)	<0.001
New Zealand	24582 (19096 to 30941)	7126 (5527 to 8974)	56282 (43527 to 71090)	6701 (5183 to 8463)	-0.19 (-0.21 to -0.18)	<0.001
Nicaragua	6816 (5334 to 8524)	6522 (5101 to 8159)	23410 (18342 to 29223)	6462 (5059 to 8070)	-0.03 (-0.03 to -0.03)	<0.001
Niger	5953 (4653 to 7500)	4565 (3558 to 5757)	17982 (14043 to 22580)	4239 (3303 to 5328)	-0.24 (-0.25 to -0.23)	<0.001
Nigeria	108585 (84654 to 137702)	3995 (3104 to 5077)	182064 (141372 to 231380)	3567 (2763 to 4538)	-0.37 (-0.38 to -0.35)	<0.001
Niue	14 (11 to 18)	6240 (4816 to 7908)	11 (8 to 13)	6046 (4627 to 7669)	-0.1 (-0.11 to -0.1)	<0.001

North Macedonia	8246 (6389 to 10407)	6312 (4894 to 7969)	14413 (11149 to 18237)	6256 (4845 to 7900)	-0.03 (-0.04 to -0.02)	<0.001
Northern Mariana Islands	37 (28 to 47)	6189 (4735 to 7833)	156 (119 to 199)	6077 (4648 to 7709)	-0.06 (-0.09 to -0.03)	<0.001
Norway	55814 (43896 to 69843)	7775 (6112 to 9734)	68191 (52724 to 86343)	6398 (4948 to 8097)	-0.62 (-0.65 to -0.59)	<0.001
Oman	2916 (2279 to 3658)	7844 (6132 to 9817)	6579 (5071 to 8292)	7229 (5574 to 9101)	-0.27 (-0.29 to -0.25)	<0.001
Pakistan	166275 (130204 to 210604)	4504 (3518 to 5711)	284267 (221479 to 360647)	4196 (3265 to 5325)	-0.23 (-0.24 to -0.22)	<0.001
Palau	35 (26 to 44)	6025 (4598 to 7638)	66 (50 to 84)	5797 (4419 to 7339)	-0.12 (-0.15 to -0.1)	<0.001
Palestine	4651 (3640 to 5818)	7983 (6246 to 9975)	10775 (8464 to 13501)	7690 (6038 to 9616)	-0.13 (-0.15 to -0.12)	<0.001
Panama	7132 (5550 to 8939)	6187 (4813 to 7752)	24691 (19289 to 31019)	6099 (4770 to 7656)	-0.04 (-0.05 to -0.04)	<0.001
Papua New Guinea	4928 (3834 to 6231)	6817 (5321 to 8571)	14197 (11049 to 17825)	6378 (4969 to 8000)	-0.22 (-0.23 to -0.2)	<0.001
Paraguay	12023 (9428 to 15081)	7115 (5580 to 8921)	30267 (23754 to 37852)	6672 (5241 to 8339)	-0.21 (-0.22 to -0.19)	<0.001
Peru	38008 (29879 to 47557)	4252 (3343 to 5319)	122267 (95479 to 152872)	4205 (3287 to 5254)	-0.04 (-0.05 to -0.03)	<0.001
Philippines	113100 (88569 to 141968)	6634 (5186 to 8350)	322636 (251777 to 406947)	6370 (4969 to 8034)	-0.13 (-0.14 to -0.12)	<0.001

Poland	235029 (182229 to 296207)	6709 (5199 to 8468)	450044 (348260 to 569824)	6305 (4880 to 7978)	-0.2 (-0.21 to -0.19)	<0.001
Portugal	78367 (60887 to 98903)	6793 (5271 to 8570)	190992 (148873 to 239803)	6658 (5189 to 8365)	-0.07 (-0.08 to -0.06)	<0.001
Puerto Rico	17727 (13936 to 22138)	5663 (4446 to 7078)	46753 (36506 to 59059)	5537 (4331 to 6987)	-0.07 (-0.08 to -0.06)	<0.001
Qatar	252 (197 to 317)	7567 (5929 to 9491)	1827 (1413 to 2301)	7351 (5690 to 9244)	-0.09 (-0.11 to -0.07)	<0.001
Republic of Korea	118761 (94766 to 144623)	7493 (5964 to 9142)	602867 (480830 to 738722)	7278 (5802 to 8921)	-0.08 (-0.11 to -0.06)	<0.001
Republic of Moldova	18103 (14174 to 22722)	6266 (4899 to 7876)	34363 (26814 to 43062)	6324 (4936 to 7915)	0.03 (0.02 to 0.04)	<0.001
Romania	125659 (98214 to 158366)	6296 (4910 to 7940)	241386 (187829 to 303857)	6271 (4879 to 7893)	-0.01 (-0.02 to -0.01)	<0.001
Russian Federation	870346 (678415 to 1095582)	6603 (5142 to 8325)	1406324 (1099564 to 1772996)	6495 (5077 to 8183)	-0.05 (-0.06 to -0.04)	<0.001
Rwanda	8423 (6632 to 10554)	6082 (4769 to 7623)	20060 (15765 to 25021)	6020 (4721 to 7513)	-0.03 (-0.04 to -0.02)	<0.001
Saint Kitts and Nevis	179 (139 to 225)	5330 (4140 to 6713)	215 (167 to 270)	5257 (4097 to 6614)	-0.05 (-0.06 to -0.03)	<0.001
Saint Lucia	385 (300 to 482)	5609 (4360 to 7031)	1097 (856 to 1378)	5427 (4233 to 6822)	-0.11 (-0.12 to -0.1)	<0.001
Saint Vincent and the Grenadines	337 (263 to 421)	5751 (4490 to 7197)	625 (489 to 783)	5451 (4257 to 6831)	-0.17 (-0.18 to -0.16)	<0.001

Samoa	324 (250 to 409)	6330 (4897 to 7980)	575 (442 to 728)	6105 (4697 to 7724)	-0.12 (-0.13 to -0.1)	<0.001
San Marino	245 (192 to 305)	7115 (5577 to 8878)	568 (440 to 714)	6295 (4889 to 7927)	-0.39 (-0.42 to -0.37)	<0.001
Sao Tome and Principe	197 (154 to 248)	4308 (3351 to 5415)	259 (201 to 328)	3982 (3092 to 5044)	-0.26 (-0.27 to -0.24)	<0.001
Saudi Arabia	24749 (19249 to 31185)	7429 (5776 to 9354)	48124 (37357 to 61224)	6965 (5408 to 8827)	-0.21 (-0.24 to -0.19)	<0.001
Senegal	8791 (6896 to 11010)	4475 (3506 to 5616)	19969 (15590 to 25207)	4151 (3232 to 5238)	-0.24 (-0.25 to -0.23)	<0.001
Serbia	44441 (34427 to 56071)	6273 (4871 to 7889)	100440 (77859 to 126608)	6276 (4863 to 7910)	0 (-0.01 to 0.01)	0.86
Seychelles	320 (248 to 402)	6455 (4996 to 8104)	503 (389 to 635)	6210 (4804 to 7834)	-0.13 (-0.13 to -0.12)	<0.001
Sierra Leone	6332 (4971 to 7950)	4559 (3563 to 5729)	9700 (7610 to 12234)	4291 (3354 to 5418)	-0.19 (-0.2 to -0.18)	<0.001
Singapore	7127 (5697 to 8649)	5074 (4070 to 6147)	37803 (31019 to 45291)	5285 (4340 to 6318)	0.13 (0.12 to 0.15)	<0.001
Slovakia	31741 (24697 to 39967)	6375 (4958 to 8033)	54120 (42284 to 68082)	6246 (4880 to 7853)	-0.06 (-0.08 to -0.04)	<0.001
Slovenia	13265 (10263 to 16691)	6237 (4823 to 7856)	30297 (23578 to 38017)	6238 (4855 to 7828)	0.01 (-0.01 to 0.02)	0.47
Solomon Islands	367 (284 to 464)	6436 (5006 to 8118)	1090 (846 to 1375)	6423 (4982 to 8099)	-0.01 (-0.01 to 0)	0.027

Somalia	5767 (4547 to 7210)	6103 (4798 to 7633)	14176 (11164 to 17746)	6046 (4745 to 7573)	-0.03 (-0.04 to -0.02)	<0.001
South Africa	89769 (70020 to 113373)	6275 (4890 to 7926)	179027 (139827 to 226114)	5893 (4592 to 7447)	-0.21 (-0.23 to -0.18)	<0.001
South Sudan	9325 (7331 to 11686)	6013 (4708 to 7541)	10553 (8262 to 13271)	5549 (4330 to 6967)	-0.26 (-0.27 to -0.24)	<0.001
Spain	338575 (281694 to 398470)	6846 (5695 to 8064)	664433 (526355 to 823656)	5833 (4625 to 7232)	-0.59 (-0.71 to -0.46)	<0.001
Sri Lanka	41419 (32484 to 51931)	6293 (4930 to 7881)	125301 (97111 to 157895)	6070 (4709 to 7642)	-0.12 (-0.14 to -0.1)	<0.001
Sudan	45085 (35398 to 56037)	7873 (6179 to 9784)	85494 (67271 to 107297)	7272 (5717 to 9126)	-0.26 (-0.27 to -0.24)	<0.001
Suriname	1111 (868 to 1388)	5963 (4664 to 7446)	2797 (2192 to 3510)	5669 (4444 to 7113)	-0.16 (-0.18 to -0.14)	<0.001
Sweden	122628 (97737 to 150583)	7667 (6109 to 9419)	174572 (135680 to 219348)	7029 (5458 to 8843)	-0.27 (-0.3 to -0.24)	<0.001
Switzerland	77232 (60328 to 96398)	7242 (5651 to 9049)	132060 (102476 to 165963)	6468 (5019 to 8134)	-0.35 (-0.42 to -0.29)	<0.001
Syrian Arab Republic	26591 (20864 to 33367)	8162 (6404 to 10223)	58877 (46113 to 74037)	7473 (5841 to 9387)	-0.29 (-0.31 to -0.26)	<0.001
Taiwan (Province of China)	45948 (36001 to 57170)	5026 (3940 to 6232)	217343 (171855 to 265279)	5485 (4340 to 6681)	0.3 (0.24 to 0.35)	<0.001
Tajikistan	12344 (9658 to 15453)	6276 (4908 to 7862)	17259 (13468 to 21833)	5839 (4555 to 7363)	-0.23 (-0.24 to -0.22)	<0.001

Thailand	122366 (97028 to 151875)	5720 (4527 to 7097)	554584 (433478 to 695803)	5806 (4541 to 7282)	0.05 (0.04 to 0.06)	<0.001
Timor-Leste	863 (678 to 1084)	6880 (5413 to 8626)	3669 (2874 to 4595)	6418 (5018 to 8043)	-0.22 (-0.23 to -0.21)	<0.001
Togo	2890 (2271 to 3628)	4409 (3451 to 5538)	7786 (6055 to 9828)	4257 (3302 to 5364)	-0.12 (-0.13 to -0.11)	<0.001
Tokelau	6 (5 to 8)	6272 (4819 to 7930)	8 (6 to 10)	6133 (4735 to 7770)	-0.07 (-0.08 to -0.06)	<0.001
Tonga	225 (175 to 285)	6529 (5064 to 8252)	410 (318 to 515)	6312 (4905 to 7931)	-0.11 (-0.11 to -0.1)	<0.001
Trinidad and Tobago	3609 (2836 to 4528)	5714 (4477 to 7179)	8983 (6999 to 11271)	5569 (4341 to 6982)	-0.08 (-0.09 to -0.08)	<0.001
Tunisia	25633 (20190 to 31920)	8400 (6596 to 10449)	78365 (61473 to 97605)	7849 (6158 to 9775)	-0.21 (-0.24 to -0.19)	<0.001
Turkey	192095 (151419 to 239640)	8691 (6845 to 10837)	590033 (460887 to 737186)	8137 (6355 to 10160)	-0.21 (-0.22 to -0.2)	<0.001
Turkmenistan	7512 (5862 to 9409)	6338 (4940 to 7943)	14869 (11637 to 18619)	6012 (4711 to 7520)	-0.17 (-0.18 to -0.15)	<0.001
Tuvalu	22 (17 to 28)	6582 (5118 to 8312)	42 (33 to 53)	6392 (4939 to 8043)	-0.1 (-0.11 to -0.09)	<0.001
Uganda	21739 (17060 to 27185)	5872 (4593 to 7342)	47198 (37021 to 59051)	5797 (4534 to 7253)	-0.04 (-0.05 to -0.03)	<0.001
Ukraine	372475 (288471 to 473111)	6617 (5118 to 8414)	465754 (360648 to 589736)	6393 (4947 to 8090)	-0.11 (-0.12 to -0.1)	<0.001

United Arab Emirates	1184 (915 to 1496)	7199 (5557 to 9064)	4694 (3597 to 5953)	6447 (4959 to 8123)	-0.36 (-0.38 to -0.34)	<0.001
United Kingdom	561402 (439074 to 703427)	6316 (4936 to 7920)	846216 (659203 to 1061845)	5993 (4664 to 7523)	-0.17 (-0.19 to -0.16)	<0.001
United Republic of Tanzania	36332 (28586 to 45253)	6052 (4743 to 7551)	84662 (68492 to 103674)	5534 (4469 to 6778)	-0.29 (-0.3 to -0.28)	<0.001
United States Virgin Islands	291 (229 to 366)	7874 (6173 to 9889)	934 (724 to 1179)	7446 (5820 to 9330)	-0.1 (-0.11 to -0.1)	<0.001
United States of America	2447102 (1919014 to 3073003)	5518 (4323 to 6929)	4305981 (3363972 to 5399207)	5344 (4141 to 6750)	-0.18 (-0.19 to -0.16)	<0.001
Uruguay	21701 (16948 to 27201)	6214 (4851 to 7786)	35832 (28042 to 45127)	5880 (4603 to 7411)	-0.17 (-0.2 to -0.15)	<0.001
Uzbekistan	53416 (41750 to 67048)	5996 (4681 to 7534)	87846 (68201 to 110479)	5955 (4628 to 7485)	-0.02 (-0.03 to 0)	0.019
Vanuatu	170 (129 to 216)	6062 (4637 to 7677)	506 (389 to 641)	5930 (4560 to 7488)	-0.07 (-0.09 to -0.06)	<0.001
Venezuela (Bolivarian Republic of)	48522 (38241 to 60369)	7218 (5684 to 8979)	168112 (132596 to 209828)	7090 (5597 to 8841)	-0.06 (-0.07 to -0.05)	<0.001
Viet Nam	193528 (151912 to 243316)	6668 (5228 to 8384)	418957 (326098 to 528302)	6276 (4889 to 7912)	-0.2 (-0.21 to -0.19)	<0.001
Yemen	21251 (16770 to 26402)	8568 (6738 to 10623)	61130 (47904 to 76422)	7746 (6058 to 9673)	-0.33 (-0.34 to -0.32)	<0.001
Zambia	8600 (6773 to 10794)	5731 (4497 to 7195)	20055 (15814 to 24940)	5768 (4539 to 7181)	0.02 (0.01 to 0.03)	<0.001

Zimbabwe	13452 (10552 to 16933)	5988 (4684 to 7543)	19018 (14965 to 23849)	5698 (4472 to 7150)	-0.16 (-0.17 to -0.15)	<0.001

Note: Estimates are for individuals aged over 65 years. AAPCs=average annual percent changes. CI=confidence interval. P value for the significant test of AAPCs. Numbers in parentheses are 95% uncertainty intervals (Cases and age-standardized rate) and 95% confidence interval (AAPCs).

Table S8. Age standardized mortality of ADOD in people aged ≥65 years in 2021 and their AAPCs between 1990-2021 in 204 countries and territories.

Country or territory	No in 1990 (000s)	Age standardised rate in 1990 (per 100 000)	No in 2021 (000s)	Age standardised rate in 2021 (per 100 000)	AAPC (95% CI)	P value
Afghanistan	1156 (269 to 3337)	383 (90 to 1075)	1520 (357 to 4157)	361 (86 to 974)	-0.2 (-0.22 to -0.17)	<0.001
Albania	296 (69 to 816)	235 (54 to 643)	758 (171 to 2159)	226 (51 to 639)	-0.14 (-0.25 to -0.03)	0.015
Algeria	1372 (319 to 3906)	305 (70 to 864)	5231 (1205 to 14295)	285 (65 to 779)	-0.22 (-0.26 to -0.18)	<0.001
American Samoa	2 (1 to 6)	262 (60 to 723)	6 (1 to 17)	244 (56 to 671)	-0.22 (-0.26 to -0.18)	<0.001
Andorra	9 (2 to 24)	280 (68 to 725)	45 (11 to 119)	265 (66 to 703)	-0.2 (-0.29 to -0.11)	<0.001
Angola	357 (81 to 1027)	307 (69 to 867)	1402 (326 to 4023)	364 (86 to 1020)	0.56 (0.5 to 0.62)	<0.001
Antigua and Barbuda	10 (2 to 27)	181 (42 to 510)	12 (3 to 33)	171 (40 to 485)	-0.16 (-0.24 to -0.07)	<0.001
Argentina	5010 (1196 to 13944)	228 (55 to 629)	11696 (2872 to 31103)	221 (54 to 587)	-0.1 (-0.14 to -0.07)	<0.001
Armenia	362 (85 to 1010)	218 (51 to 604)	868 (208 to 2331)	233 (56 to 624)	0.21 (0.09 to 0.32)	<0.001
Australia	3970 (965 to 10725)	265 (64 to 710)	12578 (3142 to 32365)	250 (62 to 646)	-0.19 (-0.24 to -0.14)	<0.001
Austria	2940 (703 to 8118)	279 (66 to 765)	5981 (1512 to 15389)	268 (68 to 695)	-0.11 (-0.14 to -0.09)	<0.001
Azerbaijan	678 (160 to 1879)	231 (54 to 638)	1186 (278 to 3391)	224 (53 to 634)	-0.1 (-0.18 to -0.02)	0.011
Bahamas	17 (4 to 49)	175 (41 to 489)	45 (10 to 127)	171 (39 to 480)	-0.08 (-0.17 to 0)	0.053

Bahrain	13 (3 to 38)	320 (75 to 907)	61 (14 to 175)	280 (64 to 796)	-0.41 (-0.51 to -0.32)	<0.001
Bangladesh	4419 (1018 to 12632)	166 (38 to 472)	15477 (3492 to 45981)	186 (42 to 552)	0.42 (0.25 to 0.6)	<0.001
Barbados	45 (10 to 130)	171 (39 to 491)	78 (18 to 217)	173 (39 to 478)	0.0 (-0.11 to 0.17)	0.663
Belarus	2231 (521 to 6247)	229 (54 to 639)	3295 (770 to 9226)	224 (52 to 626)	-0.08 (-0.15 to 0)	0.047
Belgium	4326 (1062 to 11461)	321 (79 to 845)	8702 (2287 to 21651)	291 (76 to 731)	-0.31 (-0.38 to -0.25)	<0.001
Belize	15 (3 to 39)	178 (42 to 481)	36 (8 to 97)	175 (41 to 472)	-0.08 (-0.19 to 0.03)	0.165
Benin	229 (52 to 651)	194 (44 to 551)	509 (116 to 1446)	200 (46 to 563)	0.09 (0.02 to 0.16)	0.011
Bermuda	8 (2 to 22)	185 (43 to 518)	27 (6 to 70)	178 (43 to 474)	-0.06 (-0.37 to 0.26)	0.725
Bhutan	16 (3 to 45)	167 (35 to 467)	90 (21 to 257)	213 (49 to 602)	0.78 (0.74 to 0.82)	<0.001
Bolivia (Plurinational State of)	265 (60 to 764)	167 (38 to 477)	865 (198 to 2385)	169 (39 to 461)	0.04 (0.02 to 0.06)	<0.001
Bosnia and Herzegovina	467 (110 to 1308)	221 (52 to 609)	1160 (268 to 3105)	213 (49 to 564)	-0.12 (-0.19 to -0.04)	0.002
Botswana	49 (11 to 145)	250 (55 to 736)	158 (37 to 443)	245 (57 to 673)	-0.05 (-0.2 to 0.11)	0.541
Brazil	14348 (3541 to 39124)	305 (76 to 814)	60833 (15626 to 157040)	295 (76 to 761)	-0.11 (-0.14 to -0.07)	<0.001
Brunei Darussalam	12 (3 to 33)	245 (57 to 682)	31 (7 to 83)	254 (61 to 681)	0.1 (0.03 to 0.17)	0.007
Bulgaria	1249 (287 to 3612)	217 (49 to 623)	2710 (636 to 7661)	218 (51 to 618)	0 (-0.02 to 0.03)	0.807
Burkina Faso	422 (97 to 1187)	241 (55 to 671)	947 (215 to 2689)	220 (51 to 616)	-0.28 (-0.44 to -0.12)	0.001
Burundi	249 (56 to 707)	246 (55 to 684)	513 (116 to 1485)	273 (62 to 775)	0.34 (0.26 to 0.43)	<0.001
Cabo Verde	37 (8 to 101)	179 (41 to 494)	69 (16 to 188)	190 (44 to 518)	0.2 (0.13 to 0.27)	<0.001

Cambodia	414 (95 to 1169)	228 (52 to 638)	1523 (350 to 4341)	278 (64 to 781)	0.65 (0.61 to 0.69)	<0.001
Cameroon	424 (98 to 1189)	222 (51 to 618)	1069 (235 to 3202)	209 (46 to 617)	-0.22 (-0.29 to -0.15)	<0.001
Canada	6469 (1589 to 17395)	240 (59 to 643)	18211 (4593 to 47025)	231 (58 to 598)	-0.11 (-0.18 to -0.03)	0.005
Central African Republic	112 (25 to 305)	358 (83 to 958)	193 (43 to 533)	349 (77 to 944)	-0.08 (-0.17 to 0)	0.047
Chad	281 (62 to 798)	186 (41 to 525)	470 (104 to 1333)	192 (42 to 537)	0.09 (0.07 to 0.12)	<0.001
Chile	1378 (333 to 3769)	213 (51 to 578)	5031 (1241 to 13221)	212 (52 to 557)	-0.01 (-0.08 to 0.06)	0.876
China	109829 (25861 to 302122)	342 (81 to 928)	470807 (115221 to 1284158)	336 (82 to 909)	-0.08 (-0.17 to 0.01)	0.066
Colombia	2091 (501 to 5834)	194 (47 to 538)	10606 (2637 to 27611)	193 (47 to 508)	-0.04 (-0.07 to 0)	0.027
Comoros	20 (5 to 54)	272 (63 to 724)	77 (18 to 216)	306 (72 to 843)	0.37 (0.34 to 0.41)	<0.001
Congo	121 (28 to 334)	381 (89 to 1031)	341 (79 to 933)	376 (87 to 1005)	-0.04 (-0.07 to -0.01)	0.007
Cook Islands	2 (0 to 5)	274 (64 to 763)	5 (1 to 13)	238 (56 to 641)	-0.46 (-0.5 to -0.41)	<0.001
Costa Rica	265 (63 to 726)	199 (48 to 545)	1036 (257 to 2687)	195 (48 to 511)	-0.06 (-0.08 to -0.04)	<0.001
Coted'Ivoire	259 (60 to 721)	211 (49 to 576)	893 (197 to 2580)	207 (46 to 593)	-0.06 (-0.1 to -0.03)	<0.001
Croatia	870 (202 to 2461)	227 (53 to 638)	1998 (468 to 5493)	221 (52 to 609)	-0.08 (-0.2 to 0.04)	0.186
Cuba	1263 (299 to 3557)	171 (40 to 479)	3371 (783 to 9525)	166 (39 to 471)	-0.09 (-0.17 to -0.01)	0.026
Cyprus	117 (27 to 335)	317 (72 to 898)	428 (102 to 1185)	284 (66 to 790)	-0.36 (-0.44 to -0.28)	<0.001
Czechia	2292 (537 to 6494)	224 (53 to 628)	4785 (1157 to 12970)	221 (54 to 600)	-0.02 (-0.05 to 0)	0.086
Democratic People's Republic of Korea	1814 (397 to 5111)	254 (55 to 711)	5510 (1249 to 15347)	263 (60 to 726)	0.12 (0.07 to 0.16)	<0.001

Democratic Republic of the Congo	1500 (341 to 4332)	328 (75 to 915)	5131 (1164 to 14346)	386 (89 to 1054)	0.54 (0.49 to 0.58)	<0.001
Denmark	2007 (480 to 5543)	254 (61 to 701)	3455 (850 to 9016)	265 (65 to 692)	0.16 (0.05 to 0.26)	0.004
Djibouti	11 (3 to 32)	285 (65 to 801)	60 (13 to 168)	313 (71 to 855)	0.31 (0.28 to 0.33)	<0.001
Dominica	8 (2 to 23)	180 (42 to 518)	11 (3 to 31)	178 (42 to 504)	-0.04 (-0.08 to 0.01)	0.102
Dominican Republic	384 (88 to 1068)	185 (42 to 513)	1458 (334 to 3989)	177 (41 to 485)	-0.14 (-0.25 to -0.02)	0.021
Ecuador	536 (125 to 1514)	157 (36 to 443)	1852 (407 to 5200)	150 (33 to 423)	-0.14 (-0.23 to -0.04)	0.005
Egypt	2781 (638 to 8005)	295 (67 to 839)	5838 (1343 to 16603)	266 (62 to 740)	-0.33 (-0.48 to -0.18)	<0.001
El Salvador	497 (119 to 1353)	202 (48 to 552)	1365 (335 to 3490)	198 (48 to 511)	-0.05 (-0.22 to 0.13)	0.581
Equatorial Guinea	22 (5 to 62)	322 (73 to 913)	73 (16 to 205)	360 (82 to 991)	0.36 (0.3 to 0.42)	<0.001
Eritrea	61 (14 to 176)	261 (60 to 740)	269 (61 to 768)	327 (74 to 906)	0.74 (0.72 to 0.77)	<0.001
Estonia	342 (79 to 973)	226 (52 to 640)	771 (185 to 2058)	240 (57 to 643)	0.18 (0.12 to 0.24)	<0.001
Eswatini	30 (7 to 84)	254 (57 to 723)	49 (11 to 139)	253 (57 to 700)	0.01 (-0.07 to 0.09)	0.788
Ethiopia	1667 (386 to 4820)	291 (68 to 815)	6939 (1662 to 18730)	311 (75 to 826)	0.22 (0.18 to 0.25)	<0.001
Fiji	32 (7 to 93)	255 (58 to 732)	69 (16 to 190)	245 (56 to 676)	-0.14 (-0.21 to -0.07)	<0.001
Finland	1856 (454 to 4943)	309 (76 to 818)	4532 (1155 to 11454)	287 (73 to 729)	-0.26 (-0.29 to -0.22)	<0.001
France	20045 (4854 to 54095)	252 (61 to 678)	45987 (11595 to 117057)	238 (60 to 609)	-0.18 (-0.21 to -0.14)	<0.001
Gabon	113 (27 to 297)	386 (93 to 996)	173 (41 to 475)	387 (91 to 1043)	0.02 (-0.04 to 0.08)	0.518
Gambia	29 (7 to 83)	215 (48 to 602)	107 (24 to 312)	218 (49 to 630)	0.03 (0 to 0.07)	0.065

Georgia	959 (226 to 2687)	224 (53 to 625)	1523 (375 to 4024)	230 (56 to 617)	0.06 (-0.15 to 0.28)	0.568
Germany	36658 (9254 to 95801)	325 (83 to 841)	77550 (20543 to 192281)	322 (85 to 803)	-0.01 (-0.1 to 0.09)	0.909
Ghana	439 (100 to 1255)	182 (41 to 514)	1457 (328 to 4283)	210 (48 to 612)	0.48 (0.43 to 0.52)	<0.001
Greece	3115 (749 to 8636)	273 (65 to 751)	8820 (2174 to 23120)	266 (65 to 701)	-0.08 (-0.1 to -0.07)	<0.001
Greenland	3 (1 to 9)	289 (66 to 818)	9 (2 to 23)	281 (67 to 734)	-0.09 (-0.22 to 0.04)	0.164
Grenada	16 (4 to 43)	192 (45 to 527)	12 (3 to 33)	181 (42 to 513)	-0.04 (-0.19 to 0.11)	0.616
Guam	6 (2 to 18)	251 (59 to 694)	46 (13 to 105)	197 (54 to 470)	-0.81 (-0.96 to -0.67)	<0.001
Guatemala	294 (67 to 841)	204 (46 to 583)	1447 (337 to 3983)	198 (46 to 545)	-0.12 (-0.15 to -0.09)	<0.001
Guinea	337 (76 to 947)	184 (41 to 518)	607 (133 to 1721)	200 (44 to 563)	0.26 (0.23 to 0.3)	<0.001
Guinea-Bissau	28 (6 to 78)	204 (46 to 579)	47 (10 to 139)	213 (47 to 626)	0.12 (0.08 to 0.17)	<0.001
Guyana	35 (8 to 98)	165 (38 to 466)	59 (13 to 165)	168 (38 to 471)	0.03 (-0.2 to 0.26)	0.796
Haiti	228 (52 to 660)	185 (42 to 533)	526 (113 to 1542)	178 (38 to 516)	-0.12 (-0.14 to -0.11)	<0.001
Honduras	243 (56 to 682)	219 (50 to 609)	840 (195 to 2388)	247 (57 to 696)	0.4 (0.3 to 0.5)	<0.001
Hungary	2257 (525 to 6351)	226 (52 to 631)	4457 (1059 to 12118)	219 (52 to 595)	-0.1 (-0.14 to -0.06)	<0.001
Iceland	77 (19 to 204)	271 (66 to 717)	180 (46 to 450)	267 (68 to 674)	-0.05 (-0.18 to 0.07)	0.403
India	26877 (5959 to 79606)	144 (32 to 421)	123910 (29015 to 345263)	183 (43 to 506)	0.84 (0.61 to 1.07)	<0.001
Indonesia	8019 (1764 to 22974)	189 (41 to 535)	23609 (5464 to 68441)	249 (58 to 715)	0.91 (0.85 to 0.98)	<0.001
Iran (Islamic Republic of)	3106 (746 to 8558)	297 (72 to 808)	14180 (3427 to 38120)	274 (66 to 733)	-0.26 (-0.29 to -0.23)	<0.001

Iraq	1702 (415 to 4583)	285 (69 to 767)	3081 (716 to 8459)	281 (66 to 768)	-0.03 (-0.07 to 0.01)	0.096
Ireland	837 (199 to 2331)	278 (66 to 768)	2075 (524 to 5271)	265 (67 to 673)	-0.17 (-0.24 to -0.1)	<0.001
Israel	920 (221 to 2550)	268 (64 to 736)	3549 (901 to 9007)	267 (68 to 680)	-0.02 (-0.1 to 0.05)	0.539
Italy	24933 (6184 to 66230)	346 (86 to 909)	63984 (16933 to 158644)	327 (86 to 818)	-0.18 (-0.23 to -0.12)	<0.001
Jamaica	295 (70 to 810)	178 (42 to 487)	626 (151 to 1676)	173 (41 to 474)	-0.05 (-0.12 to 0.02)	0.129
Japan	35587 (8914 to 94312)	298 (75 to 777)	171325 (47160 to 405587)	288 (79 to 695)	-0.13 (-0.17 to -0.09)	<0.001
Jordan	172 (42 to 466)	286 (70 to 772)	844 (199 to 2382)	255 (61 to 709)	-0.38 (-0.48 to -0.27)	<0.001
Kazakhstan	1786 (422 to 4971)	227 (54 to 627)	1990 (456 to 5752)	212 (48 to 608)	-0.23 (-0.3 to -0.15)	<0.001
Kenya	1032 (235 to 2867)	260 (60 to 714)	2900 (658 to 7625)	313 (72 to 803)	0.6 (0.55 to 0.64)	<0.001
Kiribati	3 (1 to 10)	257 (55 to 729)	7 (2 to 20)	298 (66 to 830)	0.48 (0.46 to 0.49)	<0.001
Kuwait	78 (19 to 206)	294 (74 to 771)	446 (112 to 1174)	266 (67 to 704)	-0.31 (-0.65 to 0.03)	0.073
Kyrgyzstan	449 (105 to 1239)	232 (54 to 636)	587 (141 to 1595)	218 (52 to 592)	-0.19 (-0.26 to -0.12)	<0.001
Lao People's Democratic Republic	154 (34 to 449)	211 (47 to 605)	498 (112 to 1463)	233 (53 to 678)	0.31 (0.28 to 0.35)	<0.001
Latvia	633 (147 to 1792)	227 (53 to 639)	1031 (244 to 2842)	227 (54 to 626)	0 (-0.02 to 0.03)	0.702
Lebanon	325 (76 to 906)	270 (63 to 746)	1616 (393 to 4367)	260 (63 to 703)	-0.12 (-0.22 to -0.01)	0.029
Lesotho	112 (25 to 308)	241 (53 to 660)	109 (25 to 319)	254 (59 to 724)	0.21 (0.16 to 0.27)	<0.001
Liberia	102 (23 to 301)	193 (44 to 556)	181 (39 to 529)	193 (42 to 557)	-0.01 (-0.16 to 0.13)	0.836
Libya	444 (109 to 1135)	335 (82 to 860)	934 (219 to 2548)	306 (72 to 832)	-0.31 (-0.41 to -0.2)	<0.001

Lithuania	826 (194 to 2355)	226 (53 to 642)	1512 (359 to 4189)	229 (54 to 637)	0.05 (-0.02 to 0.11)	0.144
Luxembourg	81 (19 to 230)	193 (44 to 542)	228 (56 to 596)	194 (47 to 510)	0.04 (-0.06 to 0.14)	0.445
Madagascar	470 (108 to 1356)	205 (47 to 586)	758 (171 to 2146)	215 (49 to 597)	0.16 (0.11 to 0.21)	<0.001
Malawi	370 (85 to 1059)	259 (59 to 726)	884 (200 to 2510)	295 (67 to 825)	0.44 (0.36 to 0.51)	<0.001
Malaysia	1618 (401 to 4303)	255 (63 to 679)	4390 (1050 to 12196)	263 (63 to 725)	0.08 (-0.05 to 0.2)	0.236
Maldives	5 (1 to 16)	190 (43 to 541)	44 (11 to 121)	211 (51 to 584)	0.32 (0.29 to 0.34)	<0.001
Mali	345 (79 to 965)	239 (54 to 666)	817 (185 to 2347)	237 (54 to 665)	-0.01 (-0.05 to 0.03)	0.614
Malta	80 (19 to 221)	271 (65 to 736)	287 (74 to 724)	263 (68 to 667)	-0.09 (-0.22 to 0.05)	0.209
Marshall Islands	2 (0 to 5)	281 (64 to 796)	2 (1 to 7)	262 (59 to 746)	-0.22 (-0.24 to -0.21)	<0.001
Mauritania	99 (23 to 278)	204 (46 to 571)	247 (54 to 730)	206 (45 to 604)	0.03 (-0.02 to 0.08)	0.226
Mauritius	85 (20 to 237)	237 (56 to 655)	306 (74 to 836)	219 (53 to 599)	-0.29 (-0.42 to -0.16)	<0.001
Mexico	3908 (911 to 11180)	163 (38 to 464)	14691 (3464 to 42021)	160 (38 to 456)	-0.07 (-0.17 to 0.03)	0.155
Micronesia (Federated States of)	7 (1 to 19)	287 (66 to 815)	8 (2 to 22)	285 (65 to 808)	-0.03 (-0.05 to -0.01)	0.015
Monaco	22 (5 to 61)	284 (66 to 781)	38 (9 to 93)	300 (75 to 747)	0.19 (0.17 to 0.21)	<0.001
Mongolia	139 (32 to 387)	247 (58 to 681)	227 (53 to 640)	237 (56 to 653)	-0.19 (-0.37 to -0.01)	0.041
Montenegro	103 (24 to 282)	224 (52 to 612)	145 (33 to 403)	223 (51 to 616)	0.01 (-0.11 to 0.12)	0.911
Morocco	2477 (586 to 6853)	288 (68 to 790)	5713 (1338 to 15861)	284 (66 to 785)	-0.04 (-0.06 to -0.03)	<0.001
Mozambique	664 (156 to 1867)	293 (69 to 812)	1319 (299 to 3847)	325 (74 to 931)	0.37 (0.29 to 0.45)	<0.001

Myanmar	1954 (446 to 5692)	205 (46 to 590)	6342 (1445 to 18298)	232 (53 to 667)	0.41<br>(0.39 to 0.43)	<0.001
Namibia	57 (13 to 161)	236 (54 to 655)	161 (36 to 468)	263 (59 to 759)	0.37<br>(0.34 to 0.4)	<0.001
Nauru	0 (0 to 1)	273 (62 to 787)	1 (0 to 2)	273 (61 to 791)	0<br>(-0.02 to 0.02)	0.988
Nepal	595 (132 to 1720)	151 (33 to 435)	2323 (519 to 6691)	188 (42 to 537)	0.7<br>(0.66 to 0.75)	<0.001
Netherlands	5567 (1401 to 14485)	313 (79 to 811)	11466 (2930 to 29338)	308 (79 to 788)	-0.05<br>(-0.09 to -0.01)	0.023
New Zealand	875 (215 to 2350)	286 (70 to 760)	2348 (596 to 6103)	271 (69 to 707)	-0.18<br>(-0.23 to -0.13)	<0.001
Nicaragua	191 (46 to 518)	198 (48 to 532)	629 (154 to 1679)	190 (46 to 506)	-0.15<br>(-0.2 to -0.1)	<0.001
Niger	190 (43 to 537)	205 (46 to 571)	651 (145 to 1891)	210 (47 to 595)	0.08<br>(0.02 to 0.13)	0.004
Nigeria	4034 (945 to 11487)	189 (44 to 533)	8793 (2007 to 25124)	206 (47 to 583)	0.27<br>(0.16 to 0.38)	<0.001
Niue	1 (0 to 2)	301 (69 to 841)	0 (0 to 1)	271 (65 to 736)	-0.34<br>(-0.37 to -0.31)	<0.001
North Macedonia	262 (62 to 718)	208 (49 to 570)	349 (81 to 995)	214 (49 to 607)	0.08<br>(0.06 to 0.1)	<0.001
Northern Mariana Islands	1 (0 to 3)	253 (58 to 707)	5 (1 to 14)	248 (59 to 684)	-0.06<br>(-0.12 to 0)	0.066
Norway	2045 (500 to 5474)	291 (71 to 778)	3191 (798 to 8227)	273 (68 to 708)	-0.2<br>(-0.24 to -0.17)	<0.001
Oman	95 (22 to 260)	308 (73 to 821)	205 (51 to 581)	285 (71 to 785)	-0.27<br>(-0.37 to -0.17)	<0.001
Pakistan	5640 (1244 to 16269)	185 (40 to 533)	11932 (2716 to 33250)	213 (48 to 588)	0.44<br>(0.39 to 0.49)	<0.001
Palau	1 (0 to 3)	268 (60 to 755)	2 (0 to 6)	235 (53 to 684)	-0.42<br>(-0.48 to -0.36)	<0.001
Palestine	144 (33 to 415)	291 (66 to 837)	305 (71 to 873)	269 (62 to 758)	-0.25<br>(-0.29 to -0.22)	<0.001
Panama	209 (51 to 573)	189 (46 to 516)	827 (205 to 2125)	190 (47 to 491)	0.03<br>(-0.05 to 0.1)	0.524

Papua New Guinea	114 (25 to 322)	229 (51 to 647)	382 (86 to 1125)	222 (50 to 653)	-0.1 (-0.12 to -0.09)	<0.001
Paraguay	438 (106 to 1178)	270 (65 to 724)	1266 (307 to 3282)	277 (67 to 721)	0.11 (0.01 to 0.22)	0.034
Peru	1358 (314 to 3836)	155 (36 to 438)	4578 (1076 to 12198)	150 (35 to 399)	-0.11 (-0.31 to 0.1)	0.308
Philippines	2692 (624 to 7752)	216 (50 to 618)	10188 (2404 to 28038)	232 (55 to 634)	0.23 (0.14 to 0.33)	<0.001
Poland	7047 (1670 to 19811)	233 (55 to 649)	16691 (4058 to 45019)	229 (56 to 620)	-0.07 (-0.11 to -0.02)	0.003
Portugal	2627 (618 to 7332)	282 (66 to 780)	8441 (2116 to 21812)	272 (68 to 706)	-0.11 (-0.16 to -0.06)	<0.001
Puerto Rico	488 (114 to 1385)	183 (43 to 515)	1716 (423 to 4484)	172 (42 to 457)	-0.21 (-0.27 to -0.16)	<0.001
Qatar	8 (2 to 23)	328 (76 to 918)	52 (12 to 150)	291 (69 to 815)	-0.42 (-0.53 to -0.3)	<0.001
Republic of Korea	4608 (1133 to 12491)	392 (96 to 1046)	25004 (6519 to 61206)	318 (83 to 776)	-0.69 (-0.74 to -0.64)	<0.001
Republic of Moldova	504 (118 to 1437)	226 (53 to 640)	1153 (276 to 3072)	206 (49 to 554)	-0.29 (-0.34 to -0.25)	<0.001
Romania	3358 (784 to 9655)	219 (51 to 627)	8321 (1948 to 23229)	218 (51 to 607)	-0.03 (-0.08 to 0.01)	0.169
Russian Federation	25360 (5941 to 71752)	236 (55 to 660)	49111 (11670 to 134526)	229 (54 to 627)	-0.09 (-0.13 to -0.04)	<0.001
Rwanda	264 (61 to 765)	272 (63 to 775)	816 (190 to 2321)	320 (76 to 896)	0.53 (0.46 to 0.59)	<0.001
Saint Kitts and Nevis	4 (1 to 13)	175 (39 to 511)	6 (1 to 17)	168 (38 to 470)	-0.15 (-0.41 to 0.11)	0.257
Saint Lucia	10 (2 to 28)	180 (41 to 520)	35 (8 to 96)	178 (41 to 493)	-0.04 (-0.2 to 0.13)	0.668
Saint Vincent and the Grenadines	9 (2 to 26)	192 (44 to 542)	17 (4 to 48)	171 (39 to 482)	-0.4 (-0.65 to -0.14)	0.002
Samoa	13 (3 to 36)	301 (69 to 821)	23 (5 to 67)	287 (67 to 809)	-0.15 (-0.17 to -0.13)	<0.001
San Marino	9 (2 to 23)	260 (64 to 682)	27 (7 to 65)	236 (61 to 593)	-0.34 (-0.52 to -0.15)	<0.001

Sao Tome and Principe	8 (2 to 22)	193 (44 to 546)	10 (2 to 30)	191 (45 to 541)	-0.04 (-0.11 to 0.03)	0.289
Saudi Arabia	877 (204 to 2441)	311 (73 to 854)	1493 (351 to 4241)	282 (66 to 789)	-0.33 (-0.36 to -0.29)	<0.001
Senegal	317 (71 to 900)	205 (46 to 576)	900 (197 to 2590)	226 (49 to 644)	0.31 (0.17 to 0.44)	<0.001
Serbia	1322 (301 to 3820)	221 (50 to 638)	3372 (778 to 9490)	216 (50 to 608)	-0.07 (-0.11 to -0.03)	<0.001
Seychelles	11 (3 to 32)	238 (56 to 662)	17 (4 to 46)	225 (52 to 620)	-0.17 (-0.21 to -0.12)	<0.001
Sierra Leone	202 (45 to 586)	184 (41 to 532)	334 (76 to 980)	185 (42 to 537)	0.03 (-0.03 to 0.08)	0.377
Singapore	237 (57 to 645)	205 (49 to 550)	1411 (363 to 3583)	196 (50 to 498)	-0.16 (-0.27 to -0.06)	0.002
Slovakia	992 (231 to 2769)	227 (53 to 629)	1865 (444 to 5036)	225 (53 to 606)	-0.03 (-0.1 to 0.04)	0.342
Slovenia	429 (101 to 1180)	219 (52 to 599)	1196 (294 to 3163)	227 (56 to 603)	0.13 (-0.02 to 0.27)	0.083
Solomon Islands	10 (2 to 30)	254 (56 to 746)	33 (7 to 96)	256 (58 to 735)	0.02 (-0.04 to 0.07)	0.544
Somalia	177 (40 to 507)	247 (55 to 702)	422 (94 to 1228)	267 (60 to 780)	0.27 (0.21 to 0.33)	<0.001
South Africa	2953 (683 to 8171)	230 (53 to 633)	6246 (1474 to 17572)	245 (58 to 683)	0.22 (0.15 to 0.3)	<0.001
South Sudan	293 (65 to 829)	251 (55 to 703)	400 (87 to 1118)	256 (56 to 710)	0.05 (0 to 0.1)	0.055
Spain	12976 (3257 to 34765)	292 (73 to 777)	35657 (9310 to 89232)	273 (71 to 687)	-0.21 (-0.24 to -0.18)	<0.001
Sri Lanka	1044 (245 to 2942)	203 (47 to 570)	3592 (811 to 9786)	204 (46 to 553)	0.01 (-0.08 to 0.11)	0.777
Sudan	1181 (274 to 3419)	274 (63 to 780)	2589 (586 to 7108)	254 (58 to 692)	-0.26 (-0.27 to -0.24)	<0.001
Suriname	34 (8 to 95)	179 (42 to 499)	85 (19 to 230)	176 (40 to 478)	-0.03 (-0.17 to 0.11)	0.696
Sweden	4134 (1009 to 11014)	271 (66 to 719)	6839 (1729 to 17296)	255 (65 to 648)	-0.19 (-0.26 to -0.12)	<0.001

Switzerland	2824 (690 to 7584)	273 (66 to 731)	6269 (1637 to 15479)	271 (71 to 672)	-0.01 (-0.13 to 0.11)	0.907
Syrian Arab Republic	864 (204 to 2439)	276 (65 to 779)	1563 (358 to 4505)	260 (59 to 746)	-0.21 (-0.3 to -0.12)	<0.001
Taiwan (Province of China)	1512 (362 to 4199)	220 (53 to 600)	9757 (2448 to 24997)	230 (58 to 595)	0.2 (-0.01 to 0.4)	0.057
Tajikistan	418 (97 to 1135)	227 (53 to 616)	610 (144 to 1696)	235 (56 to 647)	0.13 (0.05 to 0.2)	0.001
Thailand	4457 (1046 to 12368)	239 (56 to 656)	22299 (5423 to 57179)	227 (55 to 582)	-0.17 (-0.25 to -0.09)	<0.001
Timor-Leste	22 (5 to 64)	202 (45 to 580)	98 (22 to 285)	226 (52 to 652)	0.36 (0.32 to 0.41)	<0.001
Togo	101 (23 to 292)	201 (45 to 573)	290 (64 to 840)	218 (48 to 622)	0.23 (0.18 to 0.28)	<0.001
Tokelau	0 (0 to 1)	306 (71 to 873)	0 (0 to 1)	275 (64 to 750)	-0.34 (-0.37 to -0.32)	<0.001
Tonga	8 (2 to 23)	287 (68 to 788)	17 (4 to 47)	281 (67 to 753)	-0.06 (-0.14 to 0.01)	0.098
Trinidad and Tobago	86 (20 to 249)	181 (41 to 520)	265 (61 to 732)	170 (39 to 469)	-0.2 (-0.26 to -0.13)	<0.001
Tunisia	686 (162 to 1913)	308 (73 to 835)	2574 (602 to 7031)	283 (67 to 770)	-0.28 (-0.33 to -0.23)	<0.001
Turkey	6466 (1540 to 17670)	328 (78 to 890)	19173 (4604 to 53041)	286 (69 to 786)	-0.41 (-0.61 to -0.21)	<0.001
Turkmenistan	236 (56 to 660)	229 (54 to 634)	532 (124 to 1471)	214 (50 to 594)	-0.19 (-0.3 to -0.09)	<0.001
Tuvalu	1 (0 to 2)	292 (68 to 855)	1 (0 to 4)	280 (64 to 804)	-0.14 (-0.17 to -0.1)	<0.001
Uganda	660 (155 to 1815)	241 (56 to 655)	1920 (445 to 5401)	293 (69 to 809)	0.65 (0.6 to 0.7)	<0.001
Ukraine	10970 (2553 to 31300)	235 (54 to 665)	15809 (3643 to 44222)	228 (53 to 638)	-0.09 (-0.16 to -0.02)	0.009
United Arab Emirates	40 (9 to 110)	314 (75 to 847)	104 (24 to 292)	264 (62 to 723)	-0.59 (-0.88 to -0.29)	<0.001
United Kingdom	21251 (5121 to 57846)	260 (63 to 705)	36980 (9274 to 96204)	249 (62 to 649)	-0.17 (-0.22 to -0.12)	<0.001

United Republic of Tanzania	1209 (284 to 3418)	280 (66 to 767)	3584 (854 to 10053)	281 (67 to 776)	0.01 (-0.01 to 0.04)	0.317
United States Virgin Islands	8 (2 to 22)	324 (81 to 848)	24 (6 to 66)	319 (81 to 815)	-0.44 (-0.48 to -0.41)	<0.001
United States of America	97510 (24358 to 256019)	189 (44 to 541)	193180 (49263 to 492220)	164 (38 to 449)	-0.06 (-0.09 to -0.02)	0.001
Uruguay	720 (170 to 1993)	226 (54 to 624)	1544 (379 to 4079)	231 (57 to 614)	0.05 (0.02 to 0.08)	0.001
Uzbekistan	1836 (436 to 5040)	221 (52 to 605)	2761 (649 to 7773)	210 (49 to 585)	-0.18 (-0.25 to -0.1)	<0.001
Vanuatu	5 (1 to 14)	248 (54 to 714)	16 (4 to 46)	250 (57 to 726)	0.03 (-0.01 to 0.06)	0.17
Venezuela (Bolivarian Republic of)	1385 (335 to 3812)	230 (55 to 627)	5425 (1301 to 14251)	224 (54 to 593)	-0.07 (-0.18 to 0.03)	0.184
Viet Nam	6923 (1584 to 18969)	266 (61 to 724)	17978 (4257 to 50050)	295 (70 to 816)	0.35 (0.32 to 0.37)	<0.001
Yemen	555 (128 to 1583)	298 (69 to 836)	1794 (396 to 5298)	284 (63 to 834)	-0.16 (-0.18 to -0.13)	<0.001
Zambia	269 (62 to 764)	239 (54 to 672)	702 (157 to 1986)	267 (61 to 739)	0.37 (0.27 to 0.46)	<0.001
Zimbabwe	457 (108 to 1257)	270 (64 to 736)	602 (136 to 1735)	251 (57 to 707)	-0.22 (-0.28 to -0.16)	<0.001

Note: Estimates are for individuals aged over 65 years. AAPCs=average annual percent changes. CI=confidence interval. P value for the significant test of AAPCs. Numbers in parentheses are 95% uncertainty intervals (Cases and age-standardized rate) and 95% confidence interval (AAPCs).

Table S9. Age standardized DALYs of ADOD in people aged ≥65 years in 2021 and their AAPCs between 1990-2021 in 204 countries and territories.

Country or territory	No in 1990 (000s)	Age standardised rate in 1990 (per 100 000)	No in 2021 (000s)	Age standardised rate in 2021 (per 100 000)	AAPC (95% CI)	P value
Afghanistan	22678 (9533 to 54503)	6159 (2540 to 14649)	28745 (12411 to 66489)	5883 (2501 to 13607)	-0.15 (-0.17 to -0.13)	<0.001
Albania	5460 (2520 to 12128)	4095 (1879 to 9074)	14443 (6745 to 32070)	3975 (1833 to 8850)	-0.11 (-0.18 to -0.03)	0.005
Algeria	32267 (15374 to 73007)	5222 (2353 to 12079)	106252 (49827 to 232906)	4886 (2222 to 10869)	-0.21 (-0.22 to -0.2)	<0.001
American Samoa	45 (20 to 103)	4387 (1907 to 10085)	116 (53 to 256)	4099 (1845 to 9116)	-0.22 (-0.25 to -0.18)	<0.001
Andorra	183 (87 to 385)	4677 (2163 to 9908)	692 (315 to 1499)	4364 (2010 to 9463)	-0.23 (-0.3 to -0.17)	<0.001
Angola	7774 (3523 to 17836)	5231 (2305 to 12099)	27914 (11887 to 66759)	5839 (2441 to 13899)	0.36 (0.33 to 0.39)	<0.001
Antigua and Barbuda	177 (84 to 392)	3293 (1562 to 7328)	234 (113 to 520)	3138 (1485 to 7017)	-0.15 (-0.2 to -0.1)	<0.001
Argentina	94671 (44637 to 209002)	3926 (1819 to 8696)	200611 (92792 to 434679)	3783 (1749 to 8196)	-0.12 (-0.14 to -0.1)	<0.001
Armenia	6852 (3251 to 15060)	3908 (1841 to 8606)	15505 (7118 to 33833)	4064 (1864 to 8895)	0.11 (0.03 to 0.18)	0.005

Australia	73044 (34463 to 158455)	4519 (2107 to 9819)	197235 (89028 to 420855)	4081 (1857 to 8701)	-0.32 (-0.36 to -0.29)	<0.001
Austria	52323 (24255 to 116482)	4679 (2143 to 10435)	93343 (43040 to 200057)	4440 (2068 to 9510)	-0.16 (-0.18 to -0.15)	<0.001
Azerbaijan	12928 (6111 to 28338)	4059 (1883 to 8981)	22325 (10375 to 50517)	3919 (1812 to 8831)	-0.12 (-0.16 to -0.07)	<0.001
Bahamas	348 (169 to 759)	3245 (1553 to 7088)	875 (419 to 1893)	3153 (1501 to 6836)	-0.1 (-0.15 to -0.05)	<0.001
Bahrain	309 (145 to 695)	5357 (2386 to 12279)	1387 (654 to 3162)	4792 (2166 to 11100)	-0.36 (-0.42 to -0.31)	<0.001
Bangladesh	86142 (39180 to 194975)	2918 (1309 to 6637)	291759 (127591 to 692566)	3116 (1340 to 7454)	0.24 (0.13 to 0.34)	<0.001
Barbados	922 (452 to 2016)	3213 (1542 to 7093)	1471 (698 to 3193)	3161 (1493 to 6871)	-0.05 (-0.15 to 0.04)	0.271
Belarus	41894 (19843 to 92613)	4076 (1912 to 9042)	59242 (27712 to 130406)	4017 (1880 to 8837)	-0.04 (-0.09 to 0)	0.05
Belgium	75563 (35102 to 162549)	5316 (2444 to 11454)	132564 (60545 to 278398)	4770 (2205 to 10034)	-0.34 (-0.42 to -0.26)	<0.001
Belize	267 (128 to 569)	3325 (1600 to 7089)	671 (321 to 1445)	3225 (1544 to 6931)	-0.11 (-0.19 to -0.04)	0.003
Benin	4252 (1881 to 9803)	3234 (1403 to 7515)	9126 (3922 to 21502)	3173 (1332 to 7494)	-0.06 (-0.12 to 0.01)	0.102
Bermuda	157 (76 to 342)	3380 (1598 to 7406)	468 (224 to 983)	3267 (1571 to 6859)	-0.08 (-0.28 to 0.12)	0.413

Bhutan	337 (152 to 747)	2994 (1333 to 6664)	1530 (629 to 3662)	3406 (1389 to 8153)	0.42 (0.38 to 0.45)	<0.001
Bolivia (Plurinational State of)	5439 (2496 to 12403)	2930 (1317 to 6734)	17119 (7880 to 38022)	2934 (1328 to 6520)	0.01 (-0.01 to 0.03)	0.491
Bosnia and Herzegovina	9299 (4389 to 20713)	3918 (1827 to 8686)	21577 (10316 to 46266)	3802 (1810 to 8138)	-0.1 (-0.16 to -0.03)	0.003
Botswana	1090 (488 to 2548)	4260 (1848 to 10145)	3090 (1383 to 7110)	4131 (1824 to 9442)	-0.1 (-0.21 to 0)	0.05
Brazil	277430 (125390 to 624613)	5142 (2289 to 11510)	1052122 (479451 to 2285545)	5047 (2299 to 10961)	-0.06 (-0.08 to -0.04)	<0.001
Brunei Darussalam	219 (101 to 497)	3950 (1750 to 9061)	618 (284 to 1341)	4064 (1794 to 8967)	0.07 (0.02 to 0.12)	0.005
Bulgaria	29530 (14319 to 65201)	3907 (1814 to 8814)	52319 (25185 to 114625)	3880 (1832 to 8610)	-0.03 (-0.06 to 0.01)	0.15
Burkina Faso	8320 (3565 to 19343)	3794 (1558 to 8956)	17421 (7399 to 41310)	3466 (1429 to 8228)	-0.29 (-0.39 to -0.18)	<0.001
Burundi	5302 (2453 to 12007)	4209 (1889 to 9568)	9849 (4186 to 23677)	4402 (1831 to 10558)	0.15 (0.1 to 0.19)	<0.001
Cabo Verde	647 (294 to 1452)	3054 (1374 to 6853)	1098 (478 to 2495)	3114 (1367 to 7060)	0.05 (0.03 to 0.08)	<0.001
Cambodia	9137 (4277 to 20306)	4102 (1873 to 9194)	30490 (13580 to 70491)	4629 (2006 to 10760)	0.39 (0.37 to 0.42)	<0.001
Cameroon	7841 (3308 to 18150)	3495 (1437 to 8164)	19940 (8460 to 48855)	3285 (1354 to 8110)	-0.22 (-0.29 to -0.16)	<0.001

Canada	128906 (65844 to 263844)	4567 (2317 to 9380)	317546 (155619 to 649124)	4191 (2070 to 8553)	-0.28 (-0.32 to -0.24)	<0.001
Central African Republic	2429 (1063 to 5504)	5923 (2541 to 13389)	4283 (1860 to 9627)	5843 (2452 to 13170)	-0.05 (-0.1 to 0.01)	0.088
Chad	5566 (2510 to 12754)	3137 (1377 to 7255)	9080 (3884 to 21089)	3116 (1304 to 7273)	-0.03 (-0.05 to 0)	0.028
Chile	26596 (12603 to 58564)	3737 (1744 to 8261)	87254 (41030 to 185851)	3694 (1738 to 7875)	-0.03 (-0.07 to 0)	0.049
China	2209476 (973328 to 4991690)	5451 (2327 to 12426)	8948652 (4182308 to 19508573)	5762 (2654 to 12574)	0.09 (-0.02 to 0.21)	0.111
Colombia	41657 (20349 to 90619)	3622 (1757 to 7881)	186889 (91705 to 390833)	3608 (1779 to 7573)	-0.02 (-0.04 to 0)	0.105
Comoros	410 (184 to 921)	4449 (1933 to 10010)	1381 (576 to 3312)	4811 (1973 to 11491)	0.24 (0.21 to 0.28)	<0.001
Congo	2645 (1159 to 6090)	6024 (2511 to 13920)	6567 (2755 to 15186)	5861 (2394 to 13434)	-0.09 (-0.11 to -0.06)	<0.001
Cook Islands	33 (15 to 75)	4513 (1963 to 10372)	87 (40 to 191)	4026 (1828 to 8821)	-0.37 (-0.42 to -0.33)	<0.001
Costa Rica	5025 (2418 to 10733)	3676 (1761 to 7854)	18112 (8737 to 37632)	3592 (1746 to 7472)	-0.05 (-0.15 to 0.04)	0.281
Coted'Ivoire	5099 (2221 to 11808)	3321 (1391 to 7708)	16592 (7096 to 39354)	3270 (1361 to 7804)	-0.05 (-0.09 to -0.02)	<0.001
Croatia	17037 (8040 to 37629)	4040 (1882 to 8961)	35764 (16751 to 78465)	3890 (1810 to 8557)	-0.11 (-0.21 to -0.02)	0.024
Cuba	25352 (12216 to 56228)	3080 (1452 to 6888)	59007 (27868 to 130662)	3044 (1449 to 6718)	-0.05 (-0.09 to -0.02)	0.002

Cyprus	2494 (1139 to 5790)	5135 (2206 to 12162)	7850 (3629 to 17389)	4603 (2049 to 10392)	-0.36 (-0.43 to -0.28)	<0.001
Czechia	44270 (20831 to 99682)	3916 (1810 to 8847)	84842 (39588 to 183154)	3888 (1811 to 8400)	-0.01 (-0.06 to 0.03)	0.531
Democratic People's Republic of Korea	35378 (15915 to 79792)	4314 (1907 to 9776)	101501 (45083 to 229826)	4392 (1923 to 9947)	0.06 (0.04 to 0.08)	<0.001
Democratic Republic of the Congo	33386 (14554 to 78024)	5416 (2296 to 12599)	97635 (41017 to 231130)	6152 (2545 to 14397)	0.41 (0.38 to 0.44)	<0.001
Denmark	33828 (15102 to 76861)	4175 (1849 to 9499)	51237 (21519 to 115683)	4006 (1688 to 9041)	-0.11 (-0.23 to 0.01)	0.063
Djibouti	228 (99 to 541)	4668 (1982 to 10982)	1202 (510 to 2768)	4919 (2027 to 11394)	0.17 (0.13 to 0.2)	<0.001
Dominica	165 (79 to 361)	3319 (1565 to 7332)	208 (99 to 455)	3241 (1534 to 7132)	-0.08 (-0.11 to -0.04)	<0.001
Dominican Republic	7933 (3818 to 17044)	3351 (1573 to 7293)	27197 (13020 to 57832)	3280 (1568 to 6979)	-0.07 (-0.12 to -0.02)	0.01
Ecuador	10398 (4880 to 23205)	2805 (1296 to 6314)	35290 (16523 to 77199)	2704 (1252 to 5956)	-0.12 (-0.16 to -0.09)	<0.001
Egypt	59082 (27268 to 135557)	5039 (2250 to 11672)	125982 (58912 to 282162)	4634 (2114 to 10374)	-0.27 (-0.37 to -0.16)	<0.001
El Salvador	8935 (4254 to 19376)	3684 (1759 to 7991)	23300 (11133 to 48273)	3646 (1762 to 7558)	-0.02 (-0.11 to 0.07)	0.652
Equatorial Guinea	462 (204 to 1077)	5422 (2331 to 12646)	1386 (588 to 3235)	5755 (2388 to 13407)	0.18 (0.13 to 0.23)	<0.001

Eritrea	1421 (641 to 3269)	4453 (1957 to 10272)	5387 (2254 to 12806)	5107 (2058 to 12072)	0.45 (0.43 to 0.46)	<0.001
Estonia	6633 (3150 to 14795)	4020 (1876 to 9025)	12844 (5894 to 28023)	4137 (1904 to 9034)	0.09 (0.07 to 0.12)	<0.001
Eswatini	597 (266 to 1376)	4265 (1844 to 9889)	1057 (455 to 2455)	4265 (1808 to 9812)	0.01 (-0.03 to 0.05)	0.504
Ethiopia	37625 (16209 to 89582)	4781 (2012 to 11291)	123614 (51779 to 288520)	4884 (2015 to 11341)	0.07 (0.04 to 0.09)	<0.001
Fiji	661 (298 to 1519)	4293 (1887 to 9930)	1470 (689 to 3182)	4118 (1866 to 9107)	-0.15 (-0.17 to -0.12)	<0.001
Finland	31618 (14271 to 69950)	4974 (2220 to 11001)	68455 (30256 to 146298)	4571 (2040 to 9766)	-0.29 (-0.32 to -0.26)	<0.001
France	330081 (149627 to 733494)	4029 (1808 to 8979)	670276 (298160 to 1440186)	3812 (1727 to 8163)	-0.18 (-0.2 to -0.16)	<0.001
Gabon	2082 (894 to 4697)	6090 (2551 to 13631)	3164 (1355 to 7273)	6046 (2530 to 13864)	-0.02 (-0.06 to 0.01)	0.221
Gambia	587 (261 to 1337)	3462 (1485 to 7999)	1927 (791 to 4717)	3439 (1381 to 8417)	-0.02 (-0.15 to 0.1)	0.713
Georgia	18363 (8737 to 40677)	4001 (1881 to 8898)	25491 (11814 to 55665)	4061 (1891 to 8915)	0.03 (-0.1 to 0.16)	0.637
Germany	632425 (302973 to 1342269)	5343 (2547 to 11331)	1233817 (586875 to 2517246)	5328 (2547 to 10874)	0 (-0.05 to 0.05)	0.968
Ghana	8895 (3975 to 20456)	3001 (1306 to 6954)	27459 (11524 to 66774)	3318 (1355 to 8112)	0.32 (0.29 to 0.35)	<0.001

Greece	57809 (27042 to 128565)	4606 (2116 to 10299)	138084 (63227 to 297733)	4475 (2071 to 9628)	-0.1 (-0.14 to -0.07)	<0.001
Greenland	69 (33 to 154)	4968 (2297 to 11235)	175 (84 to 371)	4811 (2265 to 10255)	-0.09 (-0.22 to 0.03)	0.151
Grenada	269 (125 to 591)	3419 (1603 to 7512)	240 (115 to 528)	3303 (1561 to 7296)	-0.04 (-0.15 to 0.06)	0.401
Guam	140 (64 to 312)	4253 (1892 to 9557)	766 (380 to 1489)	3681 (1830 to 7281)	-0.48 (-0.59 to -0.38)	<0.001
Guatemala	6544 (3150 to 14565)	3722 (1737 to 8403)	28986 (14092 to 61426)	3628 (1735 to 7779)	-0.09 (-0.11 to -0.07)	<0.001
Guinea	6534 (2893 to 14820)	3114 (1350 to 7131)	10870 (4669 to 25340)	3230 (1364 to 7544)	0.12 (0.09 to 0.15)	<0.001
Guinea-Bissau	584 (254 to 1352)	3307 (1388 to 7784)	969 (409 to 2343)	3402 (1391 to 8316)	0.08 (0.05 to 0.11)	<0.001
Guyana	723 (351 to 1556)	3075 (1471 to 6679)	1202 (581 to 2620)	3095 (1469 to 6764)	0.03 (-0.13 to 0.19)	0.69
Haiti	5312 (2548 to 11751)	3424 (1608 to 7690)	11478 (5418 to 25670)	3229 (1486 to 7282)	-0.19 (-0.2 to -0.17)	<0.001
Honduras	5019 (2381 to 11003)	3972 (1860 to 8734)	16808 (7593 to 38015)	4286 (1897 to 9726)	0.26 (0.19 to 0.32)	<0.001
Hungary	44792 (21069 to 99991)	3932 (1806 to 8840)	78702 (36875 to 169852)	3874 (1816 to 8361)	-0.04 (-0.08 to 0.01)	0.129
Iceland	1331 (639 to 2835)	4731 (2274 to 10078)	2824 (1328 to 5832)	4492 (2135 to 9290)	-0.17 (-0.22 to -0.11)	<0.001

India	586919 (270001 to 1365248)	2608 (1186 to 6069)	2342639 (1019520 to 5458039)	3062 (1316 to 7118)	0.53 (0.42 to 0.65)	<0.001
Indonesia	179143 (86509 to 393081)	3609 (1716 to 7978)	491344 (225533 to 1137126)	4231 (1879 to 9880)	0.52 (0.49 to 0.55)	<0.001
Iran (Islamic Republic of)	62849 (29120 to 140178)	5171 (2369 to 11532)	260061 (120576 to 565995)	4827 (2228 to 10511)	-0.22 (-0.24 to -0.2)	<0.001
Iraq	30058 (13905 to 64831)	5017 (2320 to 10814)	60667 (28104 to 133282)	4855 (2206 to 10742)	-0.1 (-0.13 to -0.07)	<0.001
Ireland	15614 (7284 to 34863)	4641 (2113 to 10429)	33896 (15730 to 71954)	4356 (2022 to 9249)	-0.2 (-0.26 to -0.15)	<0.001
Israel	17552 (8192 to 39133)	4505 (2055 to 10096)	56289 (25602 to 119446)	4393 (2011 to 9320)	-0.09 (-0.14 to -0.03)	0.002
Italy	416478 (184282 to 929915)	5350 (2327 to 11958)	968469 (446781 to 2019282)	5294 (2466 to 11066)	-0.03 (-0.09 to 0.03)	0.31
Jamaica	5802 (2879 to 12233)	3435 (1699 to 7255)	10568 (5128 to 22331)	3308 (1640 to 6986)	-0.12 (-0.16 to -0.07)	<0.001
Japan	616356 (278167 to 1354501)	4737 (2107 to 10383)	2461667 (1135634 to 4986405)	4686 (2202 to 9544)	-0.03 (-0.07 to 0)	0.074
Jordan	3334 (1567 to 7246)	4993 (2320 to 10878)	17715 (8570 to 39001)	4604 (2187 to 10137)	-0.26 (-0.35 to -0.18)	<0.001
Kazakhstan	33594 (15792 to 74270)	4002 (1862 to 8858)	40570 (19081 to 90753)	3782 (1746 to 8515)	-0.19 (-0.24 to -0.15)	<0.001
Kenya	19962 (8790 to 45085)	4304 (1858 to 9771)	54565 (22658 to 123744)	4901 (1991 to 11024)	0.42 (0.37 to 0.47)	<0.001

Kiribati	75 (34 to 170)	4506 (2015 to 10302)	144 (65 to 327)	4942 (2168 to 11273)	0.3 (0.28 to 0.32)	<0.001
Kuwait	1460 (692 to 3149)	5175 (2442 to 11118)	7664 (3620 to 16271)	4699 (2234 to 9971)	-0.31 (-0.51 to -0.11)	0.003
Kyrgyzstan	8440 (3967 to 18498)	4052 (1875 to 8921)	11223 (5302 to 24328)	3932 (1854 to 8514)	-0.1 (-0.14 to -0.06)	<0.001
Lao People's Democratic Republic	3738 (1807 to 8211)	3891 (1831 to 8653)	10011 (4608 to 22856)	4075 (1842 to 9374)	0.15 (0.12 to 0.19)	<0.001
Latvia	11983 (5655 to 26664)	4021 (1872 to 8980)	17910 (8392 to 39410)	4016 (1882 to 8834)	0 (-0.04 to 0.04)	0.942
Lebanon	6470 (3118 to 14059)	4859 (2313 to 10623)	28494 (13749 to 60673)	4718 (2283 to 10026)	-0.09 (-0.15 to -0.04)	0.001
Lesotho	2235 (1013 to 4864)	4206 (1869 to 9238)	2350 (1031 to 5606)	4378 (1903 to 10295)	0.15 (0.09 to 0.2)	<0.001
Liberia	2040 (900 to 4912)	3109 (1322 to 7508)	3268 (1400 to 7860)	3093 (1298 to 7461)	-0.02 (-0.12 to 0.07)	0.613
Libya	7427 (3349 to 15912)	5634 (2549 to 12057)	16291 (7374 to 36039)	5149 (2321 to 11400)	-0.3 (-0.37 to -0.22)	<0.001
Lithuania	15338 (7156 to 33960)	3999 (1849 to 8900)	25937 (12012 to 57046)	4025 (1863 to 8868)	0.02 (-0.03 to 0.08)	0.381
Luxembourg	1564 (752 to 3411)	3372 (1587 to 7428)	3549 (1618 to 7704)	3195 (1471 to 6927)	-0.17 (-0.24 to -0.1)	<0.001
Madagascar	9885 (4642 to 22216)	3658 (1687 to 8289)	16313 (7597 to 36928)	3714 (1687 to 8392)	0.05 (0.01 to 0.09)	0.009

Malawi	7770 (3488 to 18035)	4303 (1872 to 10013)	17177 (7428 to 40043)	4733 (1995 to 11103)	0.32 (0.28 to 0.36)	<0.001
Malaysia	28468 (13065 to 62821)	4435 (2033 to 9788)	82695 (36605 to 189260)	4451 (1940 to 10204)	0 (-0.07 to 0.07)	0.962
Maldives	132 (64 to 290)	3554 (1682 to 7958)	811 (383 to 1769)	3817 (1799 to 8345)	0.22 (0.2 to 0.25)	<0.001
Mali	6742 (2835 to 15633)	3710 (1498 to 8752)	15601 (6373 to 37938)	3669 (1456 to 8878)	-0.03 (-0.06 to 0)	0.04
Malta	1506 (704 to 3338)	4600 (2118 to 10200)	4679 (2168 to 9850)	4395 (2040 to 9267)	-0.15 (-0.23 to -0.06)	0.001
Marshall Islands	35 (16 to 81)	4561 (1948 to 10676)	52 (23 to 120)	4256 (1810 to 10016)	-0.22 (-0.24 to -0.21)	<0.001
Mauritania	1962 (867 to 4491)	3347 (1438 to 7712)	4415 (1887 to 10673)	3303 (1389 to 8022)	-0.06 (-0.13 to 0.01)	0.113
Mauritius	1714 (807 to 3812)	4120 (1892 to 9205)	5607 (2624 to 12375)	3904 (1822 to 8627)	-0.2 (-0.28 to -0.11)	<0.001
Mexico	84435 (41414 to 183598)	3095 (1492 to 6795)	284869 (136468 to 627003)	2968 (1415 to 6542)	-0.14 (-0.17 to -0.12)	<0.001
Micronesia (Federated States of)	132 (59 to 305)	4821 (2118 to 11220)	151 (68 to 349)	4760 (2108 to 11047)	-0.04 (-0.06 to -0.02)	<0.001
Monaco	370 (171 to 817)	4732 (2175 to 10436)	556 (250 to 1179)	4736 (2159 to 10064)	0 (-0.02 to 0.03)	0.782
Mongolia	2742 (1280 to 6054)	4237 (1932 to 9405)	4569 (2161 to 10148)	4124 (1922 to 9149)	-0.09 (-0.21 to 0.04)	0.166

Montenegro	1898 (893 to 4177)	3987 (1865 to 8778)	2896 (1364 to 6359)	3908 (1801 to 8666)	-0.05 (-0.12 to 0.01)	0.12
Morocco	48241 (22829 to 106396)	5086 (2374 to 11243)	109458 (49813 to 243913)	4895 (2197 to 10942)	-0.12 (-0.14 to -0.11)	<0.001
Mozambique	13118 (5740 to 30023)	4689 (1991 to 10824)	24907 (10344 to 60561)	5036 (2043 to 12269)	0.25 (0.2 to 0.31)	<0.001
Myanmar	45612 (22268 to 98894)	3887 (1853 to 8511)	123043 (56132 to 279230)	4069 (1830 to 9277)	0.16 (0.14 to 0.17)	<0.001
Namibia	1260 (574 to 2867)	4089 (1829 to 9319)	3181 (1398 to 7523)	4386 (1893 to 10423)	0.24 (0.21 to 0.26)	<0.001
Nauru	8 (3 to 18)	4503 (1934 to 10704)	11 (5 to 26)	4541 (1965 to 10591)	0.03 (0.02 to 0.03)	<0.001
Nepal	13127 (6273 to 28895)	2827 (1335 to 6286)	44927 (19773 to 103943)	3138 (1350 to 7297)	0.34 (0.3 to 0.37)	<0.001
Netherlands	93116 (42917 to 199340)	5049 (2311 to 10832)	183020 (83028 to 391291)	4981 (2264 to 10653)	-0.04 (-0.08 to -0.01)	0.009
New Zealand	15768 (7290 to 34568)	4772 (2177 to 10452)	38368 (17562 to 82399)	4519 (2074 to 9702)	-0.18 (-0.22 to -0.14)	<0.001
Nicaragua	3772 (1845 to 7943)	3684 (1794 to 7756)	12660 (6251 to 26031)	3595 (1758 to 7435)	-0.09 (-0.13 to -0.06)	<0.001
Niger	3938 (1731 to 9028)	3343 (1421 to 7769)	12767 (5488 to 30162)	3339 (1383 to 7930)	0 (-0.05 to 0.04)	0.865
Nigeria	74941 (32788 to 174117)	3002 (1272 to 7043)	148576 (61579 to 360985)	3114 (1265 to 7551)	0.1 (0.05 to 0.15)	<0.001

Niue	11 (5 to 25)	4848 (2050 to 11262)	7 (3 to 17)	4408 (1931 to 10079)	-0.31 (-0.34 to -0.28)	<0.001
North Macedonia	4864 (2312 to 10675)	3758 (1784 to 8227)	7874 (3793 to 17363)	3797 (1764 to 8550)	0.03 (0 to 0.07)	0.077
Northern Mariana Islands	23 (11 to 51)	4266 (1893 to 9651)	101 (46 to 227)	4162 (1862 to 9445)	-0.09 (-0.17 to -0.01)	0.035
Norway	35306 (16518 to 76625)	4965 (2316 to 10774)	49068 (22120 to 106559)	4444 (2025 to 9645)	-0.35 (-0.38 to -0.32)	<0.001
Oman	1849 (851 to 4070)	5254 (2384 to 11526)	4094 (1914 to 9387)	4842 (2225 to 11033)	-0.28 (-0.34 to -0.21)	<0.001
Pakistan	109745 (48523 to 253610)	3133 (1359 to 7297)	216577 (89620 to 506945)	3382 (1374 to 7925)	0.24 (0.2 to 0.28)	<0.001
Palau	23 (10 to 52)	4400 (1877 to 10176)	41 (18 to 95)	3935 (1712 to 9335)	-0.36 (-0.4 to -0.33)	<0.001
Palestine	2794 (1300 to 6362)	5065 (2318 to 11608)	6196 (2950 to 13949)	4742 (2208 to 10673)	-0.22 (-0.24 to -0.2)	<0.001
Panama	4012 (1939 to 8554)	3522 (1697 to 7506)	14544 (6979 to 29923)	3508 (1694 to 7215)	0.02 (-0.01 to 0.04)	0.218
Papua New Guinea	2780 (1309 to 6127)	4146 (1904 to 9244)	8240 (3791 to 18851)	3943 (1777 to 9114)	-0.16 (-0.19 to -0.13)	<0.001
Paraguay	7871 (3621 to 17113)	4708 (2161 to 10226)	21434 (9553 to 46486)	4696 (2100 to 10177)	0 (-0.07 to 0.07)	0.965
Peru	24341 (11098 to 54793)	2737 (1246 to 6162)	79083 (36596 to 170083)	2669 (1243 to 5732)	-0.06 (-0.18 to 0.06)	0.312
Philippines	60905 (29528 to 134833)	3925 (1836 to 8848)	199776 (92071 to 437514)	4100 (1867 to 9001)	0.14 (0.07 to 0.2)	<0.001

Poland	137707 (64889 to 307247)	4129 (1920 to 9209)	288311 (133384 to 634153)	4004 (1857 to 8810)	-0.11 (-0.13 to -0.09)	<0.001
Portugal	49237 (22615 to 110209)	4630 (2068 to 10418)	133026 (60709 to 284366)	4506 (2075 to 9625)	-0.09 (-0.12 to -0.05)	<0.001
Puerto Rico	9938 (4764 to 21662)	3355 (1579 to 7379)	28425 (13663 to 59071)	3202 (1553 to 6671)	-0.17 (-0.23 to -0.12)	<0.001
Qatar	161 (73 to 368)	5341 (2347 to 12301)	1079 (494 to 2445)	4829 (2150 to 10997)	-0.33 (-0.49 to -0.16)	<0.001
Republic of Korea	85699 (37671 to 193462)	6032 (2573 to 13637)	417189 (192297 to 859669)	5141 (2355 to 10599)	-0.52 (-0.55 to -0.49)	<0.001
Republic of Moldova	10496 (4943 to 23389)	3962 (1810 to 8935)	20802 (9911 to 44339)	3781 (1808 to 8070)	-0.15 (-0.17 to -0.12)	<0.001
Romania	71185 (34103 to 158572)	3895 (1817 to 8771)	149196 (69699 to 327351)	3889 (1815 to 8536)	-0.01 (-0.06 to 0.03)	0.536
Russian Federation	509500 (242313 to 1137144)	4148 (1930 to 9311)	878207 (409956 to 1959031)	4047 (1891 to 9017)	-0.08 (-0.12 to -0.04)	<0.001
Rwanda	5594 (2507 to 13086)	4483 (1943 to 10527)	15361 (6454 to 37071)	5036 (2067 to 12105)	0.38 (0.34 to 0.42)	<0.001
Saint Kitts and Nevis	96 (47 to 212)	3171 (1489 to 7109)	123 (59 to 270)	3095 (1471 to 6790)	-0.08 (-0.27 to 0.11)	0.404
Saint Lucia	208 (100 to 457)	3282 (1534 to 7346)	639 (305 to 1394)	3210 (1525 to 7027)	-0.11 (-0.16 to -0.05)	<0.001
Saint Vincent and the Grenadines	189 (91 to 414)	3466 (1618 to 7685)	346 (168 to 750)	3153 (1509 to 6914)	-0.32 (-0.42 to -0.21)	<0.001

Samoa	236 (103 to 547)	4857 (2090 to 11204)	416 (179 to 968)	4629 (1964 to 10783)	-0.16 (-0.18 to -0.14)	<0.001
San Marino	152 (72 to 324)	4450 (2088 to 9455)	394 (184 to 808)	4007 (1911 to 8219)	-0.36 (-0.46 to -0.25)	<0.001
Sao Tome and Principe	138 (61 to 324)	3147 (1365 to 7400)	189 (81 to 442)	3072 (1297 to 7217)	-0.09 (-0.13 to -0.04)	<0.001
Saudi Arabia	16223 (7294 to 36376)	5149 (2278 to 11561)	29898 (13451 to 68191)	4685 (2061 to 10730)	-0.31 (-0.33 to -0.29)	<0.001
Senegal	6060 (2621 to 14212)	3329 (1401 to 7854)	15839 (6547 to 38102)	3514 (1424 to 8497)	0.16 (0.03 to 0.29)	0.015
Serbia	26361 (12347 to 59370)	3894 (1794 to 8880)	61014 (28393 to 135026)	3843 (1786 to 8509)	-0.05 (-0.09 to -0.01)	0.01
Seychelles	204 (94 to 455)	4168 (1924 to 9300)	309 (141 to 680)	3919 (1772 to 8638)	-0.2 (-0.23 to -0.17)	<0.001
Sierra Leone	3962 (1793 to 9120)	3085 (1359 to 7194)	6398 (2811 to 15169)	3056 (1312 to 7287)	-0.03 (-0.07 to 0.01)	0.125
Singapore	4457 (2083 to 9896)	3413 (1560 to 7593)	24169 (11604 to 50146)	3363 (1617 to 6975)	-0.05 (-0.13 to 0.02)	0.177
Slovakia	18967 (8892 to 42316)	3983 (1843 to 8888)	33645 (15641 to 72987)	3931 (1821 to 8531)	-0.04 (-0.09 to 0.01)	0.15
Slovenia	7998 (3734 to 17737)	3880 (1798 to 8593)	19664 (9110 to 41975)	3936 (1839 to 8390)	0.06 (-0.05 to 0.17)	0.254
Solomon Islands	228 (103 to 527)	4358 (1921 to 10246)	683 (308 to 1579)	4349 (1912 to 10162)	-0.02 (-0.05 to 0.02)	0.38

Somalia	3701 (1650 to 8536)	4223 (1838 to 9815)	9717 (4259 to 22733)	4523 (1941 to 10791)	0.23 (0.2 to 0.26)	<0.001
South Africa	55662 (25879 to 123980)	4015 (1846 to 8961)	118449 (53353 to 271950)	4119 (1820 to 9456)	0.09 (0.04 to 0.14)	<0.001
South Sudan	5989 (2613 to 13865)	4185 (1786 to 9709)	7503 (3175 to 17325)	4181 (1739 to 9697)	0 (-0.03 to 0.03)	0.955
Spain	228084 (103602 to 499241)	4801 (2154 to 10532)	522704 (233367 to 1105488)	4348 (1967 to 9201)	-0.33 (-0.39 to -0.26)	<0.001
Sri Lanka	22702 (10908 to 49426)	3712 (1737 to 8199)	72225 (33791 to 155868)	3668 (1695 to 7945)	-0.04 (-0.11 to 0.04)	0.301
Sudan	25723 (12250 to 59414)	4885 (2268 to 11315)	50739 (23977 to 111056)	4505 (2105 to 9860)	-0.27 (-0.28 to -0.25)	<0.001
Suriname	635 (304 to 1378)	3371 (1619 to 7305)	1600 (776 to 3407)	3263 (1580 to 6949)	-0.09 (-0.16 to -0.01)	0.022
Sweden	73132 (35355 to 155626)	4664 (2241 to 9937)	110613 (52666 to 228274)	4340 (2079 to 8949)	-0.22 (-0.29 to -0.16)	<0.001
Switzerland	49084 (22794 to 106451)	4666 (2156 to 10122)	95125 (43498 to 198393)	4439 (2058 to 9244)	-0.16 (-0.26 to -0.06)	0.002
Syrian Arab Republic	15989 (7501 to 36316)	4939 (2314 to 11229)	33556 (15862 to 75266)	4617 (2121 to 10534)	-0.22 (-0.26 to -0.19)	<0.001
Taiwan (Province of China)	29792 (13572 to 67477)	3588 (1588 to 8104)	154908 (70549 to 330346)	3806 (1746 to 8115)	0.26 (0.16 to 0.36)	<0.001
Tajikistan	7656 (3539 to 16707)	3993 (1831 to 8748)	11337 (5128 to 25706)	3972 (1780 to 8989)	-0.01 (-0.05 to 0.03)	0.624

Thailand	82434 (36898 to 184927)	4013 (1773 to 8988)	378883 (174070 to 812671)	3921 (1808 to 8397)	-0.09 (-0.14 to -0.04)	<0.001
Timor-Leste	464 (224 to 1017)	3804 (1819 to 8372)	2100 (981 to 4784)	3991 (1810 to 9199)	0.15 (0.11 to 0.18)	<0.001
Togo	1978 (866 to 4576)	3277 (1391 to 7647)	5689 (2432 to 13529)	3445 (1428 to 8267)	0.15 (0.1 to 0.19)	<0.001
Tokelau	5 (2 to 11)	4946 (2067 to 11890)	6 (3 to 13)	4505 (1965 to 10281)	-0.3 (-0.36 to -0.24)	<0.001
Tonga	154 (70 to 354)	4722 (2093 to 10810)	292 (130 to 644)	4582 (2026 to 10111)	-0.09 (-0.15 to -0.04)	0.001
Trinidad and Tobago	1907 (937 to 4165)	3314 (1573 to 7392)	5071 (2452 to 10956)	3169 (1533 to 6832)	-0.15 (-0.19 to -0.1)	<0.001
Tunisia	14967 (7078 to 33032)	5393 (2476 to 11936)	48181 (22556 to 105306)	4972 (2308 to 10859)	-0.27 (-0.3 to -0.23)	<0.001
Turkey	120398 (55673 to 263808)	5666 (2590 to 12427)	357385 (168342 to 788149)	5048 (2364 to 11147)	-0.35 (-0.46 to -0.24)	<0.001
Turkmenistan	4561 (2134 to 10173)	4029 (1860 to 9003)	9485 (4419 to 20971)	3798 (1782 to 8391)	-0.19 (-0.25 to -0.13)	<0.001
Tuvalu	15 (7 to 35)	4847 (2125 to 11651)	28 (13 to 66)	4622 (2024 to 10855)	-0.15 (-0.17 to -0.14)	<0.001
Uganda	13562 (6167 to 29913)	4036 (1771 to 9014)	35034 (14928 to 83166)	4646 (1944 to 10972)	0.46 (0.41 to 0.51)	<0.001
Ukraine	216973 (102583 to 483207)	4119 (1907 to 9251)	288620 (132318 to 639507)	4029 (1837 to 8958)	-0.07 (-0.09 to -0.06)	<0.001

United Arab Emirates	792 (357 to 1788)	5199 (2295 to 11663)	2741 (1266 to 6016)	4376 (1941 to 9789)	-0.57 (-0.75 to -0.39)	<0.001
United Kingdom	366395 (169286 to 810470)	4266 (1950 to 9452)	585897 (267732 to 1269286)	4077 (1870 to 8837)	-0.15 (-0.19 to -0.11)	<0.001
United Republic of Tanzania	24880 (10919 to 57950)	4573 (1949 to 10504)	64434 (27428 to 152810)	4475 (1874 to 10536)	-0.07 (-0.09 to -0.04)	<0.001
United States Virgin Islands	165 (79 to 362)	5405 (2475 to 11778)	501 (246 to 1054)	5190 (2352 to 11209)	-0.35 (-0.37 to -0.34)	<0.001
United States of America	1658591 (762393 to 3616495)	3405 (1585 to 7575)	3061591 (1379915 to 6611803)	3048 (1464 to 6501)	-0.14 (-0.16 to -0.12)	<0.001
Uruguay	13196 (6212 to 29286)	3913 (1821 to 8706)	24582 (11139 to 53578)	3900 (1786 to 8493)	-0.03 (-0.04 to -0.02)	<0.001
Uzbekistan	33349 (15496 to 73709)	3855 (1769 to 8567)	53251 (24893 to 119539)	3722 (1726 to 8335)	-0.12 (-0.17 to -0.08)	<0.001
Vanuatu	107 (48 to 243)	4192 (1834 to 9714)	326 (147 to 768)	4183 (1832 to 9953)	-0.01 (-0.05 to 0.04)	0.741
Venezuela (Bolivarian Republic of)	27312 (13212 to 58664)	4201 (2013 to 9036)	99017 (47578 to 206911)	4127 (1988 to 8617)	-0.05 (-0.14 to 0.04)	0.282
Viet Nam	127673 (58180 to 282017)	4545 (2052 to 10050)	310321 (135113 to 716625)	4788 (2073 to 11035)	0.17 (0.15 to 0.2)	<0.001
Yemen	12260 (5767 to 28044)	5344 (2468 to 12146)	36716 (17171 to 84174)	4974 (2280 to 11496)	-0.23 (-0.26 to -0.19)	<0.001
Zambia	5540 (2515 to 12571)	4019 (1771 to 9244)	13950 (6129 to 32316)	4379 (1880 to 10139)	0.28 (0.25 to 0.31)	<0.001

Zimbabwe	9178 (4060 to 20578)	4440 (1921 to 10025)	12955 (5695 to 30255)	4222 (1823 to 9879)	-0.15 (-0.2 to -0.1)	<0.001

Note: Estimates are for individuals aged over 65 years. AAPCs=average annual percent changes. CI=confidence interval. P value for the significant test of AAPCs. Numbers in parentheses are 95% uncertainty intervals (Cases and age-standardized rate) and 95% confidence interval (AAPCs).

Table S10. Sociodemographic-index-related inequalities in prevalence, deaths, and DALYs for ADOD, aged ≥65 years, both sexes

Region	Year	Inequality metrics	Prevalence (95% CI)	Deaths (95% CI)	DALYs (95% CI)
Global	1990	slope index of inequality	1366.63 (852.54 to 1880.72)	33.75 (9.35 to 58.15)	614.81 (240.06 to 989.56)
Global	2021	slope index of inequality	962.2 (547.37 to 1377.03)	10.17 (-10.94 to 31.28)	233.53 (-72.4 to 539.45)
Global	1990	concentration index	0.06 (0.05 to 0.07)	0.06 (0.04 to 0.08)	0.05 (0.04 to 0.07)
Global	2021	concentration index	0.06 (0.04 to 0.08)	0.05 (0.04 to 0.07)	0.05 (0.04 to 0.07)
High SDI	1990	slope index of inequality	834.11 (-47.96 to 1716.19)	33.2 (-10.18 to 76.57)	487.44 (-199.89 to 1174.76)
High SDI	2021	slope index of inequality	390.53 (-288.34 to 1069.4)	36.41 (3.99 to 68.83)	398.49 (-90.97 to 887.95)
High SDI	1990	concentration index	0.03 (0.01 to 0.05)	0.03 (0.01 to 0.05)	0.03 (0.01 to 0.05)
High SDI	2021	concentration index	0.04 (0.02 to 0.06)	0.03 (0.01 to 0.05)	0.03 (0.01 to 0.05)
High-middle SDI	1990	slope index of inequality	-750.11 (-1758.66 to 258.43)	-59.25 (-136.77 to 18.28)	-988.69 (-2041.14 to 63.75)
High-middle SDI	2021	slope index of inequality	-374.64 (-1240.46 to 491.17)	-17.77 (-74.21 to 38.68)	-357.36 (-1176.48 to 461.75)
High-middle SDI	1990	concentration index	-0.01 (-0.01 to 0)	-0.06 (-0.08 to -0.04)	-0.05 (-0.06 to -0.03)
High-middle SDI	2021	concentration index	-0.06 (-0.07 to -0.05)	-0.05 (-0.07 to -0.04)	-0.05 (-0.06 to -0.04)
Middle SDI	1990	slope index of inequality	-1132.81 (-1933.48 to -332.13)	-52.69 (-98.46 to -6.92)	-879.69 (-1574.46 to -184.93)
Middle SDI	2021	slope index of inequality	-240.47 (-965.38 to 484.44)	-28.79 (-71.83 to 14.25)	-389.31 (-1045.58 to 266.96)
Middle SDI	1990	concentration index	-0.04 (-0.06 to -0.02)	-0.04 (-0.08 to 0)	-0.04 (-0.07 to -0.01)
Middle SDI	2021	concentration index	-0.02 (-0.05 to 0)	-0.05 (-0.08 to -0.02)	-0.04 (-0.07 to -0.01)
Low-middle SDI	1990	slope index of inequality	1096.19 (262.19 to 1930.19)	51.68 (19.02 to 84.35)	793.8 (280.65 to 1306.95)
Low-middle SDI	2021	slope index of inequality	886.15 (-65.74 to 1838.04)	8.18 (-28.22 to 44.59)	285.86 (-316.31 to 888.03)

Low-middle SDI	1990	concentration index	0.03 (0 to 0.07)	0.02 (-0.02 to 0.06)	0.03 (-0.01 to 0.07)
Low-middle SDI	2021	concentration index	0 (-0.03 to 0.04)	-0.02 (-0.04 to 0)	-0.01 (-0.03 to 0.01)
Low SDI	1990	slope index of inequality	1130.28 (-243.11 to 2503.66)	-0.37 (-60.37 to 59.62)	275.89 (-716.64 to 1268.42)
Low SDI	2021	slope index of inequality	1299.79 (-11.13 to 2610.71)	16.72 (-53.88 to 87.32)	476.71 (-599.48 to 1552.9)
Low SDI	1990	concentration index	0.03 (0 to 0.07)	0.01 (-0.04 to 0.05)	0.01 (-0.03 to 0.06)
Low SDI	2021	concentration index	0.03 (-0.01 to 0.07)	0 (-0.05 to 0.04)	0 (-0.04 to 0.05)
Eastern Sub-Saharan Africa	1990	slope index of inequality	-158.17 (-473.19 to 156.86)	-8.1 (-51.34 to 35.14)	-133.67 (-670.22 to 402.89)
Eastern Sub-Saharan Africa	2021	slope index of inequality	2.59 (-286.26 to 291.43)	25.41 (-22.21 to 73.03)	257.39 (-296.7 to 811.48)
Eastern Sub-Saharan Africa	1990	concentration index	-0.01 (-0.01 to 0)	-0.03 (-0.05 to 0)	-0.02 (-0.04 to -0.01)
Eastern Sub-Saharan Africa	2021	concentration index	0 (-0.01 to 0.01)	0 (-0.03 to 0.03)	0 (-0.02 to 0.02)
Western Sub-Saharan Africa	1990	slope index of inequality	-335.43 (-597.33 to -73.53)	-12.82 (-40.26 to 14.61)	-260.76 (-598.18 to 76.65)
Western Sub-Saharan Africa	2021	slope index of inequality	-191.09 (-352.32 to -29.86)	-11.88 (-34.01 to 10.25)	-196.84 (-465.25 to 71.58)
Western Sub-Saharan Africa	1990	concentration index	-0.03 (-0.03 to -0.02)	-0.03 (-0.04 to -0.01)	-0.03 (-0.04 to -0.01)
Western Sub-Saharan Africa	2021	concentration index	-0.03 (-0.04 to -0.02)	-0.01 (-0.02 to 0.01)	-0.01 (-0.02 to 0)
Central Sub-Saharan Africa	1990	slope index of inequality	-384.92 (-724.79 to -45.05)	56.41 (-3.24 to 116.06)	550.89 (-201.4 to 1303.19)
Central Sub-Saharan Africa	2021	slope index of inequality	-740.39 (-1077.85 to -402.93)	14.46 (-19.74 to 48.66)	-75.71 (-433.63 to 282.22)
Central Sub-Saharan Africa	1990	concentration index	-0.01 (-0.01 to 0)	0.02 (-0.01 to 0.04)	0.01 (-0.01 to 0.02)
Central Sub-Saharan Africa	2021	concentration index	-0.01 (-0.01 to -0.01)	-0.01 (-0.02 to 0.01)	-0.01 (-0.02 to 0)
North Africa and Middle East	1990	slope index of inequality	-106.73 (-578.85 to 365.38)	23.31 (0.43 to 46.18)	136.74 (-136.54 to 410.03)
North Africa and Middle East	2021	slope index of inequality	-1.59 (-362.67 to 359.49)	-1.24 (-19.98 to 17.5)	-119.66 (-364.83 to 125.51)
North Africa and Middle East	1990	concentration index	0.01 (0 to 0.02)	0.01 (-0.01 to 0.03)	0.01 (-0.01 to 0.02)
North Africa and Middle East	2021	concentration index	0.01 (0 to 0.02)	0 (-0.01 to 0.02)	0 (-0.01 to 0.02)

Oceania	1990	slope index of inequality	-447.31 (-1002.21 to 107.58)	25.93 (-15.6 to 67.47)	163.47 (-350.07 to 677.01)
Oceania	2021	slope index of inequality	-578.75 (-1346.43 to 188.94)	-5 (-92.62 to 82.61)	-304.02 (-1475.58 to 867.54)
Oceania	1990	concentration index	-0.01 (-0.02 to -0.01)	0.03 (0.01 to 0.04)	0.01 (0 to 0.02)
Oceania	2021	concentration index	0 (-0.01 to 0)	0.02 (0 to 0.04)	0.01 (0 to 0.02)
South Asia	1990	slope index of inequality	-577.27 (-851.24 to -303.3)	-40.55 (-41.65 to -39.45)	-569.74 (-1083.44 to -56.03)
South Asia	2021	slope index of inequality	-179.21 (-589.2 to 230.78)	-24.6 (-87.4 to 38.2)	-310.32 (-964.68 to 344.05)
South Asia	1990	concentration index	-0.01 (-0.02 to -0.01)	-0.03 (-0.05 to -0.01)	-0.02 (-0.04 to -0.01)
South Asia	2021	concentration index	0 (-0.01 to 0)	-0.01 (-0.02 to 0)	-0.01 (-0.02 to 0)
Caribbean	1990	slope index of inequality	-172.66 (-400.13 to 54.82)	-3.94 (-14.89 to 7.01)	-94.31 (-233.09 to 44.47)
Caribbean	2021	slope index of inequality	-58.26 (-312.35 to 195.83)	-3.53 (-13.37 to 6.31)	-43.12 (-206.51 to 120.28)
Caribbean	1990	concentration index	-0.01 (-0.03 to 0.01)	-0.01 (-0.01 to 0)	-0.01 (-0.02 to 0)
Caribbean	2021	concentration index	0 (-0.01 to 0.01)	-0.01 (-0.01 to 0)	0 (-0.01 to 0)
Southeast Asia	1990	slope index of inequality	-238.11 (-608.86 to 132.64)	23.38 (-10.61 to 57.36)	186.45 (-297.71 to 670.61)
Southeast Asia	2021	slope index of inequality	-143.34 (-255.72 to -30.96)	-16.32 (-55.84 to 23.21)	-258.53 (-718.02 to 200.95)
Southeast Asia	1990	concentration index	-0.02 (-0.03 to 0)	-0.01 (-0.05 to 0.04)	-0.01 (-0.04 to 0.02)
Southeast Asia	2021	concentration index	-0.01 (-0.02 to 0)	-0.03 (-0.06 to 0)	-0.02 (-0.04 to 0)
Southern Sub-Saharan Africa	1990	slope index of inequality	445.14 (-37.33 to 927.6)	-37.86 (-106.57 to 30.85)	-475.72 (-1073.28 to 121.84)
Southern Sub-Saharan Africa	2021	slope index of inequality	271.51 (-254.36 to 797.39)	-14.42 (-44.65 to 15.81)	-330.05 (-818.53 to 158.42)
Southern Sub-Saharan Africa	1990	concentration index	0.01 (0 to 0.01)	-0.02 (-0.03 to -0.01)	-0.01 (-0.02 to -0.01)
Southern Sub-Saharan Africa	2021	concentration index	0 (0 to 0.01)	0 (-0.01 to 0)	0 (-0.01 to 0)
Central Latin America	1990	slope index of inequality	-60.36 (-454.95 to 334.22)	-7.17 (-32.5 to 18.16)	-104.12 (-396.53 to 188.3)

Central Latin America	2021	slope index of inequality	-270.9 (-491.81 to -49.99)	-17.3 (-53.45 to 18.85)	-321.08 (-897.33 to 255.16)
Central Latin America	1990	concentration index	0 (-0.03 to 0.03)	0 (-0.05 to 0.05)	0 (-0.05 to 0.04)
Central Latin America	2021	concentration index	-0.05 (-0.08 to -0.02)	-0.06 (-0.09 to -0.03)	-0.06 (-0.09 to -0.03)
Central Asia	1990	slope index of inequality	129.14 (-76.92 to 335.2)	-2.88 (-20.37 to 14.6)	13.03 (-241.96 to 268.02)
Central Asia	2021	slope index of inequality	208.39 (-320.24 to 737.01)	-4.98 (-28.26 to 18.29)	-11.39 (-330.73 to 307.95)
Central Asia	1990	concentration index	0.01 (0 to 0.02)	0 (-0.01 to 0.01)	0.01 (0 to 0.01)
Central Asia	2021	concentration index	0.01 (0 to 0.02)	0 (-0.02 to 0.02)	0 (-0.01 to 0.02)
East Asia	1990	slope index of inequality	-2429.15 (-6541.52 to 1683.23)	-212.15 (-312.51 to -111.79)	-3040.02 (-5322.16 to -757.88)
East Asia	2021	slope index of inequality	-692.06 (-7822.39 to 6438.27)	-33.25 (-239.59 to 173.09)	-599.2 (-4422.31 to 3223.91)
East Asia	1990	concentration index	-0.01 (-0.01 to 0)	-0.01 (-0.01 to -0.01)	-0.01 (-0.01 to -0.01)
East Asia	2021	concentration index	0 (-0.02 to 0.02)	0 (-0.02 to 0.01)	0 (-0.02 to 0.01)
Andean Latin America	1990	slope index of inequality	30.55 (-713.46 to 774.56)	-12.21 (-31.48 to 7.05)	-149.94 (-534.01 to 234.12)
Andean Latin America	2021	slope index of inequality	-359.94 (-554.14 to -165.74)	-25.66 (-65.44 to 14.11)	-367.54 (-847.38 to 112.31)
Andean Latin America	1990	concentration index	0 (-0.02 to 0.03)	-0.01 (-0.03 to 0.01)	0 (-0.03 to 0.02)
Andean Latin America	2021	concentration index	-0.01 (-0.02 to -0.01)	-0.02 (-0.05 to 0.01)	-0.01 (-0.03 to 0.01)
Central Europe	1990	slope index of inequality	76.72 (-117.3 to 270.74)	1.91 (-7.96 to 11.77)	26.61 (-111.07 to 164.29)
Central Europe	2021	slope index of inequality	-55.99 (-124.71 to 12.73)	10.51 (3.81 to 17.2)	91.53 (0.28 to 182.77)
Central Europe	1990	concentration index	0 (-0.01 to 0.01)	0 (-0.01 to 0.01)	0 (-0.01 to 0.01)
Central Europe	2021	concentration index	0 (0 to 0)	0.01 (0 to 0.02)	0.01 (0 to 0.01)
Eastern Europe	1990	slope index of inequality	31.08 (-244.93 to 307.08)	-0.99 (-9.6 to 7.61)	5.13 (-135.07 to 145.33)
Eastern Europe	2021	slope index of inequality	50.36 (-160.18 to 260.9)	16.33 (1.58 to 31.09)	137.62 (-38 to 313.24)
Eastern Europe	1990	concentration index	0 (0 to 0)	0 (0 to 0.01)	0 (0 to 0.01)

Eastern Europe	2021	concentration index	0 (0 to 0.01)	0 (0 to 0.01)	0 (0 to 0.01)
Southern Latin America	1990	slope index of inequality	25.96 (-675.28 to 727.19)	10.53 (-38.91 to 59.98)	115.03 (-502.98 to 733.04)
Southern Latin America	2021	slope index of inequality	53.58 (-84.03 to 191.19)	-23.76 (-29.96 to -17.55)	-246.53 (-323.4 to -169.65)
Southern Latin America	1990	concentration index	0 (-0.01 to 0.01)	0.01 (-0.01 to 0.03)	0.01 (-0.01 to 0.02)
Southern Latin America	2021	concentration index	0 (0 to 0.01)	-0.01 (-0.02 to -0.01)	-0.01 (-0.01 to 0)
Western Europe	1990	slope index of inequality	375.53 (-104.88 to 855.93)	-8.28 (-51.72 to 35.16)	-50.33 (-625.55 to 524.89)
Western Europe	2021	slope index of inequality	-44.56 (-846.69 to 757.56)	3.91 (-28.36 to 36.18)	5.36 (-426.51 to 437.23)
Western Europe	1990	concentration index	0.04 (0.01 to 0.06)	0 (-0.02 to 0.03)	0.01 (-0.01 to 0.04)
Western Europe	2021	concentration index	0.04 (0 to 0.07)	0.01 (-0.01 to 0.04)	0.02 (-0.01 to 0.05)
High-income North America	1990	slope index of inequality	752.88 (-155.45 to 1661.21)	-53.15 (-190.26 to 83.96)	-440.42 (-1926.82 to 1045.98)
High-income North America	2021	slope index of inequality	-184 (-622.02 to 254.02)	-55.53 (-202.58 to 91.52)	-683.12 (-2286.56 to 920.33)
High-income North America	1990	concentration index	0.01 (0.01 to 0.01)	-0.02 (-0.02 to -0.02)	-0.01 (-0.01 to -0.01)
High-income North America	2021	concentration index	0 (0 to 0)	-0.03 (-0.03 to -0.03)	-0.02 (-0.02 to -0.02)
High-income Asia Pacific	1990	slope index of inequality	1291.73 (-3528.95 to 6112.41)	102.56 (-190.37 to 395.49)	1518.47 (-2422.18 to 5459.12)
High-income Asia Pacific	2021	slope index of inequality	2024.08 (1247.79 to 2800.38)	105.3 (20.26 to 190.33)	1623.17 (550.91 to 2695.43)
High-income Asia Pacific	1990	concentration index	-0.02 (-0.03 to 0)	-0.03 (-0.06 to 0)	-0.03 (-0.05 to 0)
High-income Asia Pacific	2021	concentration index	0.02 (0.01 to 0.03)	0.02 (0 to 0.04)	0.02 (0.01 to 0.03)
Sub-Saharan Africa	1990	slope index of inequality	848.47 (-322.22 to 2019.16)	17.76 (-30.79 to 66.32)	421.3 (-421.74 to 1264.34)
Sub-Saharan Africa	2021	slope index of inequality	1032.82 (-90.82 to 2156.45)	34.86 (-24.66 to 94.37)	643.6 (-271.71 to 1558.91)
Sub-Saharan Africa	1990	concentration index	-0.01 (-0.04 to 0.03)	-0.03 (-0.06 to 0)	-0.03 (-0.06 to 0.01)
Sub-Saharan Africa	2021	concentration index	-0.01 (-0.05 to 0.02)	-0.03 (-0.07 to 0.01)	-0.03 (-0.07 to 0.01)
Southeast Asia, east Asia, and Oceania	1990	slope index of inequality	-320.95 (-546.45 to -95.45)	25.71 (-0.62 to 52.03)	213.81 (-119.65 to 547.27)

Southeast Asia, east Asia, and Oceania	2021	slope index of inequality	-224.28 (-376.5 to -72.07)	-16.13 (-39.76 to 7.5)	-265.8 (-551.9 to 20.3)
Southeast Asia, east Asia, and Oceania	1990	concentration index	-0.01 (-0.01 to 0)	0.02 (-0.01 to 0.04)	0.01 (-0.01 to 0.03)
Southeast Asia, east Asia, and Oceania	2021	concentration index	0.04 (0.03 to 0.06)	0.04 (0.02 to 0.05)	0.04 (0.03 to 0.05)
Latin America and Caribbean	1990	slope index of inequality	-342.11 (-825.14 to 140.93)	-9.4 (-27.98 to 9.18)	-157.1 (-453.16 to 138.96)
Latin America and Caribbean	2021	slope index of inequality	-474.92 (-941.79 to -8.05)	-12.32 (-29.38 to 4.75)	-233.9 (-574.61 to 106.81)
Latin America and Caribbean	1990	concentration index	-0.03 (-0.06 to -0.01)	-0.05 (-0.09 to 0)	-0.05 (-0.08 to -0.01)
Latin America and Caribbean	2021	concentration index	-0.05 (-0.08 to -0.03)	-0.08 (-0.12 to -0.03)	-0.07 (-0.11 to -0.04)
Central Europe, eastern Europe, and central Asia	1990	slope index of inequality	115.36 (-27.75 to 258.47)	-1.34 (-8.57 to 5.88)	-4.28 (-109.51 to 100.95)
Central Europe, eastern Europe, and central Asia	2021	slope index of inequality	70.88 (-68.31 to 210.06)	9 (0.7 to 17.31)	102.89 (-3.61 to 209.38)
Central Europe, eastern Europe, and central Asia	1990	concentration index	0 (-0.01 to 0.01)	0 (-0.01 to 0.01)	0 (-0.01 to 0.01)
Central Europe, eastern Europe, and central Asia	2021	concentration index	0 (0 to 0)	0.01 (0 to 0.02)	0.01 (0 to 0.01)
High income	1990	slope index of inequality	585.69 (-179.45 to 1350.82)	-0.71 (-36.37 to 34.94)	56.2 (-422.65 to 535.05)
High income	2021	slope index of inequality	330.92 (-397.32 to 1059.15)	7.57 (-20.64 to 35.77)	77.73 (-354.83 to 510.28)
High income	1990	concentration index	0.03 (0 to 0.05)	0.01 (-0.02 to 0.03)	0.01 (-0.01 to 0.03)
High income	2021	concentration index	0.03 (0.01 to 0.05)	0.01 (-0.01 to 0.04)	0.02 (0 to 0.04)

Note: CI=confidence interval; SDI=sociodemographic index; ADOD= Alzheimer's disease and other dementias; DALYs= disease-adjusted life years.

Table S11. Case number and ASR prediction of prevalence for ADOD aged ≥65 years from 2022 to 2050 by BAPC models in global, by both sexes

Year	Sex	Number (000000s)	ASR (per 100,000)	Crude rate (per 100,000)
2021	Both	49.1 (49.03 to 49.17)	6833.22 (6823.52 to 6842.93)	6374.65 (6365.63 to 6383.66)
2022	Both	51.69 (50.57 to 52.81)	6858.91 (6710.39 to 7007.42)	6470.42 (6329.91 to 6610.92)
2023	Both	53.97 (52.42 to 55.51)	6935.3 (6737.07 to 7133.53)	6555.34 (6367.42 to 6743.25)
2024	Both	56.48 (54.31 to 58.65)	7012.82 (6744.15 to 7281.5)	6663.78 (6407.9 to 6919.66)
2025	Both	59.18 (56.2 to 62.15)	7092.97 (6737.15 to 7448.79)	6783.02 (6442.26 to 7123.78)
2026	Both	62 (58.04 to 65.96)	7176.81 (6719 to 7634.62)	6903.61 (6462.88 to 7344.34)
2027	Both	64.89 (59.76 to 70.02)	7261.7 (6687.78 to 7835.63)	7015.21 (6460.51 to 7569.91)
2028	Both	68 (61.49 to 74.5)	7348.4 (6645.61 to 8051.19)	7125.92 (6444.14 to 7807.69)
2029	Both	71.43 (63.32 to 79.54)	7437.49 (6593.78 to 8281.2)	7245.19 (6422.99 to 8067.39)
2030	Both	75.11 (65.16 to 85.05)	7530.12 (6533.48 to 8526.77)	7371.48 (6395.53 to 8347.43)
2031	Both	78.95 (66.92 to 90.98)	7626.77 (6464.63 to 8788.91)	7503.92 (6360.31 to 8647.52)
2032	Both	82.86 (68.48 to 97.24)	7724.68 (6384.05 to 9065.31)	7639.43 (6313.7 to 8965.15)
2033	Both	86.97 (69.95 to 103.99)	7825.02 (6292.9 to 9357.14)	7785.89 (6262.06 to 9309.72)
2034	Both	91.41 (71.4 to 111.41)	7928.86 (6191.99 to 9665.73)	7947.41 (6207.91 to 9686.91)
2035	Both	96.09 (72.74 to 119.44)	8037.32 (6081.83 to 9992.82)	8120.74 (6147.5 to 10093.97)
2036	Both	100.94 (73.88 to 128)	8150.55 (5961.67 to 10339.44)	8302.79 (6077.06 to 10528.51)
2037	Both	105.83 (74.7 to 136.97)	8266.16 (5828.63 to 10703.7)	8488.93 (5991.62 to 10986.24)
2038	Both	110.91 (75.28 to 146.55)	8385.24 (5683.08 to 11087.39)	8688.21 (5896.81 to 11479.62)
2039	Both	116.31 (75.68 to 156.94)	8508.86 (5525.21 to 11492.52)	8905.92 (5794.66 to 12017.18)
2040	Both	121.95 (75.81 to 168.1)	8638.07 (5354.89 to 11921.25)	9136.63 (5679.53 to 12593.73)
2041	Both	127.78 (75.59 to 179.97)	8772.97 (5171.04 to 12374.91)	9374.71 (5545.9 to 13203.52)
2042	Both	133.7 (74.93 to 192.46)	8912.1 (4971.37 to 12852.82)	9613.42 (5387.89 to 13838.96)
2043	Both	139.83 (73.87 to 205.79)	9056.2 (4755.31 to 13357.09)	9862.71 (5210.13 to 14515.29)
2044	Both	146.3 (72.42 to 220.18)	9206.12 (4522.14 to 13890.1)	10129.67 (5014.42 to 15244.91)
2045	Both	153.06 (70.51 to 235.61)	9362.75 (4270.94 to 14454.55)	10406.92 (4794.24 to 16019.6)
2046	Both	160.06 (68.05 to 252.07)	9526.42 (4000.22 to 15052.62)	10686.98 (4543.7 to 16830.26)
2047	Both	167.22 (64.94 to 269.49)	9696.76 (3707.92 to 15685.61)	10961.7 (4257.35 to 17666.05)

2048	Both	174.68 (61.2 to 288.17)	9874.32 (3392.41 to 16356.22)	11235.09 (3935.92 to 18534.27)
2049	Both	182.6 (56.78 to 308.41)	10059.72 (3051.91 to 17067.54)	11515.41 (3580.95 to 19449.86)
2050	Both	190.88 (51.59 to 330.17)	10253.79 (2684.38 to 17823.2)	11802.94 (3189.97 to 20415.91)
2021	Male	17.28 (17.25 to 17.31)	5744.15 (5741.38 to 5746.92)	4948.75 (4940.38 to 4957.11)
2022	Male	18.35 (17.95 to 18.75)	5765.28 (5686.36 to 5844.21)	5071.37 (4961.15 to 5181.59)
2023	Male	19.17 (18.62 to 19.72)	5822.61 (5692.86 to 5952.36)	5142.5 (4994.99 to 5290.01)
2024	Male	20.08 (19.31 to 20.85)	5881.44 (5687.19 to 6075.69)	5233.73 (5032.74 to 5434.72)
2025	Male	21.06 (20 to 22.11)	5942.44 (5672.95 to 6211.93)	5333.39 (5065.52 to 5601.25)
2026	Male	22.07 (20.66 to 23.48)	6005.99 (5651.26 to 6360.72)	5432.79 (5086.09 to 5779.49)
2027	Male	23.11 (21.28 to 24.93)	6069.98 (5620.12 to 6519.83)	5523.38 (5086.77 to 5959.99)
2028	Male	24.22 (21.9 to 26.53)	6135.29 (5581.19 to 6689.39)	5612.78 (5075.83 to 6149.72)
2029	Male	25.45 (22.56 to 28.34)	6202.67 (5535.55 to 6869.79)	5709.25 (5061.29 to 6357.21)
2030	Male	26.77 (23.23 to 30.32)	6272.83 (5483.89 to 7061.78)	5810.68 (5041.14 to 6580.22)
2031	Male	28.15 (23.86 to 32.44)	6345.97 (5425.99 to 7265.95)	5915.86 (5013.82 to 6817.91)
2032	Male	29.54 (24.41 to 34.67)	6419.88 (5359.42 to 7480.35)	6022.4 (4976.55 to 7068.26)
2033	Male	31.01 (24.93 to 37.08)	6495.33 (5285.03 to 7705.63)	6136.65 (4934.53 to 7338.77)
2034	Male	32.59 (25.45 to 39.73)	6573.17 (5203.55 to 7942.8)	6262.29 (4890.16 to 7634.42)
2035	Male	34.26 (25.93 to 42.6)	6654.11 (5115.29 to 8192.93)	6396.91 (4840.62 to 7953.21)
2036	Male	35.99 (26.33 to 45.65)	6738.13 (5019.7 to 8456.57)	6538.57 (4783.34 to 8293.79)
2037	Male	37.74 (26.62 to 48.86)	6823.21 (4914.6 to 8731.82)	6684.42 (4715 to 8653.85)
2038	Male	39.56 (26.83 to 52.28)	6909.89 (4800.35 to 9019.43)	6841.74 (4640.06 to 9043.41)
2039	Male	41.49 (26.97 to 56.01)	6998.85 (4677.24 to 9320.46)	7014.49 (4559.94 to 9469.04)
2040	Male	43.52 (27.02 to 60.01)	7090.67 (4545.32 to 9636.02)	7198.36 (4470.03 to 9926.69)
2041	Male	45.62 (26.95 to 64.28)	7185.21 (4403.95 to 9966.48)	7389.13 (4366.03 to 10412.23)
2042	Male	47.76 (26.73 to 68.79)	7281.05 (4251.63 to 10310.46)	7581.9 (4243.45 to 10920.35)
2043	Male	49.98 (26.36 to 73.61)	7378.42 (4088.32 to 10668.51)	7784.67 (4105.91 to 11463.42)
2044	Male	52.35 (25.87 to 78.82)	7477.69 (3913.99 to 11041.38)	8002.94 (3954.64 to 12051.24)
2045	Male	54.82 (25.2 to 84.44)	7579.13 (3728.44 to 11429.81)	8230.41 (3784.04 to 12676.78)
2046	Male	57.39 (24.34 to 90.43)	7682.64 (3531.19 to 11834.1)	8460.88 (3589.17 to 13332.58)
2047	Male	60.02 (23.25 to 96.79)	7787.54 (3321.44 to 12253.64)	8687.85 (3365.59 to 14010.11)
2048	Male	62.78 (21.93 to 103.63)	7893.94 (3099.01 to 12688.87)	8914.51 (3113.74 to 14715.27)

2049	Male	65.71 (20.36 to 111.06)	8001.99 (2863.67 to 13140.31)	9147.42 (2834.82 to 15460.02)
2050	Male	68.78 (18.52 to 119.05)	8111.83 (2615.17 to 13608.5)	9386.7 (2526.67 to 16246.73)
2021	Female	31.81 (31.77 to 31.85)	7603.06 (7600.41 to 7605.7)	7557.68 (7548.13 to 7567.24)
2022	Female	33.34 (32.61 to 34.06)	7643.34 (7537.8 to 7748.87)	7629.01 (7463.43 to 7794.59)
2023	Female	34.79 (33.8 to 35.79)	7735.32 (7561.28 to 7909.36)	7724.74 (7503.38 to 7946.1)
2024	Female	36.4 (35 to 37.8)	7828.69 (7567.51 to 8089.87)	7846.67 (7545.39 to 8147.95)
2025	Female	38.12 (36.2 to 40.04)	7924.26 (7561.29 to 8287.22)	7981.31 (7580.29 to 8382.33)
2026	Female	39.93 (37.38 to 42.48)	8022.73 (7544.32 to 8501.13)	8118.64 (7600.23 to 8637.04)
2027	Female	41.78 (38.48 to 45.09)	8120.52 (7513.19 to 8727.86)	8246.94 (7594.74 to 8899.14)
2028	Female	43.78 (39.59 to 47.97)	8219.07 (7470.29 to 8967.86)	8374.89 (7573.57 to 9176.21)
2029	Female	45.98 (40.76 to 51.2)	8319.29 (7417.01 to 9221.56)	8512.95 (7546.93 to 9478.97)
2030	Female	48.33 (41.94 to 54.73)	8421.84 (7354.08 to 9489.6)	8659.88 (7513.54 to 9806.22)
2031	Female	50.8 (43.06 to 58.54)	8526.75 (7281.1 to 9772.41)	8814.97 (7471.94 to 10158)
2032	Female	53.32 (44.07 to 62.57)	8630.38 (7194.28 to 10066.48)	8974.51 (7417.7 to 10531.31)
2033	Female	55.97 (45.02 to 66.91)	8734.52 (7095.52 to 10373.52)	9147.98 (7358.45 to 10937.51)
2034	Female	58.82 (45.95 to 71.68)	8840.57 (6986.07 to 10695.06)	9340.01 (7296.91 to 11383.1)
2035	Female	61.83 (46.82 to 76.84)	8949.32 (6866.36 to 11032.28)	9546.34 (7228.29 to 11864.39)
2036	Female	64.94 (47.55 to 82.34)	9060.46 (6735.53 to 11385.38)	9762.64 (7147.59 to 12377.68)
2037	Female	68.09 (48.08 to 88.11)	9170.87 (6590.51 to 11751.23)	9982.52 (7048.29 to 12916.76)
2038	Female	71.36 (48.45 to 94.26)	9282.03 (6432.45 to 12131.61)	10216.65 (6937.08 to 13496.21)
2039	Female	74.82 (48.71 to 100.93)	9395.19 (6262.09 to 12528.3)	10471.67 (6816.78 to 14126.56)
2040	Female	78.44 (48.79 to 108.09)	9511.06 (6079.56 to 12942.57)	10741.25 (6680.83 to 14801.67)
2041	Female	82.17 (48.64 to 115.69)	9629.28 (5884 to 13374.55)	11018.54 (6522.69 to 15514.39)
2042	Female	85.94 (48.2 to 123.68)	9747.78 (5673.45 to 13822.11)	11295.26 (6335.34 to 16255.18)
2043	Female	89.84 (47.5 to 132.18)	9867.37 (5448.27 to 14286.48)	11582.94 (6124.22 to 17041.66)
2044	Female	93.95 (46.56 to 141.35)	9988.8 (5208.64 to 14768.96)	11890.06 (5891.66 to 17888.47)
2045	Female	98.24 (45.31 to 151.18)	10112.5 (4954.44 to 15270.56)	12208.43 (5630.39 to 18786.46)
2046	Female	102.67 (43.71 to 161.64)	10238.3 (4685.08 to 15791.51)	12529.52 (5333.75 to 19725.28)
2047	Female	107.2 (41.69 to 172.7)	10365.26 (4399.6 to 16330.92)	12843.92 (4995.53 to 20692.3)
2048	Female	111.91 (39.27 to 184.54)	10493.7 (4097.88 to 16889.52)	13156.43 (4616.64 to 21696.23)
2049	Female	116.89 (36.42 to 197.36)	10623.92 (3779.76 to 17468.08)	13476.66 (4198.93 to 22754.39)

2050	Female	122.1 (33.07 to 211.12)	10756.13 (3444.98 to 18067.29)	13804.9 (3739.55 to 23870.25)

Note: Data in parentheses are 95% confidence intervals. Count data in 100000 are presented to two decimal places. ASR, age-standardized rate; BAPC, Bayesian age-period-cohort; ADOD, Alzheimer's disease and other dementias.

Table S12. Case number and ASR prediction of deaths for ADOD aged ≥65 years from 2022 to 2050 by BAPC models in global, by both sexes

Year	Sex	Number (000000s)	ASR (per 100,000)	Crude rate (per 100,000)
2021	Both	1.88 (1.87 to 1.89)	274.06 (272.16 to 275.95)	243.94 (242.23 to 245.65)
2022	Both	1.98 (1.93 to 2.02)	273.96 (267.8 to 280.12)	247.58 (242 to 253.16)
2023	Both	2.05 (1.99 to 2.11)	274.95 (267.16 to 282.75)	249.11 (242.02 to 256.19)
2024	Both	2.14 (2.06 to 2.22)	275.95 (265.78 to 286.12)	252.21 (242.89 to 261.52)
2025	Both	2.23 (2.12 to 2.34)	277.04 (263.91 to 290.17)	255.69 (243.55 to 267.83)
2026	Both	2.32 (2.19 to 2.46)	278.24 (261.64 to 294.83)	258.84 (243.38 to 274.3)
2027	Both	2.42 (2.24 to 2.59)	279.48 (258.96 to 300)	261.19 (242 to 280.39)
2028	Both	2.52 (2.29 to 2.74)	280.74 (255.91 to 305.57)	263.6 (240.27 to 286.94)
2029	Both	2.63 (2.35 to 2.9)	282.04 (252.55 to 311.52)	266.73 (238.83 to 294.64)
2030	Both	2.75 (2.42 to 3.09)	283.42 (248.95 to 317.89)	270.22 (237.33 to 303.11)
2031	Both	2.88 (2.48 to 3.28)	284.91 (245.1 to 324.72)	273.87 (235.58 to 312.16)
2032	Both	3.01 (2.53 to 3.49)	286.45 (240.94 to 331.95)	277.55 (233.44 to 321.65)
2033	Both	3.15 (2.59 to 3.71)	288.01 (236.49 to 339.54)	281.85 (231.42 to 332.28)
2034	Both	3.3 (2.64 to 3.96)	289.64 (231.77 to 347.51)	287.09 (229.74 to 344.45)
2035	Both	3.47 (2.7 to 4.23)	291.38 (226.82 to 355.94)	292.92 (228.05 to 357.78)
2036	Both	3.64 (2.75 to 4.52)	293.24 (221.63 to 364.86)	299.06 (226.09 to 372.02)
2037	Both	3.81 (2.79 to 4.82)	295.17 (216.13 to 374.21)	305.25 (223.62 to 386.89)
2038	Both	3.98 (2.82 to 5.15)	297.17 (210.32 to 384.02)	312.09 (221.05 to 403.13)
2039	Both	4.18 (2.85 to 5.5)	299.25 (204.21 to 394.29)	319.88 (218.54 to 421.23)
2040	Both	4.38 (2.88 to 5.88)	301.44 (197.81 to 405.08)	328.2 (215.71 to 440.69)
2041	Both	4.59 (2.89 to 6.29)	303.75 (191.08 to 416.42)	336.68 (212.26 to 461.1)
2042	Both	4.8 (2.89 to 6.7)	306.13 (183.98 to 428.29)	344.96 (207.92 to 482.01)
2043	Both	5.01 (2.88 to 7.15)	308.6 (176.49 to 440.71)	353.71 (203.05 to 504.37)
2044	Both	5.25 (2.86 to 7.64)	311.17 (168.61 to 453.72)	363.31 (197.83 to 528.8)

2045	Both	5.49 (2.82 to 8.16)	313.87 (160.33 to 467.4)	373.24 (191.85 to 554.62)
2046	Both	5.74 (2.77 to 8.71)	316.72 (151.63 to 481.8)	383.04 (184.84 to 581.25)
2047	Both	5.98 (2.69 to 9.28)	319.7 (142.45 to 496.94)	392.29 (176.53 to 608.05)
2048	Both	6.24 (2.6 to 9.88)	322.82 (132.77 to 512.87)	401.4 (167.14 to 635.66)
2049	Both	6.52 (2.49 to 10.54)	326.1 (122.56 to 529.64)	410.87 (156.83 to 664.9)
2050	Both	6.8 (2.35 to 11.25)	329.54 (111.77 to 547.32)	420.55 (145.47 to 695.62)
2021	Male	0.59 (0.59 to 0.6)	224.8 (224.22 to 225.38)	170.13 (168.6 to 171.66)
2022	Male	0.64 (0.62 to 0.65)	225.18 (221.89 to 228.48)	176.7 (172.5 to 180.9)
2023	Male	0.67 (0.65 to 0.69)	226.08 (220.99 to 231.17)	178.49 (173.2 to 183.78)
2024	Male	0.7 (0.67 to 0.72)	227 (219.63 to 234.37)	181.52 (174.59 to 188.44)
2025	Male	0.73 (0.69 to 0.77)	227.95 (217.93 to 237.97)	184.79 (175.78 to 193.8)
2026	Male	0.76 (0.72 to 0.81)	228.94 (215.93 to 241.94)	187.73 (176.26 to 199.21)
2027	Male	0.79 (0.74 to 0.85)	229.92 (213.62 to 246.22)	189.99 (175.73 to 204.24)
2028	Male	0.83 (0.75 to 0.9)	230.9 (211.03 to 250.77)	192.3 (174.94 to 209.66)
2029	Male	0.87 (0.78 to 0.96)	231.91 (208.22 to 255.6)	195.19 (174.39 to 215.98)
2030	Male	0.91 (0.8 to 1.03)	232.95 (205.2 to 260.71)	198.28 (173.72 to 222.84)
2031	Male	0.96 (0.82 to 1.09)	234.03 (201.97 to 266.1)	201.39 (172.75 to 230.03)
2032	Male	1 (0.84 to 1.16)	235.1 (198.48 to 271.73)	204.4 (171.37 to 237.43)
2033	Male	1.05 (0.86 to 1.24)	236.18 (194.76 to 277.61)	207.83 (170.02 to 245.63)
2034	Male	1.1 (0.88 to 1.33)	237.29 (190.84 to 283.74)	211.94 (168.9 to 254.97)
2035	Male	1.16 (0.9 to 1.42)	238.44 (186.74 to 290.13)	216.41 (167.71 to 265.11)
2036	Male	1.22 (0.92 to 1.52)	239.63 (182.44 to 296.82)	221.03 (166.24 to 275.82)
2037	Male	1.27 (0.93 to 1.62)	240.83 (177.9 to 303.76)	225.63 (164.33 to 286.94)
2038	Male	1.33 (0.94 to 1.73)	242.05 (173.15 to 310.96)	230.7 (162.34 to 299.07)
2039	Male	1.4 (0.95 to 1.85)	243.3 (168.18 to 318.42)	236.5 (160.38 to 312.61)
2040	Male	1.47 (0.96 to 1.98)	244.59 (163 to 326.17)	242.68 (158.19 to 327.18)
2041	Male	1.54 (0.96 to 2.11)	245.9 (157.6 to 334.2)	248.99 (155.52 to 342.46)
2042	Male	1.61 (0.96 to 2.26)	247.22 (151.95 to 342.5)	255.19 (152.2 to 358.18)

2043	Male	1.68 (0.95 to 2.41)	248.56 (146.04 to 351.08)	261.8 (148.52 to 375.07)
2044	Male	1.76 (0.95 to 2.57)	249.91 (139.89 to 359.93)	269.12 (144.59 to 393.64)
2045	Male	1.84 (0.93 to 2.75)	251.28 (133.48 to 369.08)	276.73 (140.11 to 413.35)
2046	Male	1.93 (0.91 to 2.94)	252.67 (126.82 to 378.52)	284.29 (134.84 to 433.74)
2047	Male	2.01 (0.89 to 3.14)	254.07 (119.87 to 388.27)	291.47 (128.6 to 454.34)
2048	Male	2.1 (0.86 to 3.35)	255.48 (112.66 to 398.31)	298.61 (121.54 to 475.67)
2049	Male	2.2 (0.82 to 3.58)	256.91 (105.16 to 408.66)	306.06 (113.78 to 498.33)
2050	Male	2.3 (0.77 to 3.83)	258.34 (97.38 to 419.31)	313.69 (105.2 to 522.19)
2021	Female	1.28 (1.28 to 1.29)	303.83 (303.31 to 304.35)	305.18 (303.31 to 307.04)
2022	Female	1.34 (1.31 to 1.37)	304.63 (300.39 to 308.87)	306.28 (299.55 to 313)
2023	Female	1.39 (1.35 to 1.42)	305.89 (299.26 to 312.53)	307.56 (298.99 to 316.13)
2024	Female	1.44 (1.39 to 1.49)	307.22 (297.53 to 316.9)	310.69 (299.4 to 321.98)
2025	Female	1.5 (1.43 to 1.57)	308.62 (295.39 to 321.85)	314.3 (299.57 to 329.02)
2026	Female	1.56 (1.47 to 1.65)	310.11 (292.89 to 327.32)	317.58 (298.84 to 336.33)
2027	Female	1.62 (1.5 to 1.74)	311.57 (289.95 to 333.19)	319.99 (296.71 to 343.26)
2028	Female	1.69 (1.54 to 1.83)	313.03 (286.63 to 339.43)	322.46 (294.19 to 350.73)
2029	Female	1.76 (1.58 to 1.94)	314.53 (283.02 to 346.04)	325.78 (292.01 to 359.55)
2030	Female	1.84 (1.62 to 2.06)	316.1 (279.14 to 353.05)	329.6 (289.84 to 369.36)
2031	Female	1.92 (1.66 to 2.19)	317.73 (274.99 to 360.47)	333.71 (287.46 to 379.97)
2032	Female	2.01 (1.69 to 2.32)	319.32 (270.46 to 368.18)	337.95 (284.7 to 391.2)
2033	Female	2.1 (1.73 to 2.47)	320.89 (265.6 to 376.18)	342.99 (282.13 to 403.85)
2034	Female	2.2 (1.76 to 2.63)	322.49 (260.46 to 384.52)	349.21 (280.01 to 418.4)
2035	Female	2.31 (1.8 to 2.81)	324.16 (255.08 to 393.24)	356.19 (277.95 to 434.43)
2036	Female	2.42 (1.83 to 3)	325.9 (249.44 to 402.36)	363.62 (275.62 to 451.62)
2037	Female	2.53 (1.86 to 3.2)	327.61 (243.46 to 411.76)	371.16 (272.7 to 469.61)
2038	Female	2.65 (1.88 to 3.42)	329.31 (237.15 to 421.47)	379.46 (269.65 to 489.26)
2039	Female	2.78 (1.91 to 3.65)	331.03 (230.54 to 431.52)	388.91 (266.68 to 511.14)
2040	Female	2.91 (1.92 to 3.9)	332.8 (223.66 to 441.95)	399 (263.33 to 534.66)

2041	Female	3.05 (1.93 to 4.17)	334.61 (216.47 to 452.74)	409.28 (259.24 to 559.32)
2042	Female	3.19 (1.93 to 4.45)	336.38 (208.92 to 463.84)	419.29 (254.04 to 584.53)
2043	Female	3.33 (1.93 to 4.74)	338.13 (201.01 to 475.25)	429.8 (248.19 to 611.4)
2044	Female	3.49 (1.91 to 5.06)	339.9 (192.78 to 487.02)	441.28 (241.89 to 640.67)
2045	Female	3.65 (1.89 to 5.4)	341.7 (184.22 to 499.18)	453.12 (234.68 to 671.56)
2046	Female	3.81 (1.85 to 5.76)	343.54 (175.33 to 511.74)	464.78 (226.22 to 703.35)
2047	Female	3.97 (1.8 to 6.14)	345.38 (166.07 to 524.69)	475.75 (216.21 to 735.29)
2048	Female	4.14 (1.74 to 6.53)	347.23 (156.44 to 538.03)	486.5 (204.89 to 768.12)
2049	Female	4.32 (1.67 to 6.96)	349.11 (146.45 to 551.77)	497.68 (192.49 to 802.86)
2050	Female	4.5 (1.58 to 7.42)	351.01 (136.09 to 565.92)	509.08 (178.84 to 839.32)

Note: Data in parentheses are 95% confidence intervals. Count data in 100000 are presented to two decimal places. ASR, age-standardized rate; BAPC, Bayesian age-period-cohort; ADOD, Alzheimer's disease and other dementias.

Table S13. Case number and ASR prediction of DALYs for ADOD aged ≥65 years from 2022 to 2050 by BAPC models in global, by both sexes

Year	Sex	Number (000000s)	ASR (per 100,000)	Crude rate (per 100,000)
2021	Both	32.56 (32.5 to 32.61)	4601.13 (4593.08 to 4609.19)	4227.14 (4219.73 to 4234.55)
2022	Both	34.25 (33.55 to 34.95)	4610.8 (4516.25 to 4705.35)	4287.4 (4199.2 to 4375.59)
2023	Both	35.6 (34.65 to 36.56)	4641.27 (4517.17 to 4765.37)	4324.49 (4208.48 to 4440.51)
2024	Both	37.13 (35.81 to 38.45)	4671.99 (4505.79 to 4838.19)	4380.48 (4224.23 to 4536.72)
2025	Both	38.76 (36.96 to 40.56)	4704.26 (4485.97 to 4922.55)	4442.85 (4236.31 to 4649.39)
2026	Both	40.45 (38.06 to 42.83)	4738.71 (4459.64 to 5017.79)	4503.62 (4238.08 to 4769.16)
2027	Both	42.14 (39.07 to 45.22)	4773.85 (4425.87 to 5121.83)	4555.89 (4223.52 to 4888.26)
2028	Both	43.97 (40.09 to 47.85)	4809.41 (4385.47 to 5233.34)	4608.28 (4201.76 to 5014.8)
2029	Both	46.03 (41.21 to 50.84)	4845.66 (4339.28 to 5352.03)	4668.52 (4180.29 to 5156.75)
2030	Both	48.23 (42.35 to 54.11)	4883.53 (4288.35 to 5478.71)	4733.52 (4156.24 to 5310.8)
2031	Both	50.52 (43.43 to 57.61)	4923.54 (4232.94 to 5614.13)	4801.78 (4127.94 to 5475.62)
2032	Both	52.83 (44.39 to 61.27)	4964.13 (4171.33 to 5756.94)	4871.07 (4092.96 to 5649.18)
2033	Both	55.28 (45.32 to 65.23)	5005.47 (4103.91 to 5907.02)	4948.33 (4057.21 to 5839.46)
2034	Both	57.93 (46.27 to 69.6)	5047.96 (4031.16 to 6064.77)	5037.06 (4023.09 to 6051.03)
2035	Both	60.74 (47.18 to 74.31)	5092.4 (3953.61 to 6231.18)	5133.43 (3986.82 to 6280.03)
2036	Both	63.64 (47.96 to 79.31)	5139.21 (3871.24 to 6407.17)	5234.57 (3945.36 to 6523.78)
2037	Both	66.54 (48.56 to 84.51)	5187.1 (3782.49 to 6591.71)	5336.98 (3895.24 to 6778.71)
2038	Both	69.54 (49.04 to 90.05)	5236.37 (3687.52 to 6785.22)	5447.47 (3841.17 to 7053.77)
2039	Both	72.74 (49.43 to 96.05)	5287.37 (3586.43 to 6988.3)	5570.09 (3785.23 to 7354.96)
2040	Both	76.09 (49.7 to 102.48)	5340.56 (3479.28 to 7201.85)	5700.21 (3723.11 to 7677.32)
2041	Both	79.52 (49.77 to 109.27)	5396.22 (3365.75 to 7426.69)	5833.76 (3651.1 to 8016.43)
2042	Both	82.97 (49.59 to 116.36)	5453.5 (3244.69 to 7662.32)	5966.18 (3565.4 to 8366.96)
2043	Both	86.56 (49.2 to 123.92)	5512.71 (3115.92 to 7909.49)	6105.6 (3470.55 to 8740.66)
2044	Both	90.37 (48.65 to 132.09)	5574.17 (2979.24 to 8169.1)	6256.95 (3368.31 to 9145.58)

2045	Both	94.33 (47.85 to 140.81)	5638.32 (2834.38 to 8442.26)	6413.59 (3253.58 to 9573.6)
2046	Both	98.39 (46.76 to 150.03)	5705.47 (2680.85 to 8730.08)	6569.65 (3122.22 to 10017.08)
2047	Both	102.5 (45.31 to 159.68)	5775.36 (2517.74 to 9032.97)	6719.09 (2970.57 to 10467.61)
2048	Both	106.76 (43.54 to 169.97)	5848.23 (2344.45 to 9352.01)	6866.28 (2800.55 to 10932.01)
2049	Both	111.28 (41.46 to 181.1)	5924.33 (2160.33 to 9688.33)	7017.76 (2614.39 to 11421.13)
2050	Both	115.99 (38.98 to 193.01)	6004.01 (1964.65 to 10043.37)	7172.43 (2410.48 to 11934.37)
2021	Male	10.87 (10.85 to 10.9)	3769.57 (3767.27 to 3771.87)	3113.62 (3106.87 to 3120.36)
2022	Male	11.59 (11.34 to 11.83)	3780.25 (3730.44 to 3830.06)	3202.3 (3134.16 to 3270.44)
2023	Male	12.07 (11.74 to 12.4)	3804.86 (3724.65 to 3885.07)	3237.68 (3148.52 to 3326.85)
2024	Male	12.62 (12.16 to 13.08)	3829.96 (3711.23 to 3948.68)	3288.21 (3168.53 to 3407.9)
2025	Male	13.2 (12.58 to 13.82)	3855.93 (3692.37 to 4019.48)	3343.21 (3185.19 to 3501.23)
2026	Male	13.8 (12.97 to 14.62)	3883 (3668.87 to 4097.14)	3395.9 (3192.77 to 3599.04)
2027	Male	14.39 (13.33 to 15.46)	3910.17 (3639.87 to 4180.48)	3440.95 (3186.61 to 3695.3)
2028	Male	15.04 (13.7 to 16.38)	3937.58 (3606.12 to 4269.05)	3485.98 (3174.65 to 3797.31)
2029	Male	15.77 (14.1 to 17.44)	3965.49 (3568.19 to 4362.8)	3537.2 (3162.89 to 3911.52)
2030	Male	16.55 (14.5 to 18.59)	3994.25 (3526.51 to 4461.99)	3591.25 (3148.2 to 4034.3)
2031	Male	17.35 (14.89 to 19.81)	4024.15 (3481.22 to 4567.08)	3646.52 (3128.96 to 4164.08)
2032	Male	18.16 (15.22 to 21.09)	4054.21 (3431.22 to 4677.2)	3701.41 (3103.46 to 4299.36)
2033	Male	19.01 (15.55 to 22.47)	4084.55 (3376.86 to 4792.25)	3761.72 (3076.69 to 4446.75)
2034	Male	19.93 (15.88 to 23.99)	4115.42 (3318.45 to 4912.4)	3830.37 (3050.7 to 4610.04)
2035	Male	20.91 (16.19 to 25.63)	4147.14 (3256.22 to 5038.06)	3904.06 (3022.32 to 4785.81)
2036	Male	21.91 (16.46 to 27.37)	4180.01 (3190.19 to 5169.83)	3980.71 (2989.35 to 4972.06)
2037	Male	22.91 (16.65 to 29.17)	4213.2 (3119.42 to 5306.99)	4058.19 (2949.64 to 5166.74)
2038	Male	23.95 (16.81 to 31.09)	4246.85 (3044.07 to 5449.62)	4142.03 (2907 to 5377.05)
2039	Male	25.05 (16.93 to 33.17)	4281.08 (2964.24 to 5597.92)	4235.36 (2863.02 to 5607.7)
2040	Male	26.2 (17.01 to 35.39)	4316.06 (2879.95 to 5752.17)	4334.55 (2814.31 to 5854.79)
2041	Male	27.39 (17.03 to 37.75)	4351.95 (2791.07 to 5912.83)	4436.72 (2758.16 to 6115.27)
2042	Male	28.59 (16.96 to 40.22)	4388.16 (2696.96 to 6079.36)	4538.86 (2691.94 to 6385.77)

2043	Male	29.84 (16.82 to 42.86)	4424.75 (2597.64 to 6251.87)	4647.35 (2619.15 to 6675.55)
2044	Male	31.17 (16.62 to 45.73)	4461.8 (2493.1 to 6430.51)	4765.9 (2541 to 6990.81)
2045	Male	32.57 (16.34 to 48.79)	4499.38 (2383.29 to 6615.46)	4889.17 (2453.31 to 7325.03)
2046	Male	34 (15.96 to 52.04)	4537.56 (2268.09 to 6807.04)	5012.51 (2352.77 to 7672.25)
2047	Male	35.45 (15.45 to 55.45)	4576.08 (2147.15 to 7005)	5131.43 (2236.6 to 8026.26)
2048	Male	36.97 (14.83 to 59.1)	4614.98 (2020.46 to 7209.5)	5249.32 (2106.19 to 8392.45)
2049	Male	38.58 (14.1 to 63.06)	4654.3 (1887.97 to 7420.63)	5371.06 (1963.1 to 8779.02)
2050	Male	40.27 (13.23 to 67.31)	4694.08 (1749.62 to 7638.54)	5495.59 (1805.92 to 9185.27)
2021	Female	21.68 (21.65 to 21.72)	5157.98 (5155.81 to 5160.16)	5151.01 (5143.05 to 5158.98)
2022	Female	22.66 (22.2 to 23.12)	5180.24 (5114.11 to 5246.38)	5186 (5081.19 to 5290.81)
2023	Female	23.53 (22.91 to 24.15)	5217.19 (5109.25 to 5325.12)	5224.04 (5085.81 to 5362.28)
2024	Female	24.51 (23.65 to 25.38)	5254.93 (5094.12 to 5415.73)	5283.96 (5097.47 to 5470.44)
2025	Female	25.56 (24.38 to 26.74)	5294.14 (5071.89 to 5516.4)	5351.83 (5105.18 to 5598.48)
2026	Female	26.65 (25.09 to 28.21)	5335.12 (5043.55 to 5626.69)	5418.7 (5101.61 to 5735.79)
2027	Female	27.75 (25.74 to 29.76)	5375.64 (5007.09 to 5744.2)	5476.45 (5079.65 to 5873.24)
2028	Female	28.93 (26.4 to 31.47)	5416.04 (4963.64 to 5868.45)	5534.65 (5049.56 to 6019.74)
2029	Female	30.26 (27.11 to 33.4)	5456.98 (4914.29 to 5999.68)	5602.3 (5020.05 to 6184.56)
2030	Female	31.68 (27.84 to 35.52)	5499.11 (4859.78 to 6138.43)	5676.43 (4988.35 to 6364.51)
2031	Female	33.17 (28.54 to 37.8)	5542.6 (4800.12 to 6285.08)	5755.54 (4952.68 to 6558.39)
2032	Female	34.68 (29.17 to 40.18)	5585.22 (4733.03 to 6437.42)	5836.79 (4909.93 to 6763.65)
2033	Female	36.27 (29.78 to 42.76)	5627.58 (4659.38 to 6595.77)	5928.35 (4867.01 to 6989.69)
2034	Female	38 (30.4 to 45.6)	5670.43 (4580.02 to 6760.83)	6034.29 (4826.69 to 7241.89)
2035	Female	39.83 (30.99 to 48.68)	5714.41 (4495.44 to 6933.38)	6150.11 (4784.46 to 7515.76)
2036	Female	41.72 (31.51 to 51.94)	5759.68 (4405.49 to 7113.87)	6272.12 (4736.44 to 7807.8)
2037	Female	43.63 (31.91 to 55.34)	5804.34 (4308.31 to 7300.38)	6395.43 (4677.91 to 8112.95)
2038	Female	45.6 (32.23 to 58.96)	5849.02 (4204.52 to 7493.52)	6528.06 (4614.44 to 8441.68)
2039	Female	47.69 (32.5 to 62.89)	5894.25 (4094.56 to 7693.94)	6675.01 (4548.65 to 8801.37)
2040	Female	49.88 (32.68 to 67.08)	5940.38 (3978.58 to 7902.19)	6830.79 (4475.46 to 9186.11)

2041	Female	52.13 (32.74 to 71.52)	5987.41 (3856.28 to 8118.54)	6990.36 (4390.35 to 9590.37)
2042	Female	54.38 (32.63 to 76.14)	6034.04 (3726.49 to 8341.6)	7147.81 (4288.5 to 10007.12)
2043	Female	56.72 (32.39 to 81.06)	6080.73 (3589.54 to 8571.93)	7312.76 (4175.34 to 10450.18)
2044	Female	59.19 (32.03 to 86.36)	6127.85 (3445.62 to 8810.09)	7491.16 (4053.12 to 10929.19)
2045	Female	61.77 (31.51 to 92.02)	6175.66 (3294.78 to 9056.54)	7675.36 (3915.97 to 11434.74)
2046	Female	64.4 (30.8 to 97.99)	6224.21 (3136.84 to 9311.59)	7858.49 (3759.09 to 11957.89)
2047	Female	67.05 (29.86 to 104.23)	6272.94 (2971.24 to 9574.64)	8033.29 (3578.12 to 12488.47)
2048	Female	69.79 (28.71 to 110.87)	6322.07 (2798.06 to 9846.07)	8205.05 (3375.44 to 13034.65)
2049	Female	72.7 (27.35 to 118.04)	6371.72 (2617.31 to 10126.13)	8381.62 (3153.82 to 13609.41)
2050	Female	75.72 (25.75 to 125.7)	6421.99 (2428.91 to 10415.07)	8561.75 (2911.39 to 14212.11)

Note: Data in parentheses are 95% confidence intervals. Count data in 100000 are presented to two decimal places. ASR, age-standardized rate; DALYs, Disability-Adjusted Life Years; BAPC, Bayesian age-period-cohort; ADOD, Alzheimer's disease and other dementias.

Figure S1. The changes in the proportion of prevalence cases among ADOD aged over 65 years to the overall ADOD patients from 1990 to 2021.

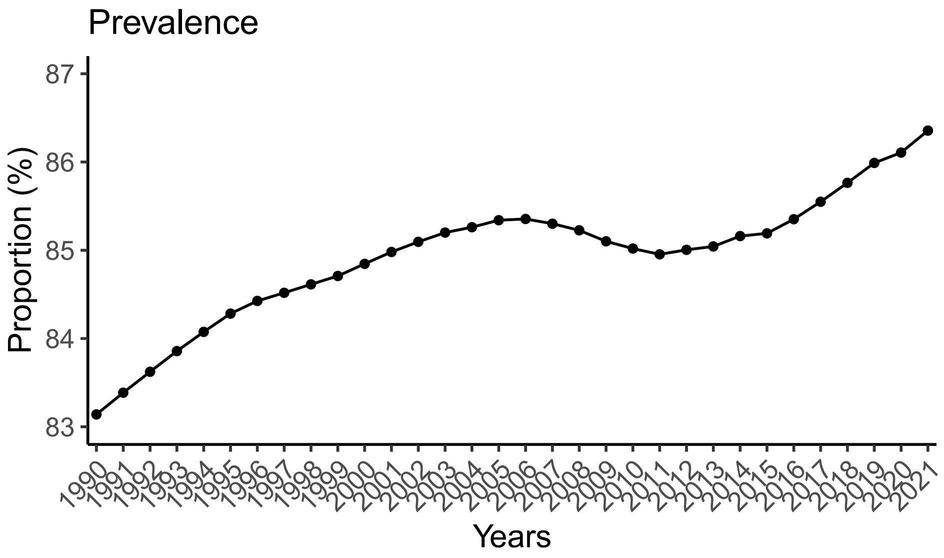

Note: ADOD, Alzheimer's disease and other dementias.

Figure S2. Temporal trend of age-standardized prevalence (A) mortality (B), and DALYs (C) for ADOD patients aged over 65 years and overall ADOD patients from 1990 to 2021.

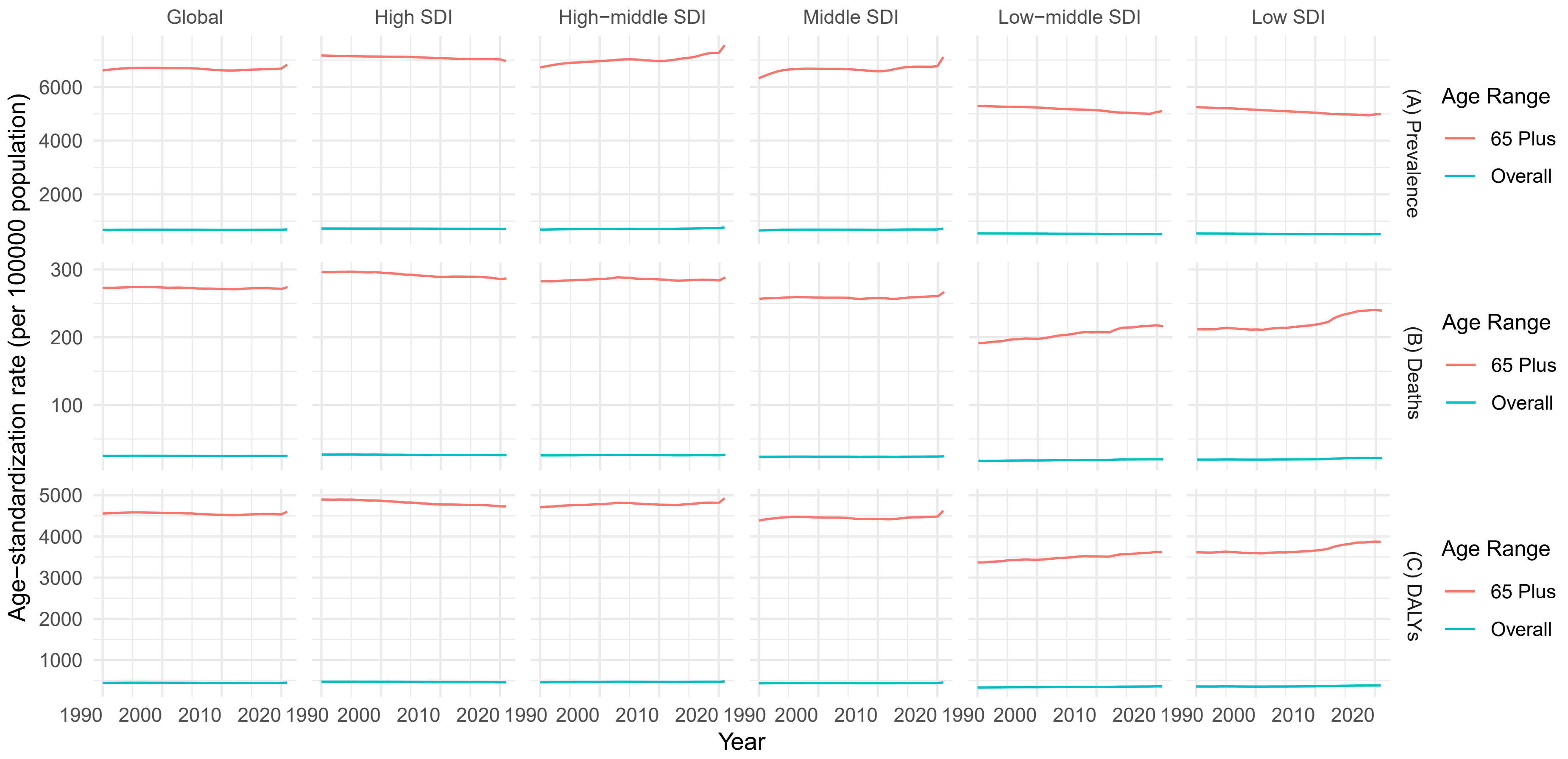

Note: ADOD, Alzheimer's disease and other dementias; DALYs, disability-adjusted life years.

Figure S3. Average annual percent changes of age-standardized prevalence, mortality, and DALYs of ADOD in patients aged over 65 years from 1990 to 2021 by sex and age.

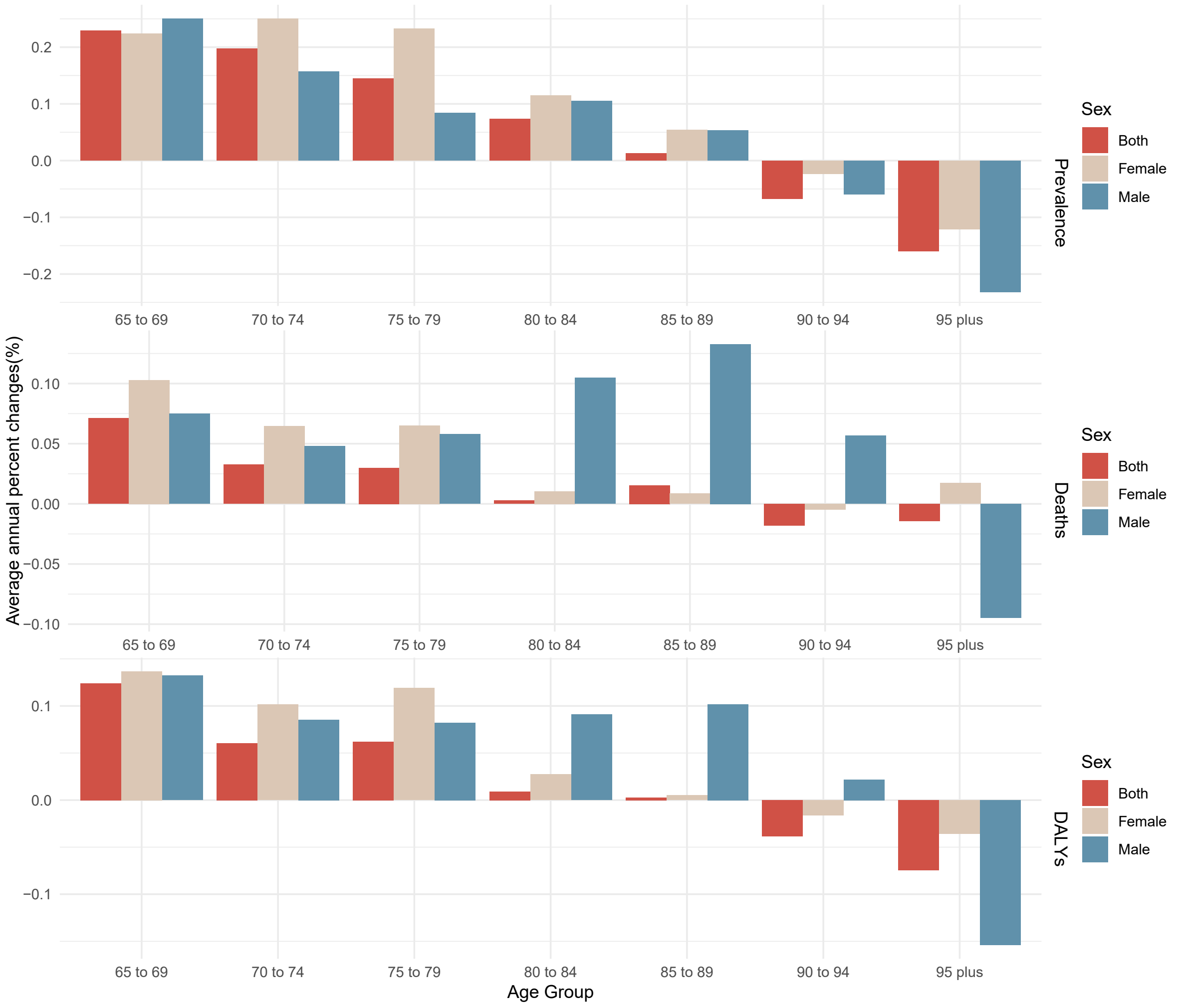

Note: ADOD, Alzheimer's disease and other dementias; DALYs, disease-adjusted life years.

Figure S4. Temporal trend of age-standardized prevalence, mortality, and DALYs of ADOD in patients aged over 65 years from 1990 to 2021 at global and socio-demographic index levels by sex.

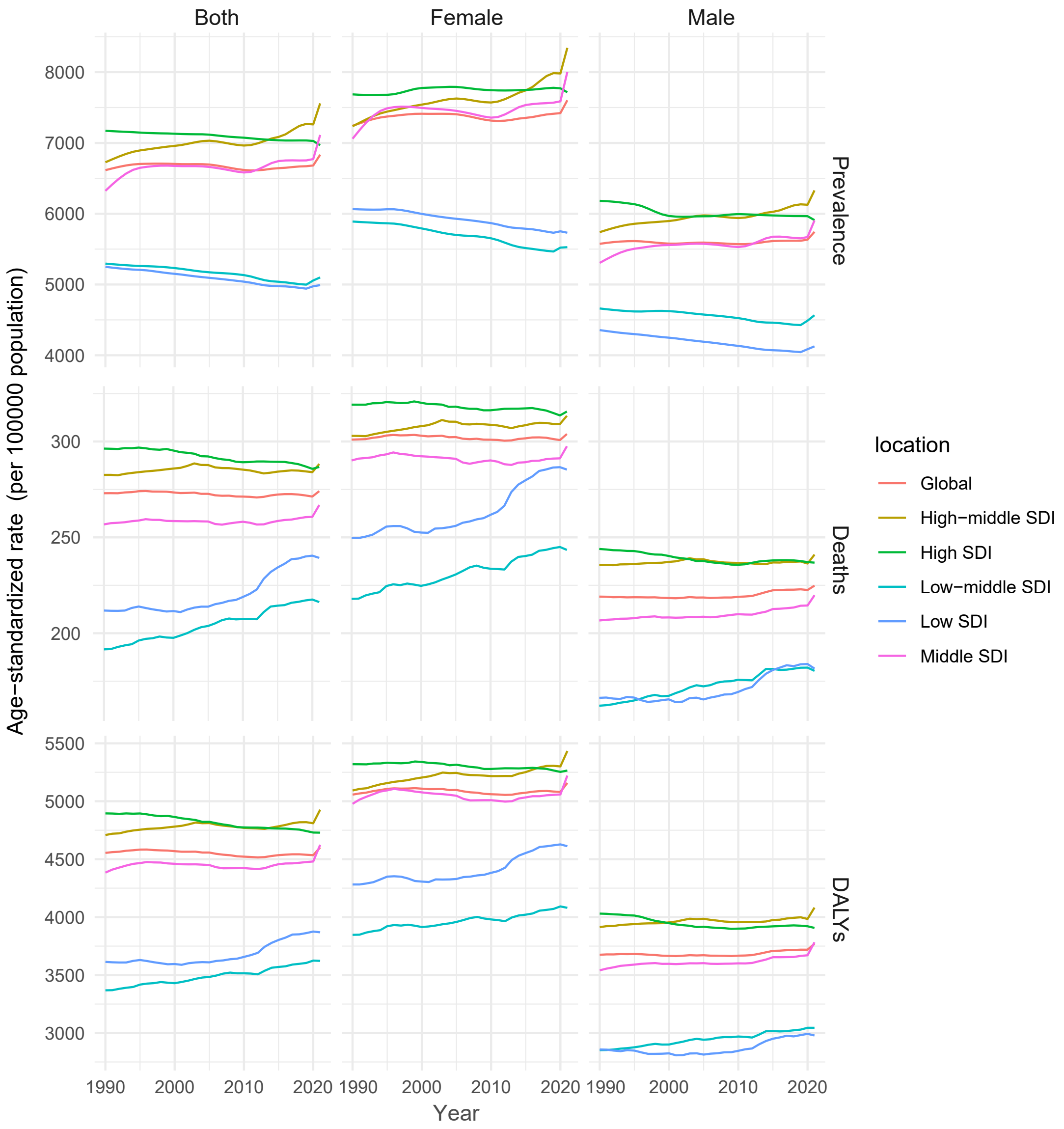

Note: ADOD, Alzheimer's disease and other dementias; SDI, socio-demographic index; DALYs, disease-adjusted life years.

Figure S5. Average annual percent changes of age-standardized prevalence, mortality, and DALYs of ADOD in patients aged over 65 years from 1990 to 2021 at socio-demographic index levels by sex.

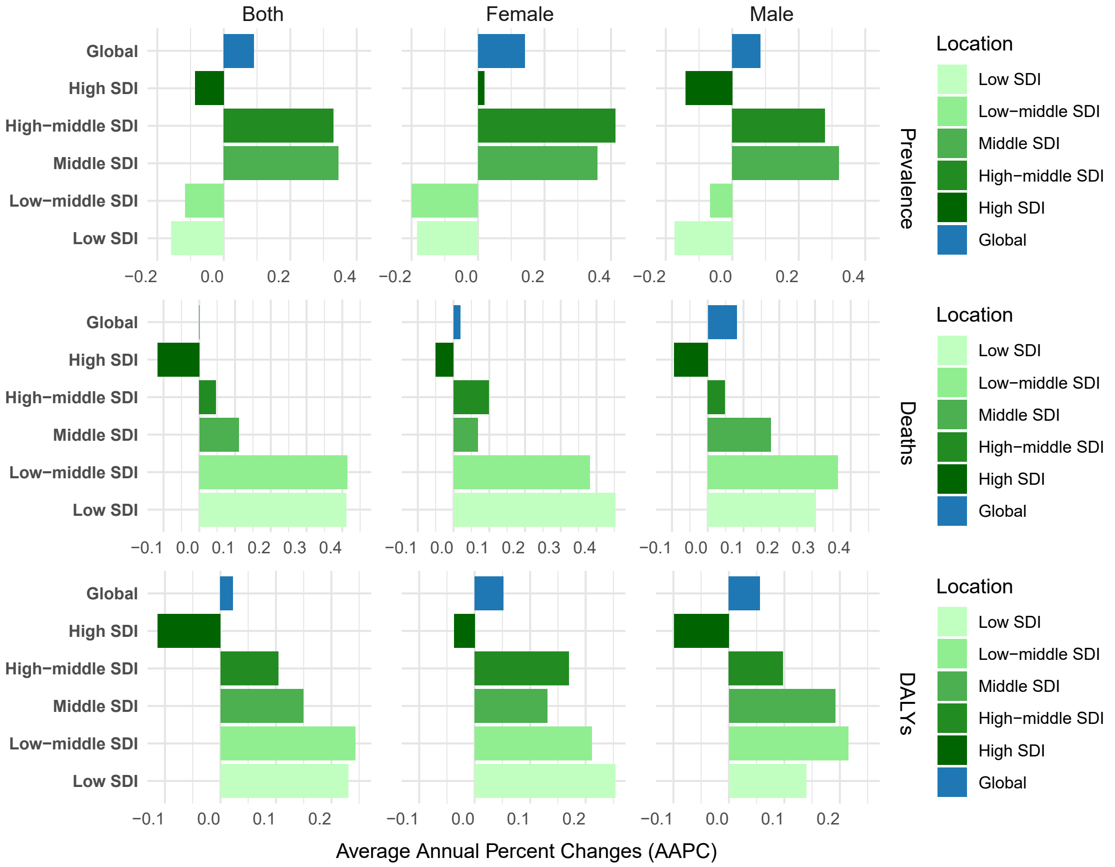

Note: ADOD, Alzheimer's disease and other dementias; SDI, socio-demographic index; DALYs, disease-adjusted life years.

Figure S6. Prevalence (A), mortality (B), and DALYs (C) rate of ADOD patients aged over 65 years from 204 countries according to the socio-demographic index in 2021

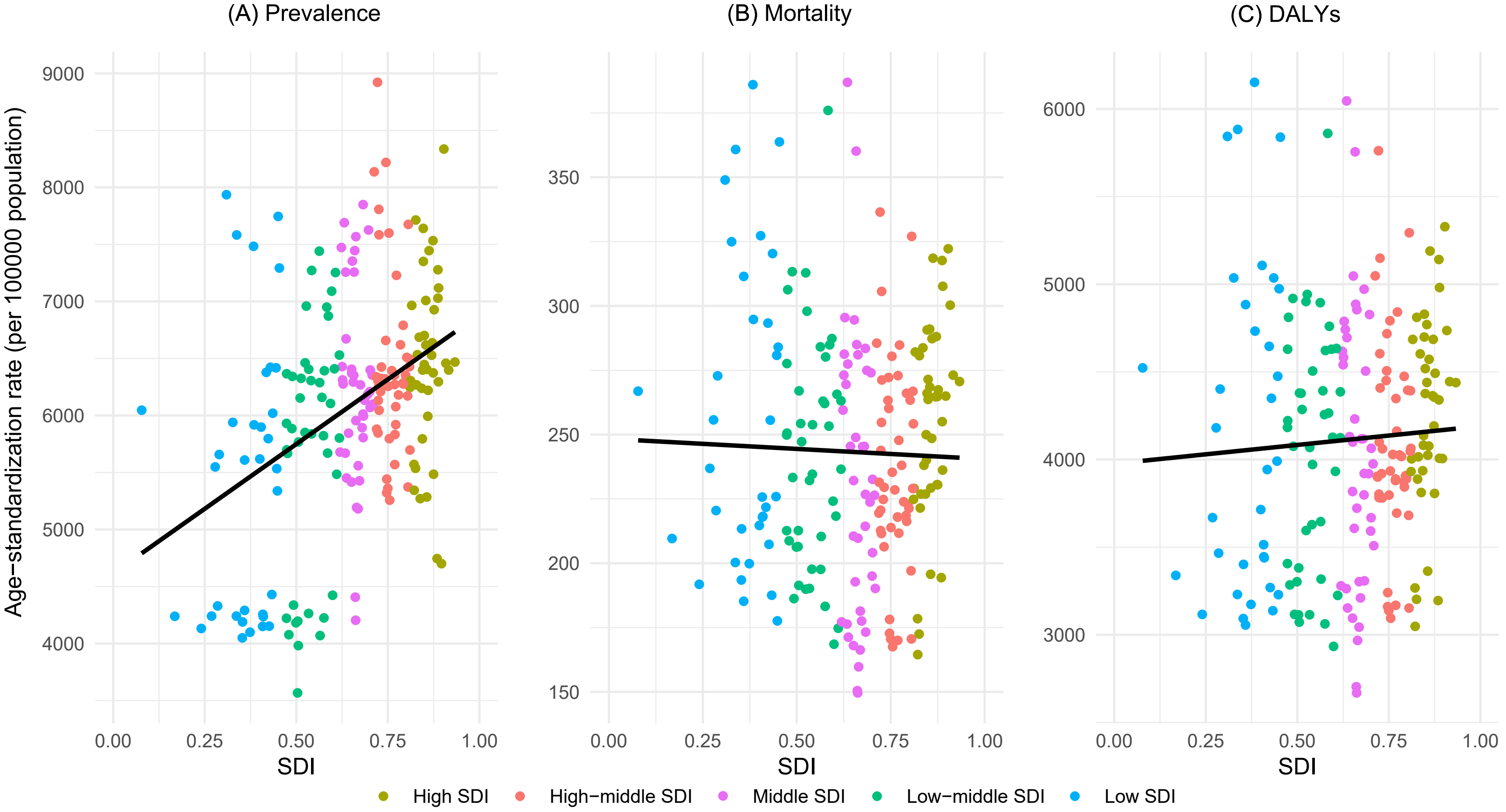

Note: ADOD, Alzheimer's disease and other dementias; SDI, socio-demographic index; DALYs, disease-adjusted life years.

Figure S7. Average annual percent changes of age-standardized prevalence (A), mortality (B), and DALYs (C) of ADOD aged over 65 years and overall ADOD patients from 1990 to 2021 at global and socio-demographic index levels.

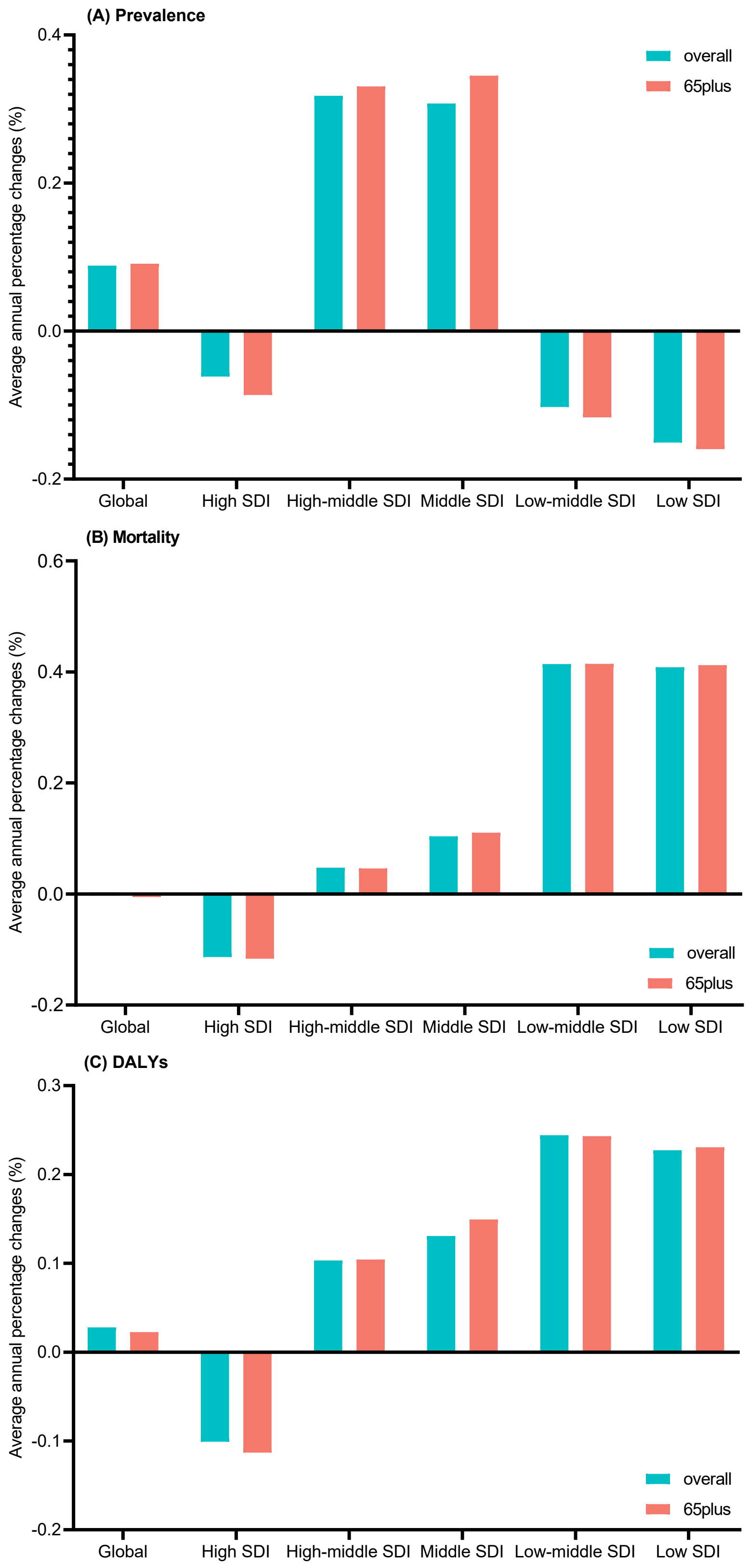

Note: ADOD, Alzheimer's disease and other dementias; SDI, socio-demographic index.

Figure S8. Average annual percent changes of age-standardized prevalence, mortality, and DALYs of ADOD patients aged over 65 years from 1990 to 2021 at regions levels

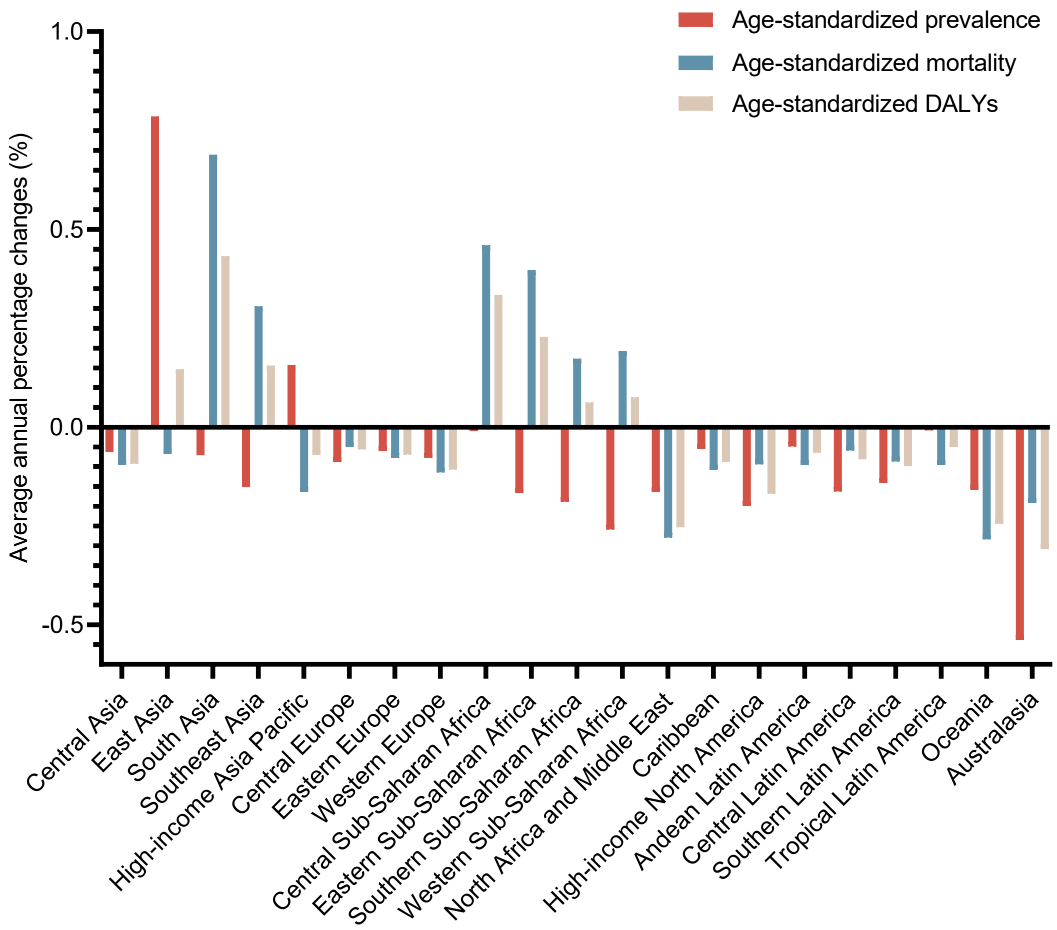

Figure S9. Average annual percent changes of age-standardized prevalence, mortality, and DALYs of ADOD patients aged over 65 years from 1990 to 2021 at regions levels by sex

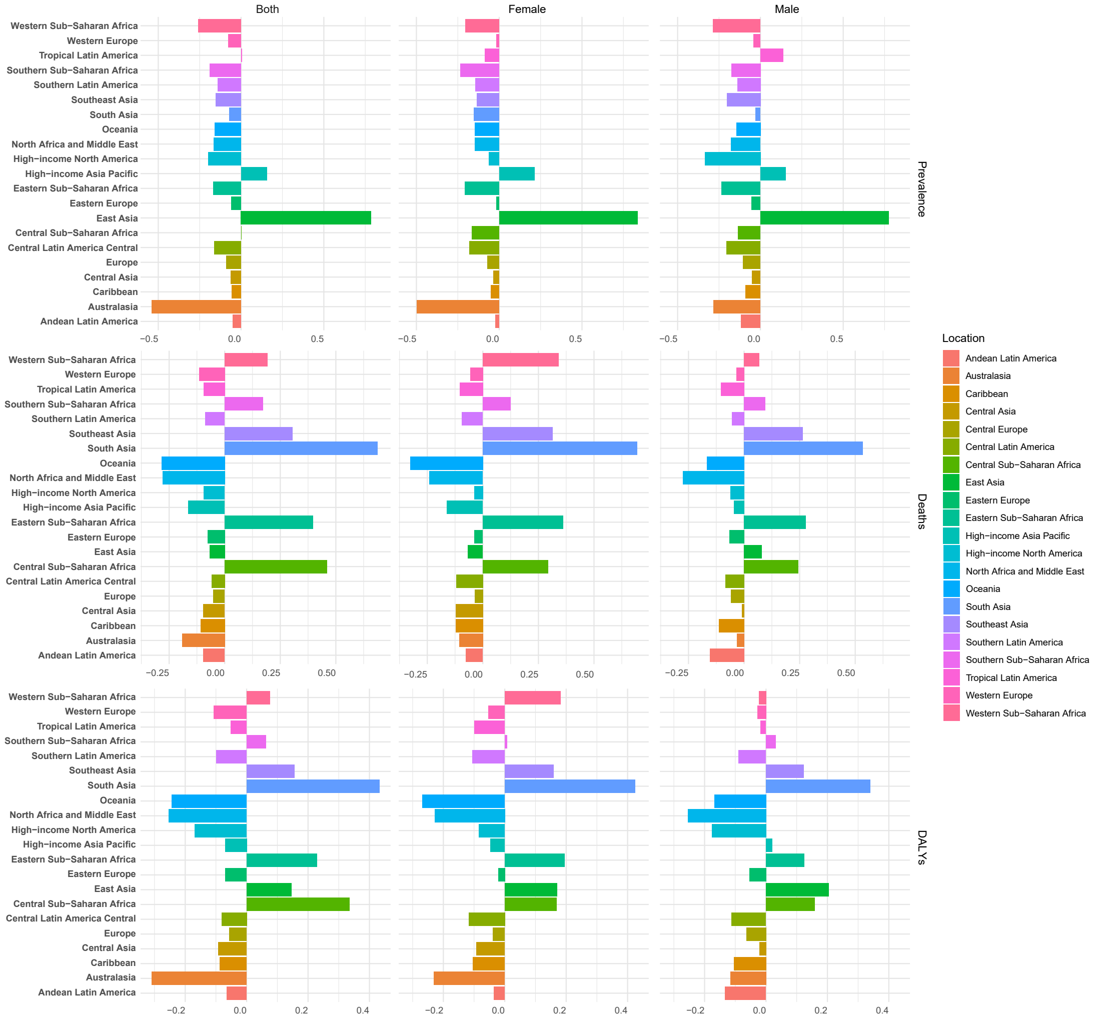

Note: ADOD, Alzheimer's disease and other dementias; SDI, socio-demographic index; DALYs, disease-adjusted life years.

Figure S10. Association between age-standardised mortality of ADOD among people aged ≥65 years and SDI

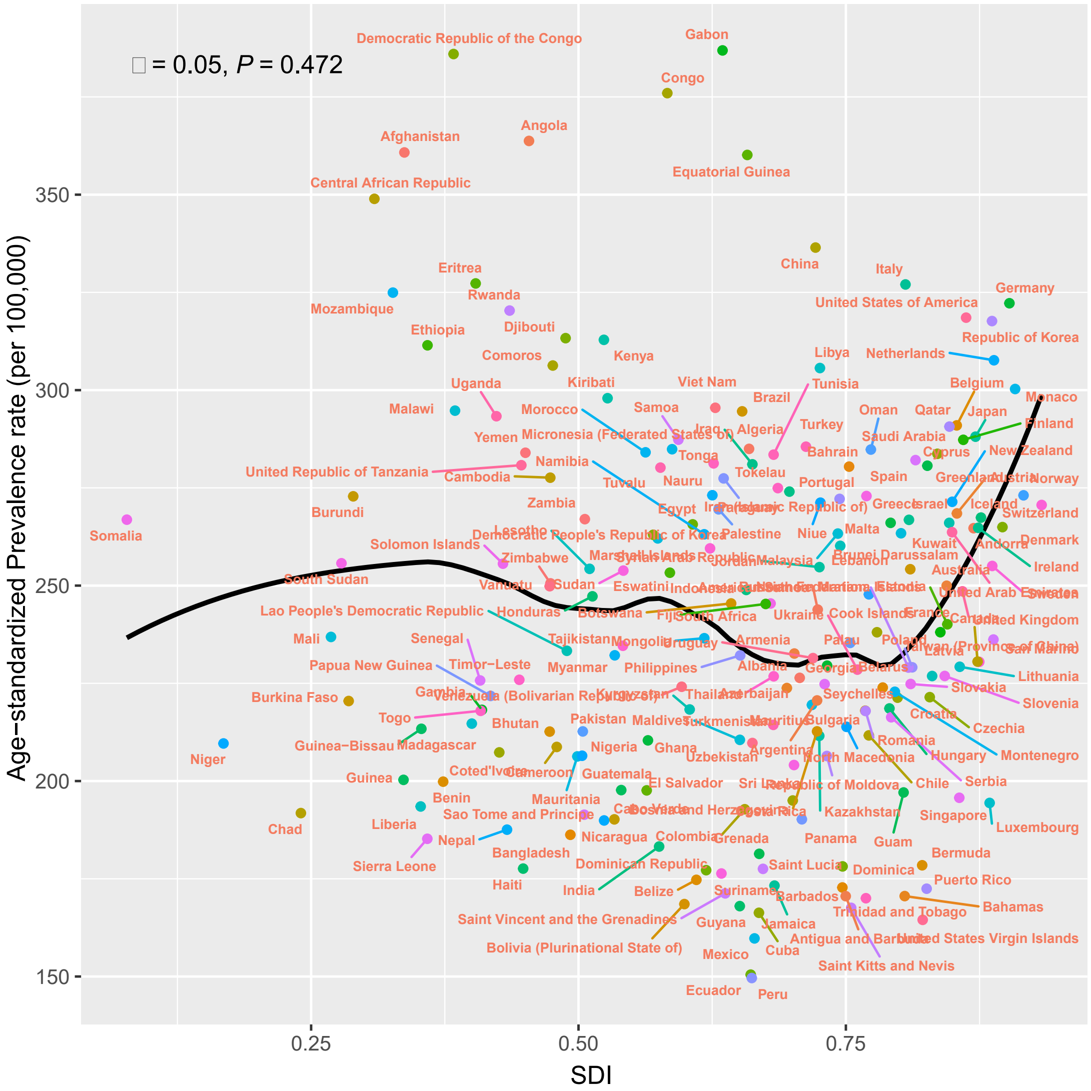

Note: ADOD, Alzheimer's disease and other dementias; SDI, socio-demographic index.

Figure S11. Association between age-standardised DALYs of ADOD among people aged ≥65 years and SDI

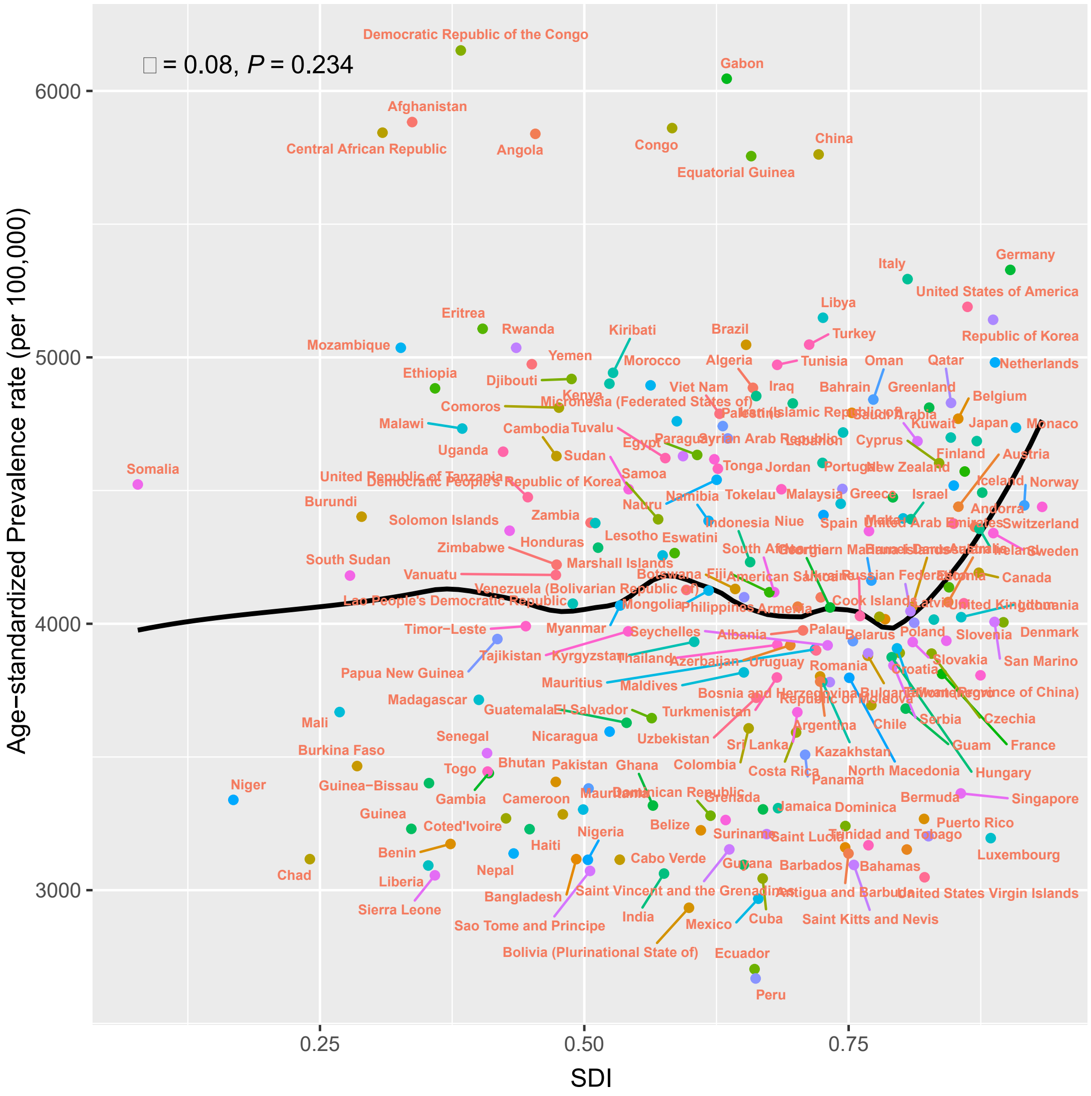

Note: ADOD, Alzheimer's disease and other dementias; SDI, socio-demographic index; DALYs, disease-adjusted life years.

STROBE Statement

STROBE Statement—checklist of items that should be included in reports of observational studies

	Item No.	Recommendation	Page No.
**Title and abstract**	1	(*a*) Indicate the study's design with a commonly used term in the title or the abstract	1
		(*b*) Provide in the abstract an informative and balanced summary of what was done and what was found	3-4
**Introduction**			
Background/rationale	2	Explain the scientific background and rationale for the investigation being reported	6
Objectives	3	State specific objectives, including any prespecified hypotheses	6
**Methods**			
Study design	4	Present key elements of study design early in the paper	6
Setting	5	Describe the setting, locations, and relevant dates, including periods of recruitment, exposure, follow-up, and data collection	6-7
Participants	6	*Cohort study*—Give the eligibility criteria, and the sources and methods of selection of participants. Describe methods of follow-up	6 and "Methods" section of the Appendix
		Cohort study—For matched studies, give matching criteria and number of exposed and unexposed	NA
Variables	7	Clearly define all outcomes, exposures, predictors, potential confounders, and effect modifiers. Give diagnostic criteria, if applicable	6-8 and "Methods" section of the Appendix
Data sources/ measurement	8*	For each variable of interest, give sources of data and details of methods of assessment (measurement). Describe comparability of assessment methods if there is more than one group	8 and "Methods" section of the Appendix
Bias	9	Describe any efforts to address potential sources of bias	16
Study size	10	Explain how the study size was arrived at	NA

Continued on next page

Quantitative variables	11	Explain how quantitative variables were handled in the analyses. If applicable, describe which groupings were chosen and why	8 and "Methods" section of the Appendix
Statistical methods	12	(*a*) Describe all statistical methods, including those used to control for confounding	7-8
		(*b*) Describe any methods used to examine subgroups and interactions	7-8
		(*c*) Explain how missing data were addressed	"Methods" section of the Appendix
		(*d*) *Cohort study*—If applicable, explain how loss to follow-up was addressed	NA
		(*e*) Describe any sensitivity analyses	7-8
**Results**			
Participants	13*	(a) Report numbers of individuals at each stage of study—eg numbers potentially eligible, examined for eligibility, confirmed eligible, included in the study, completing follow-up, and analysed	8
		(b) Give reasons for non-participation at each stage	"Methods" section of the Appendix
		(c) Consider use of a flow diagram	"Methods" section of the Appendix
Descriptive data	14*	(a) Give characteristics of study participants (eg demographic, clinical, social) and information on exposures and potential confounders	8
		(b) Indicate number of participants with missing data for each variable of interest	NA
		(c) *Cohort study*—Summarise follow-up time (eg, average and total amount)	NA
Outcome data	15*	*Cohort study*—Report numbers of outcome events or summary measures over time	NA
Main results	16	(*a*) Give unadjusted estimates and, if applicable, confounder-adjusted estimates and their precision (eg, 95% confidence interval). Make clear which confounders were adjusted for and why they were included	8-14
		(*b*) Report category boundaries when continuous variables were categorized	8-14
		(*c*) If relevant, consider translating estimates of relative risk into absolute risk for a meaningful time period	NA

Continued on next page

Other analyses	17	Report other analyses done—eg analyses of subgroups and interactions, and sensitivity analyses	eTables 1-13 and eFigures 1-11
**Discussion**			
Key results	18	Summarise key results with reference to study objectives	14
Limitations	19	Discuss limitations of the study, taking into account sources of potential bias or imprecision. Discuss both direction and magnitude of any potential bias	16
Interpretation	20	Give a cautious overall interpretation of results considering objectives, limitations, multiplicity of analyses, results from similar studies, and other relevant evidence	14-15
Generalisability	21	Discuss the generalisability (external validity) of the study results	16
**Other information**			
Funding	22	Give the source of funding and the role of the funders for the present study and, if applicable, for the original study on which the present article is based	17

*Give information separately for cases and controls in case-control studies and, if applicable, for exposed and unexposed groups in cohort and cross-sectional studies.

Note: An Explanation and Elaboration article discusses each checklist item and gives methodological background and published examples of transparent reporting. The checklist is best used in conjunction with this article (freely available on the Web sites of PLoS Medicine at http://www.plosmedicine.org/, Annals of Internal Medicine at http://www.annals.org/, and Epidemiology at http://www.epidem.com/). Information on the STROBE Initiative is available at www.strobe-statement.org.
